# Comparative plastomes of five *Psittacanthus* species: genome organization, structural features, and patterns of pseudogenization and gene loss

**DOI:** 10.1093/aobpla/plaf032

**Published:** 2025-06-24

**Authors:** Saddan Morales-Saldaña, Andrea I Barraza-Ochoa, Emanuel Villafán, Antonio Acini Vásquez-Aguilar, Santiago Ramírez-Barahona, Enrique Ibarra-Laclette, Juan Francisco Ornelas

**Affiliations:** Red de Biología Evolutiva, Instituto de Ecología, A.C. (INECOL), Carretera Antigua a Coatepec No. 351, El Haya, Xalapa, Veracruz 91073, Mexico; Red de Biología Evolutiva, Instituto de Ecología, A.C. (INECOL), Carretera Antigua a Coatepec No. 351, El Haya, Xalapa, Veracruz 91073, Mexico; Red de Estudios Moleculares Avanzados, Instituto de Ecología, A.C. (INECOL), Carretera Antigua a Coatepec No. 351, El Haya, Xalapa, Veracruz 91073, Mexico; Red de Biología Evolutiva, Instituto de Ecología, A.C. (INECOL), Carretera Antigua a Coatepec No. 351, El Haya, Xalapa, Veracruz 91073, Mexico; Departamento de Botánica, Instituto de Biología, Universidad Nacional Autónoma de México (UNAM), Circuito Exterior s/n, Ciudad de México 04510, Mexico; Red de Estudios Moleculares Avanzados, Instituto de Ecología, A.C. (INECOL), Carretera Antigua a Coatepec No. 351, El Haya, Xalapa, Veracruz 91073, Mexico; Red de Biología Evolutiva, Instituto de Ecología, A.C. (INECOL), Carretera Antigua a Coatepec No. 351, El Haya, Xalapa, Veracruz 91073, Mexico; Phenome, Genome & Environment

**Keywords:** gene loss, Loranthaceae, parasitism, plastome degradation, pseudogenes

## Abstract

The evolution of heterotrophic lifestyle entails varying degrees of plastome degradation. Yet, the evolutionary trajectory of plastome degradation associated with parasitism remains poorly explored in hemiparasites. We sequenced, assembled, and annotated the complete plastomes of five species of *Psittacanthus* mistletoes. In addition, publicly available plastomes of 58 species in Loranthaceae were obtained and re-annotated for phylogenetic and comparative analyses. We used a comparative phylogenetic approach to evaluate whether patterns of pseudogenization and gene loss differ among lineages of hemiparasites in Loranthaceae. Gene order was highly conserved, with higher sequence similarity and structural conservation between closely related *Psittacanthus* species but with considerable plastome size variation (from 121 238 to 125 427 bp). The expansion and contraction at the borders of inverted repeats (IRs) and intergenic regions variation greatly contribute to size variations among *Psittacanthus* plastomes. Phylogenetic analysis of plastomes of 60 species in Loranthaceae including 5 *Psittacanthus* species of the previously unsampled tribe Psittacantheae was largely congruent with previous phylogenetic studies. The loss of most of the *ndh* complex (10 out of 11 genes), *rpl32*, *rps15*, and *rps16* genes, was identified in all studied *Psittacanthus* species. Also, the loss and pseudogenization of *rpl33* and *rpl36* genes in *Psittacanthus* were uncommon in other Loranthaceae species. The structural variation uncovered in *Psittacanthus* plastomes reveals that, despite high synteny, significant size variation exists among species. This variation can be attributed to processes such as variations in the length of intergenic regions and the expansion/contraction of IR borders, traits that have been comparatively understudied in earlier Loranthaceae works.

## Teaser

Despite the high conservation of gene order (synteny) or collinearity observed across different Psittacanthus chloroplast genomes (plastomes), significant genome size variation exist among species. The genome size differences between species likely result from processes such as expansions or contractions in intergenic regions and inverted repeat borders. Furthermore, our comparative-phylogenetic analysis of plastomes from hemiparasitic plants revealed distinctive and convergent degradation patterns in Psittacanthus, including gene loss and pseudogenization, which differ from patterns observed in plastomes of other hemiparasites in Loranthaceae.

## Introduction

Land plants’ chloroplast genomes (plastomes) have highly conserved structures and organization, and multiple copies, meaning that the target genes are expressed at high levels (Raubeson and Jansen 2005, Verma and Daniel 2007). Thousands of complete chloroplast genomes have been sequenced, characterized, and used as a source of molecular markers (Li et al. 2020, 2022) and barcoding identification (Li et al. 2021a, Jiang et al. 2023) since the first successfully sequenced complete chloroplast genomes from the liverwort *Marchantia polymorpha* L. and the angiosperm *Nicotiana tabacum* L. (Ohyama et al. 1986, Shinozaki et al. 1986). These genomes have enhanced our understanding of plant evolution (Civáň et al. 2014, Ruhfel et al. 2014, Zhao et al. 2019) and have made significant contributions to phylogenetics (Carbonell-Caballero et al. 2015, Li et al. 2021b, Tyszka et al. 2023).

In general, the chloroplast genome comprises a single circular molecule with a quadripartite structure, including two copies of an inverted repeat (IR) region that separate the large and small single-copy (LSC and SSC) regions (Bock 2007). Typically, angiosperm chloroplast genomes range from 115 to 165 kb in size, harbouring between 101 and 118 unique genes that include ∼80 protein-coding genes (PCGs), ∼30 transfer RNA (tRNA) genes, and four ribosomal RNA (rRNA) genes (Bock 2007, Qu et al. 2023). Chloroplast genomes are highly conserved in their organization, gene order, and gene content. However, in plants that have lost the ability to photosynthesize, such as some parasitic species, chloroplast genomes show structural changes, gene loss, and high rates of pseudogenization (Wolfe et al. 1992a, Bromham et al. 2013, Petersen et al. 2015, Frailey et al. 2018, Schneider et al. 2018, Zhang et al. 2022, Banerjee and Stefanović 2023, Sánchez-Puerta et al. 2023).

Parasitism in angiosperms is considered to have at least 12 independent origins, and most parasitic species (c. 4528 species) are included in the family Orobanchaceae and the order Santalales (Nickrent 2020). Since the first complete chloroplast genome sequenced from a parasitic plant *Epifagus virginiana* (L.) W.P.C. Barton (Wolfe et al. 1992b), and despite their large known diversity, <10% of chloroplast genomes have been sequenced from parasitic plants. Santalales contains the largest number of genera (179) and species (2428) among the 12 parasitic plant lineages (Nickrent 2020). Also, this order contains the widest array of nutritional modes, including autotrophic nonparasites (13 genera/71 species), holoparasites (17/45), and hemiparasites (149/2312) (Nickrent 2020), as well as a variety of plant habits such as trees, shrubs, annual and perennial herbs, and aerial parasites. This makes Santalales an important model for the study of the evolution of parasitism in plants and its effects on genome reorganization. However, only 226 verified chloroplast genomes have been reported from 94 species in Santalales [c. 4% of the species in the order; estimates obtained from data available in GenBank (accessed 29 May 2024)], which highlights the need to continue generating genomic resources for the most diverse group of parasitic plants.

While holoparasitic plants obtain all nutrients from their host and show a complete loss of photosynthetic ability, hemiparasitic plants possess varying rates of photosynthetic ability (Westwood et al. 2010). Early comparative studies of complete chloroplast genomes from Santalales [(one *Osyris* shrub species (Santalaceae) and three hemiparasitic *Viscum* species (Viscaceae) (Petersen et al. 2015)] reported that chloroplast genomes were 10%–22% reduced in size relative to the chloroplast genome of the nonparasitic *Vitis* (Vitaceae), showing rearrangements in the borders of the IRs. Also, Petersen et al. (2015) found that several PCGs (*matK*, *infA*, *ccsA*, *rpl33*, and all 11 *ndh* genes) as well as 2 tRNA genes (*trnG*-*UCC* and *trnV*-*UAC*) have been pseudogenized or completely lost. Subsequent studies in hemiparasitic Santalales reported changes in selective pressures and gene content in plastomes usually characterized by pseudogenization or loss of the *ndh* complex genes, as well as of *rpl16*, *rpl32*, *rpoC2*, *rps15*, *rps16*, *psaI*, *psbZ*, *ycf1*, *ycf2*, and *ycf15* genes. Thereby, hemiparasitic Santalales chloroplast genomes encode between 80 and 104 unique genes (Li et al. 2017, Shin and Lee 2018, Liu et al. 2019, 2021, Nie et al. 2019, Chen et al. 2020, Guo et al. 2020, 2021, Nickrent et al. 2021, Su et al. 2021, Al-Juhani et al. 2022, Darshetkar et al. 2023, Tang et al. 2024, Edlund et al. 2025).

To better understand trends of modifications in plastome evolution while still maintaining photosynthetic function, it would be informative to have complete chloroplast genome sequences of fully photosynthetic hemiparasites in Santalales from the New World (Nickrent and García 2009). The single complete chloroplast genome reported for the Psittacantheae tribe (*Psittacanthus schiedeanus*: Loranthaceae; Ibarra-Laclette et al. 2022, Morales-Saldaña et al. 2024) revealed the *trnV*-*UAC* gene for the first time in the Loranthaceae family. With at least 110 species, *Psittacanthus* Mart. is the most species-rich genus in Loranthaceae (Kuijt 2009, Dettke and Caires 2021). Loranthaceae contains the largest number of species in the Santalales, with over 1000 species distributed in 76 genera classified into 5 tribes: Nuytsiae, Gaiadendreae, Elytrantheae, Lorantheae, and Psittacantheae, distributed in tropical and subtropical regions of the Americas, Africa, Asia, and Australia, with a few species extending to the temperate zones in Europe and East Asia (Vidal-Russell and Nickrent 2008, Nickrent et al. 2010, Liu et al. 2018). Here, we sequenced and assembled for the first time the complete plastomes of five hemiparasitic *Psittacanthus* species, half of the *Psittacanthus* species distributed in Mexico: *Psittacanthus auriculatus*, *Psittacanthus palmeri*, *Psittacanthus rhynchanthus*, *Psittacanthusschiedeanus*, and *Psittacanthus sonorae* using two organelle assemblers with Illumina reads obtained from leaf samples. Our objectives were: (i) to assess structural variations and gene loss patterns among *Psittacanthus* plastomes; (ii) reconstructing plastome phylogeny to assess pseudogenization and gene loss in Loranthaceae using ancestral state reconstruction; and (iii) to assess the phylogenetic informativeness (PI) of plastome genes to solve conflictive phylogenetic relationships at both shallow and deep nodes in Loranthaceae. This study is the first to investigate plastome variation, phylogenetic utility, pseudogenization, and gene loss patterns in *Psittacanthus* and sets the stages for future studies in the genus.

## Materials and methods

### Taxon sampling

We collected and preserved in silica gel young leaves from individual plants of five *Psittacanthus* species in Mexico during the 2020–2 flowering seasons: *P. auriculatus* Eichler, *P*. *palmeri* (S. Watson) Barlow & Wiens, *P*. *rhynchanthus* (Benth.) Kuijt, *P*. *schiedeanus* (Cham. & Schltdl.) G. Don, and *P*. *sonorae* (S. Watson) Kuijt. This set of species comprises around 50% of the *Psittacanthus* species distributed in Mexico, including both range-restricted and widely distributed species, species parasitizing one or several host species, and species occurring only in one habitat type or in several habitat types. Based on previous phylogenetic studies, the sampled species are scattered along the phylogenetic tree and show diverse phylogenetic affinities among them (Ornelas et al. 2024). Therefore, we considered these five species to be ecologically and phylogenetically representative of the genus. Voucher specimens were deposited at the XAL herbarium of the Instituto de Ecología A.C. (INECOL). Geographic information of localities sampled and voucher information of the *Psittacanthus* species included in the study is summarized in Supplementary Table S1.

### Chloroplast DNA extraction, genome sequencing, and assembly

Total genomic DNA was extracted from the preserved silica-gel-dried leaf tissue samples using a DNeasy Plant Mini kit (Qiagen, Valencia, CA, USA) following the manufacturer's protocol. The purified genomic DNA was used to prepare a paired-end (PE) Illumina sequencing library using Illumina Hi-Seq PE100 technology (Illumina, Inc., San Diego, CA, USA) following the manufacturer's guidelines.

Raw Illumina reads were first filtered using Trimmomatic v.0.38 (Bolger et al. 2014) to remove adapter sequences and low-quality reads with parameters LEADING:5 TRAILING:0 SLIDINGWINDOW:4:20 MINLEN:75. The complete plastome of each sample was *de novo* assembled using NOVOPlasty v.4.0 (Dierckxsens et al. 2017), with the trimmed reads and default parameters settings. However, we failed to assemble the chloroplast genomes of *P*. *auriculatus* and *P*. *rhynchanthus*. To correct this, we employed Get Organelle (Jin et al. 2020) with the settings -w 95 -R 20 -k 21,35,45,55,65,75 -F embplant_pt parameters for *P*. *auriculatus* and the parameters -w 65 -R 30 -k 35,45,55,65,75,85,95,105,115 -F embplant_pt for *P*. *rhynchanthus*. The resulting complete sequences were checked using Geneious Prime v.2023.2.1 (Kearse et al. 2012).

### Genome annotation

All assembled plastomes were annotated using GeSeq (Tillich et al. 2017, Chan and Lowe 2019). Additionally, all chloroplast genomes were annotated using the Plastid Genome Annotator (PGA) with default settings (Qu et al. 2019) and using the plastomes of the following species: *Elytranthe albida*, *Helicanthes elasticus*, *Loranthus odoratus*, *Macrosolen bibracteolatus*, and *Nuytsia floribunda*. GenBank accession numbers and reference information of these plastomes are provided in Supplementary Table S2. The start and stop codons of each PCG were manually checked and adjusted, whereas the PCGs with one or more frameshift mutations or premature stop codons were annotated as pseudogenes. The circular genome map was drawn using the OGDRAW programme (https://chlorobox.mpimp-golm.mpg.de/OGDraw.html; Greiner et al. 2019). All newly and fully annotated plastomes were deposited in the NCBI GenBank database under the accession numbers listed in Table 1 and Supplementary Table S2.

**Table 1.** Plastome attributes for the studied *Psittacanthus* species.

	*Psittacanthus auriculatus*	*Psittacanthus palmeri*	*Psittacanthus schiedeanus*	*Psittacanthus rhynchanthus*	*Psittacanthus sonorae*
Accession number	PP236145	PP236144	OR701826	PP579889	PP313080
Average read depth	5599.25×	1803.11×	6701.48×	7449.58×	3464×
Plastome size (bp)	121 238	122 407	122 586	120 345	125 427
Coding regions	75 427 (62.2%)	76 012 (62%)	74 573 (60.8%)	75 717 (63%)	74 391 (59.3%)
Noncoding regions	45 811 (37.7%)	46 395 (37.9%)	48 013 (39.2%)	44 628 (37%)	51 036 (40.6%)
LSC size (bp)	72 322	71 489	72 507	72 087	73 497
SSC size (bp)	7646	6080	7513	8672	6124
IR size (bp)	20 635	22 419	21 283	19 793	22 903
GC (%)	36.7	36.6	36.9	36.4	36.7
Unique PCG	66	66	65	66	65
tRNA	26	26	27	27	27
rRNA	4	4	4	4	4
Unique genes	96	96	96	97	96
Total genes	115	113	113	112	112

### Chloroplast genome comparison and sequence divergence

We made comparisons using the assembled plastomes that included the previously published plastome of *P. schiedeanus* (Morales-Saldaña et al. 2024; Table 1; Supplementary Table S2) for several attributes, including gene number, gene content, gene order, guanine and cytosine (GC) content, as well as LSC, SSC, and IR size in Geneious Prime v.2023.2.1 (Kearse et al. 2012). The numbers of shared genes among different *Psittacanthus* plastomes were visualized using the heatmap2 function in ‘ggplot2’ package in R (Wickham 2016). To assess sequence divergence and to detect regions with elevated mutation rates among *Psittacanthus* plastomes, nucleotide diversity (*π*) was calculated in two datasets (PCGs and noncoding regions). Multiple sequence alignment for each dataset was performed using MAFFT v.7 (Katoh et al. 2019) and analysed independently by sliding window computation of nucleotide diversity (*π*) with a window size of 500 bp and a step size of 100 bp in DnaSP v.6.12.03 (Rozas et al. 2017). IRScope (https://irscope.shinyapps.io/irapp/; Amiryousefi et al. 2018) was used to analyse contractions and expansions of the repeat junctions across the five *Psittacanthus* plastomes. Finally, to visualize the conservation of gene order among chloroplast genomes, we generated a synteny plot using the pyGenomeViz v.0.2.1 package, employing the pgv-mmseqs mode and setting an identity threshold of 50% (https://github.com/moshi4/pyGenomeViz).

### Phylogenetic analysis

To carry out the phylogenetic analyses, we employed the chloroplast genome sequences of the newly reported *Psittacanthus* species in this paper, as well as plastomes of Loranthaceae previously verified and available on NCBI GenBank until May 2024 (Supplementary Table S2). All downloaded plastomes were reannotated, compared, and analysed to refine the missing and dubious gene annotations. Sixty PCGs shared by all Loranthaceae plastomes were extracted and aligned individually using MAFTT v.7 (Katoh et al. 2019) and concatenated into a supermatrix. The phylogenetic tree was inferred by maximum likelihood (ML) in RaxML v.8.2.12 (Stamatakis 2014) using the best-fitting model (GTR + I + G4; Supplementary Table S3) estimated from ModelTest-NG 0.1.7 (Darriba et al. 2020) by the Akaike Information Criterion (AIC), Bayesian Information Criterion (BIC), and corrected Akaike Information Criterion (AICc) methods with branch support assessed using 1000 fast bootstraps. MrBayes v.3.2.7a (Ronquist and Huelsenbeck 2003) was used for the Bayesian inference analysis. The Markov chain Monte Carlo algorithm was run for 2 000 000 generations and sampled every 500 generations. The average standard deviations of split frequencies for each of the datasets were below 0.01. Trees resulting from the first 25% of generations were discarded as burn-in, and then, posterior probability (PP) values were estimated. *Erythropalum scandens* (Erythropalaceae, Santalales), a nonparasitic woody climber and one of the first lineages to diverge within Santalales (Nickrent et al. 2010), and *Schoepfia jasminodora* (Schoepfiaceae, Santalales), a root parasite and more closely related to Loranthaceae, were used as outgroups (Supplementary Table S2).

### Loranthaceae informativeness profiles

We compared the performance of the 60 PCGs for resolving Loranthaceae relationships in terms of PI profiles with PhyDesign (López-Giráldez and Townsend 2011) using HyPhy substitution rates algorithm recommended for DNA sequences (Pond et al. 2005). The recovered ML tree was calibrated with an arbitrary timescale where tips were assigned to time 0 and root to 1 (Townsend 2007) and then converted to rooted ultrametric tree using the ‘chronos’ function in the *ape* package (Paradis et al. 2004) implemented in R Studio v.4.3.0 (R Core Team 2023). The converted relative-time ultrametric tree and alignment partitioned of concatenated 60 PCGs were used as input files in PhyDesign to calculate PI with default settings. Finally, we selected the most informative genes, and we conducted phylogenetic analysis for each gene to assess their usefulness in reconstructing the phylogenetic relationships of the Loranthaceae. Inference of each gene tree was carried out in RaxML v.8.2.12 (Stamatakis 2014) using the GTR + I + G4 model, with branch support assessed using 1000 fast bootstraps.

### Gene loss and pseudogenization

On the obtained phylogenetic tree, we mapped gene loss and pseudogenization events detected in each species to trace the evolutionary trajectory of plastome degradation associated with the hemiparasitic lifestyle in Loranthaceae. To reconstruct the evolutionary pathway of Loranthaceae plastome degradation under a phylogenetic framework, we mapped gene loss and pseudogenization events detected in each species using the heatmap2 function in ‘ggplot2’ package in R (Wickham 2016). Also, we reconstructed the ancestral states for 11 PCGs, which were identified as pseudogenized or lost for two or more species using the ‘phytools’ package of R (R Core Team 2023), with equal-rates (ER) likelihood model and the ‘ace’ function (Revell 2012). We classified each PCG into three different categories: intact, pseudogene, and loss.

## Results

### Chloroplast genome content and structural comparison

**Figure 1.**
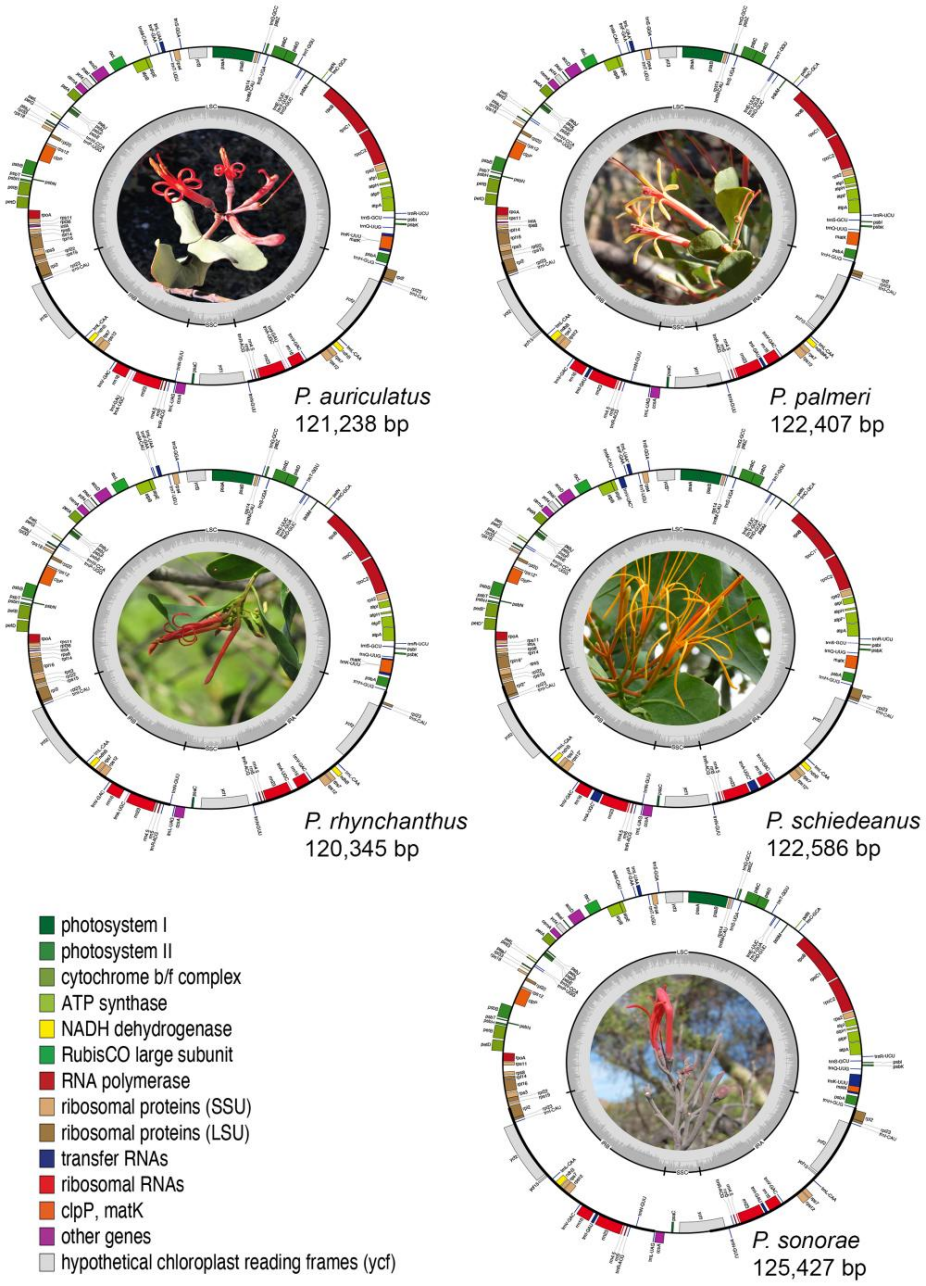
Circular representation of annotated plastid genomes (plastomes) of the five species of *Psittacanthus* included in this study. Structural organization of the gene content ring was colour-coded based on its functional category. Only functional genes are drawn. Different categories of genes labelled with distinct colours. Genes drawn inside the circle are transcribed clockwise, and those outside are transcribed counter clockwise. The dark grey in the inner circle corresponds to the GC content across the genome, and the light grey corresponds to the AT content.

The read depth of newly assembled *Psittacanthus* plastomes ranged from 1803.11× in *P*. *palmeri* to 7449.58× in *P*. *rhynchanthus*. The plastomes of the five *Psittacanthus* hemiparasitic species exhibited a typical quadripartite structure consisting of a pair of IR regions separated by a LSC and a SSC (Fig. 1). The lengths of these plastomes varied from 120 345 bp (*P*. *rhynchanthus*) to 125 427 bp (*P*. *sonorae*), exhibiting a genomic size difference of 5082 bp. The *P*. *sonorae* plastome showed the largest LSC region (73 497 bp) and *P*. *palmeri* the shortest LSC region (71 489 bp), whereas the *P*. *rhynchanthus* plastome showed the largest SSC region (8672 bp) and *P*. *palmeri* the shortest SSC region (6080 bp). The smallest IR region was observed in the *P*. *rhynchanthus* plastome (19 793 bp), whereas the largest IR region was observed in the plastome of *P*. *sonorae* (22 903 bp). The GC content was similar among all species, ranging from 36.4% to 36.9% (Table 1).

**Figure 2.**
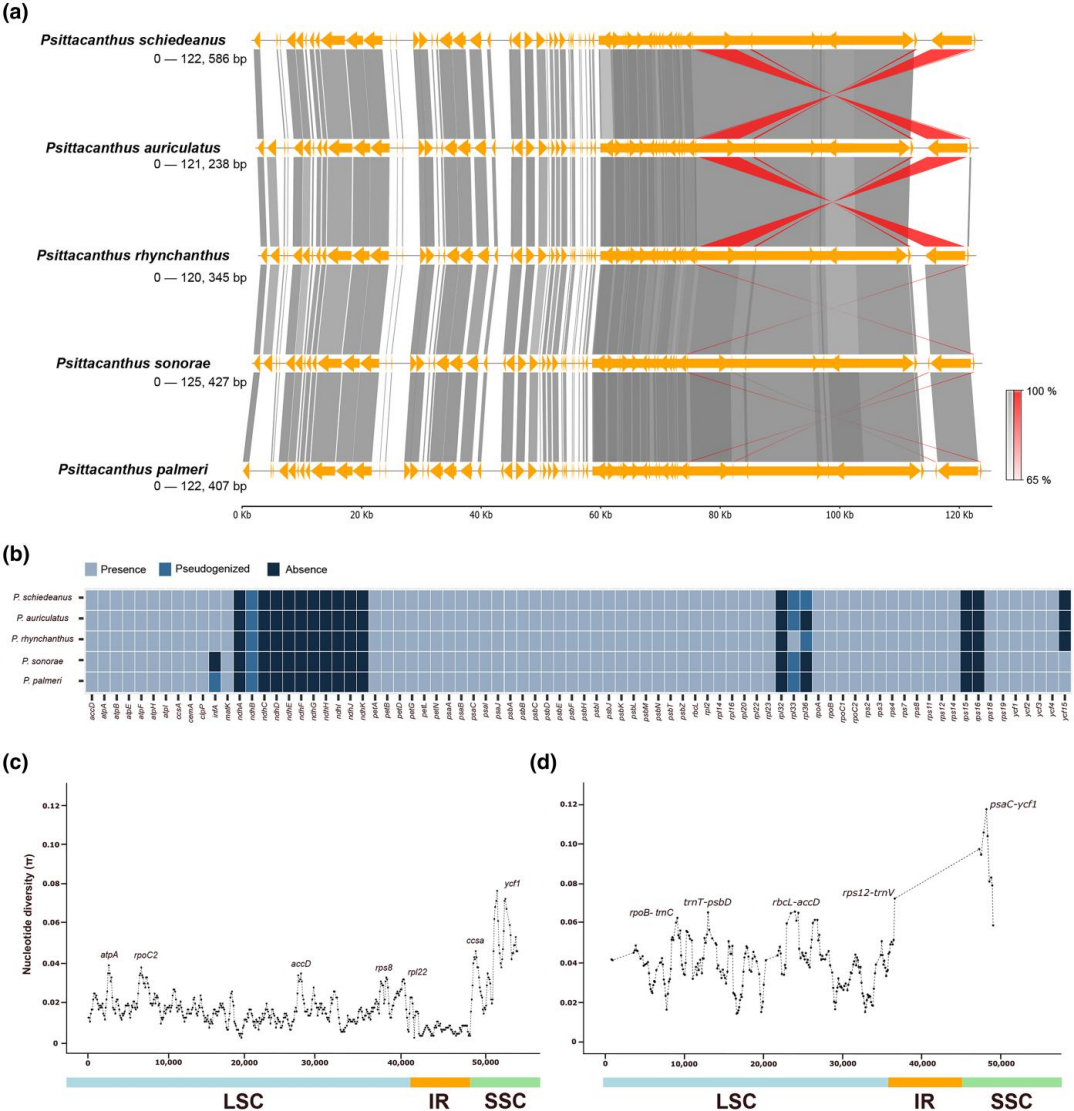
Structure and comparison of genes in *Psittacanthus* plastomes. (a) Synteny plot of plastomes of the five species of *Psittacanthus*. The synteny plot shows the conservation of gene order among genomes. Normal links shown with grey colour, inverted links with red colour, and gene features with yellow colour. (b) Heatmap showing the distribution of the presence of, absence of, and pseudogenized genes for 60 PCGs from plastomes of the five species of *Psittacanthus* included in this study. (c) Sliding window analysis of nucleotide variability for coding regions among the plastomes of the five *Psittacanthus* species included in this study (window length = 1000 bp; step size = 500 bp). The *x*-axis and *y*-axis indicate the position of the midpoint of the nucleotide diversity of each window. (d) Sliding window analysis of nucleotide variability for noncoding regions + introns among the plastomes of the five *Psittacanthus* species included in this study (window length = 1000 bp; step size = 500 bp). The *x*-axis and *y*-axis indicate the position of the midpoint of the nucleotide diversity of each window. The most variable regions are labelled.

Gene order is highly conserved in all five *Psittacanthus* plastomes, whereas the total gene number, as well as protein-coding rRNA and tRNA genes, is slightly different (Table 1). The visualization of syntenic relationships between plastomes showed a high level of sequence similarity and structural conservation across species (Fig. 2a). The synteny visualization demonstrated that the more closely related species *P. schiedeanus* and *P*. *auriculatus* exhibited higher levels of synteny and similarity corresponding to the inverted region of the *ycf2* gene in both species (Fig. 2a).

The loss of most of the NAD(P)H-dehydrogenase (*ndh*) complex (10 out of 11 genes), *rpl32*, *rps15*, and *rps16* genes, was identified in all studied *Psittacanthus* species (Fig. 2b). In addition, the *infA* and *ycf15* genes were not identified in some of the species, the former in *P*. *sonorae* and the latter in *P. rhynchanthus*, *P*. *auriculatus*, and *P*. *schiedeanus* (Fig. 2b). Pseudogenization for the *ndhB* gene was identified in all studied *Psittacanthus* species and *infA* in *P*. *palmeri* (Fig. 2b). The *rpl36* gene was lost in *P. sonorae*, *P. palmeri*, and *P. schiedeanus* and pseudogenized in *P*. *auriculatus* and *P. rhynchanthus*, whereas *rpl33* was identified as a pseudogene in *P. sonorae*, *P. palmeri*, *P. auriculatus*, and *P. schiedeanus*, i.e. identified as a potentially functional gene in the *P. rhynchanthus* plastome (Fig. 2b). Lastly, the loss of *trnV-UAC* occurred in all *Psittacanthus* species except *P. schiedeanus*, *trnA-UGC* was lost in *P. sonorae* and *P. palmeri* and pseudogenized in *P. rhynchanthus* and *P. auriculatus*, and *trnK-UUU* was only lost in *P*. *palmeri*.

For the studied *Psittacanthus* species, nucleotide diversity (*π*) for the protein-coding regions averaged 0.017, ranging from 0.002 (*psaB* and *rpl2* genes) to 0.075 (*ycf1* gene) (Table 2). Among the PCGs analysed, five genes (*atpA*, *rpoC2*, *accD*, *rps8*, and *rpl22*) exhibited *π* values > 0.03, whereas two genes (*ccsa* and *ycf1*) had *π* values > 0.04 (Fig. 2c). Compared with the LSC and SSC regions, the nucleotide diversity in the IR region showed the smallest *π* values. The nucleotide diversity values in noncoding regions were higher compared with those in the coding regions, with values ranging from 0.014 to 0.11; the regions with the highest diversity were *rpoB*-*trnC*, *trnT*-*psbD*, *rbcL*-*accD*, *rps12*-*trnV*, and *psaC*-*ycf1* (Fig. 2c).

**Table 2.** Summary of the characteristics of the most phylogenetically informative genes.

Gene	Aligned length (bp)	Polymorphic sites (S)	Missing data (%)	Nucleotide diversity (*π*)	Max value reached at
*ccsA*	1002	358	5.22	0.087	0.44
*matK*	1722	131	14.09	0.080	0.67
*rpoB*	3252	916	0.98	0.042	0.99
*rpoC2*	4429	275	8.81	0.080	0.81
*ycf1*	6480	42	25.64	0.127	0.40
*ycf2*	7308	968	7.87	0.019	0.99

### Contraction and expansion of inverted repeats

**Figure 3.**
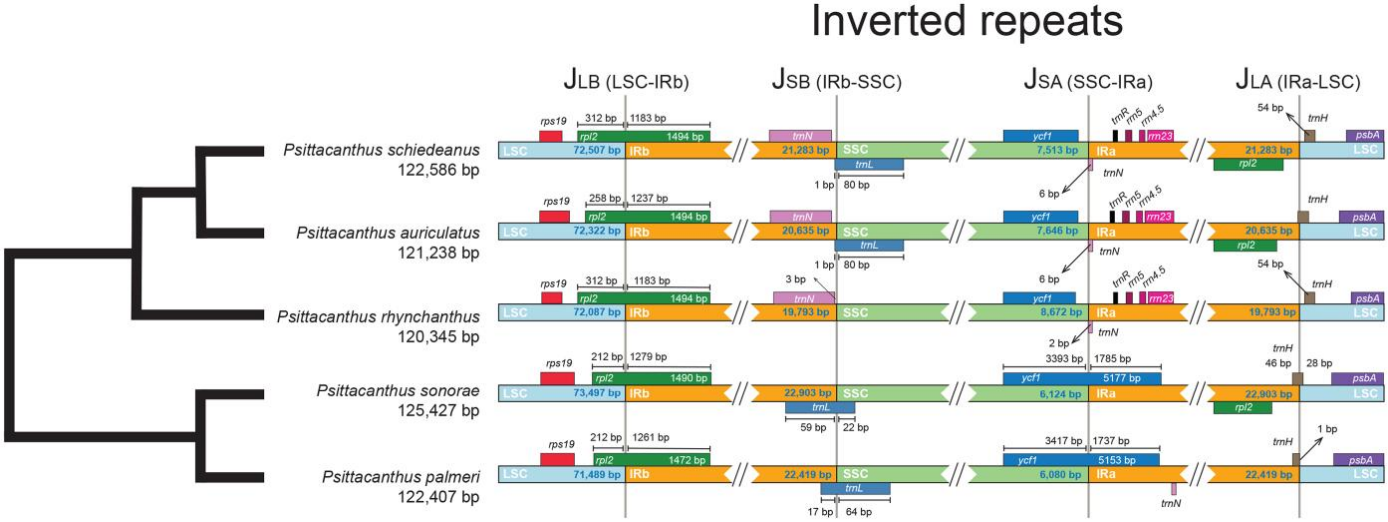
A phylogenetic tree of *Psittacanthus* along with distances between adjacent genes and junctions of the SSC, LSC, and two IR regions among plastomes of the five species of *Psittacanthus* included in this study. Boxes above and below the main line indicate the adjacent border genes. The figure is not to scale regarding sequence length and only shows relative changes at or near the IR/SC junctions.

The results indicated expansions/contractions throughout, with slight variations in the junctions of the IR and SSC regions and between the IR and LSC regions in the *Psittacanthus* plastomes (Fig. 3). The *ycf1* gene was located completely within the SSC region in *P. auriculatus*, *P. rhynchanthus*, and *P*. *schiedeanus*, but in *P*. *palmeri* and *P*. *sonorae* the same gene passes through the SSC and IRa regions. The junctions between the IRb and SSC regions were located between the *trnN* and *trnL* genes in *P. auriculatus* and *P*. *schiedeanus*, but not in *P*. *rhynchanthus*. For closely related *P*. *palmeri* and *P*. *sonorae*, the *trnL* gene passes through the IRb and SSC junctions. The IRa–LSC junction was characterized by the presence of the *psbA* and *trnH* genes in all species, whereas the *rpl2* gene was located through the LSC–IRb junctions in all plastomes (Fig. 3).

### Phylogenetic analysis

**Figure 4.**
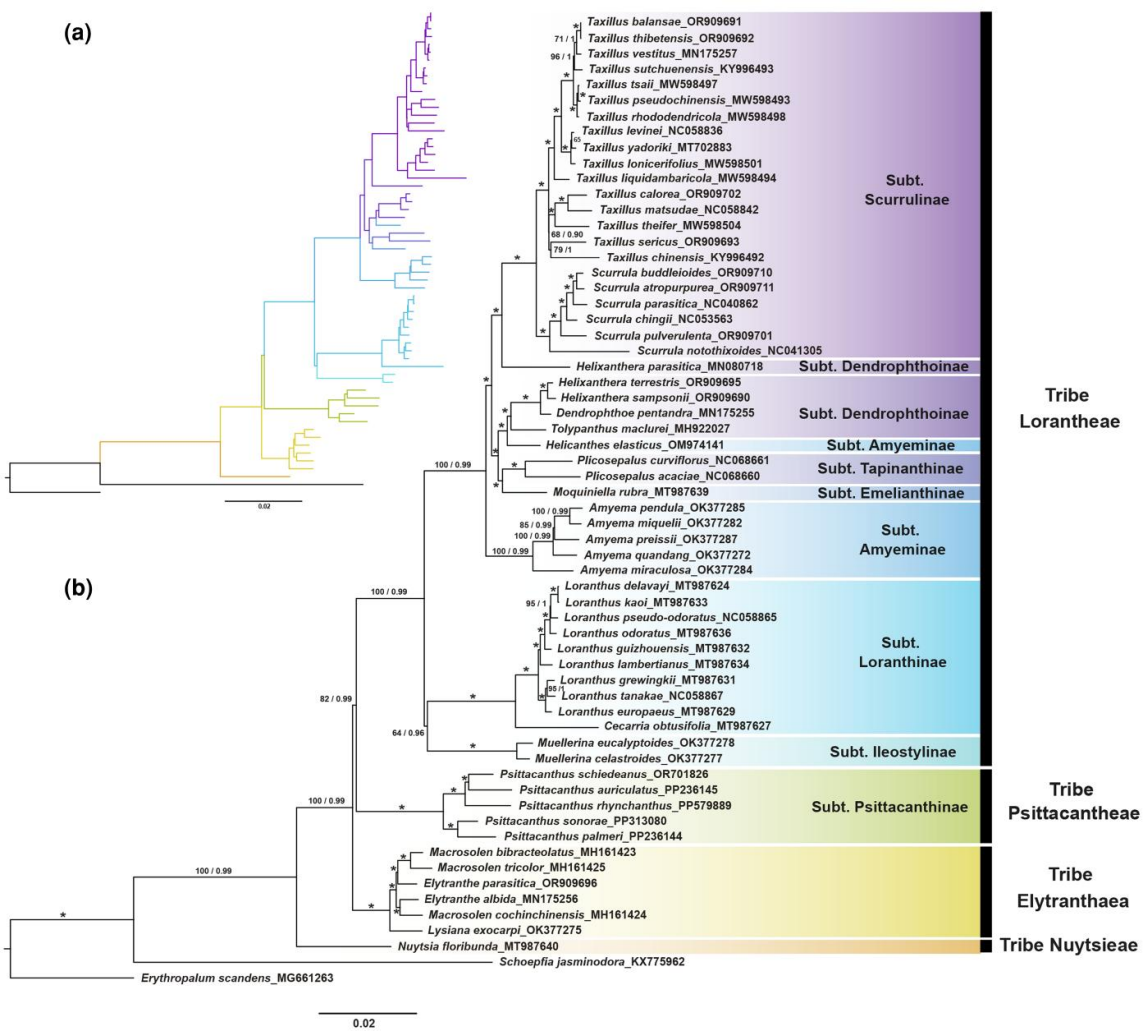
Phylogenetic tree of Loranthaceae reconstructed by analysing 60 plastid PCGs and plastomes of 52 species of Loranthaceae using (a) Bayesian inference and (b) ML methods. Numbers above branches indicate bootstrap (BS) percentage/PP, with the asterisk (*) indicating full support in both analyses (BS = 100%, PP = 1.00) from 1000 fast replicates. Label annotation against each species name represents the GenBank accession number.

The final alignment of 60 PCGs shared by 62 taxa was 59 703 bp with 6538 parsimony informative sites and 7.3% of missing data. ML and Bayesian inference BI analyses generated identical tree topologies (Fig. 4), with most nodes being fully supported (BS = 100%, PP = 1.00). Our results showed that *N. floribunda* was strongly supported as the sister group to the remaining Loranthaceae lineages (BS = 100%, PP = 1.00/0.99; Fig. 4). Within the tribe Elytrantheae, *Lysiana exocarpi* was placed sister to a well-supported clade that included *Elytranthe* and *Macrosolen* with high support values (BS = 100%, PP = 1.00). However, the position of *Lysiana* as sister to the rest of the Elytrantheae remains uncertain, due to the absence of several genera and species in Elytrantheae in this study. *Elytranthe albida* and *Elytranthe parasitica* were nested within *Macrosolen* with high support value (BS = 100%, PP = 1.00). For the largest tribe Lorantheae retrieved with strong support value (BS = 100%, PP = 1.00), subtribes Ileostylinae and Loranthinae were placed sister to clade composed of subtribes Amyeminae, Emelianthinae, Tapinanthinae, Dendrophthoinae, and Scurrulinae with strong support value (BS = 100%, PP = 0.99) and subtribes Amyeminae and Dendrophthoinae as paraphyletic. Lastly, the five species of *Psittacanthus* (tribe Psittacantheae, subtribe Psittacanthinae) were recovered as one clade with strong support (BS = 100%, PP = 1.00) and placed sister to tribe Lorantheae with moderate support value (BS = 82%, PP = 0.99), in which *P*. *sonorae* and *P*. *palmeri* formed a subclade that was placed sister to the *P*. *rhynchanthus* and *P*. *auriculatus* + *P*. *schiedeanus* subclade (Fig. 4).

### Loranthaceae informativeness profiles

Profiles of PI for each of the 60 PCGs showed *ycf1* as the most informative locus, increasing quickly in shallow time spans and attaining the highest values at a reference time (phylogenetic depth) of 0.4 (Supplementary Fig. S2a and b). Other genes with considerable PI values were *rpoC2*, *matK*, *ycf2*, *rpoB*, and *ccsA* (Table 2, Supplementary Fig. S2c). PCGs with high net PI values had long lengths (>1000 bp), although other long-length genes (e.g. *atpA*, *atpB*, *psbA*, *psbB*, *psbC*, *psbD*, *rbcL*, and *rpoA*) showed low PI values. However, individual gene trees recovered from the most informative genes showed topological discrepancies in the position of the Psittacantheae and Elytrantheae tribes as well as the subtribe Ileostylinae phylogenetic position (Supplementary Fig. S3). Likewise, the phylogenetic trees based on *ccsA* or *ycf2* showed low support for shallow nodes, whereas the tree based on *ycf1* was the only one that showed similar backbone topologies to the tree recovered with the 60 PCGs, with high support values for shallow nodes, although at deeper nodes the support values were lower. Finally, the gene tree based on *rpoC2* recovered the worst tree in terms of topology and support values (Supplementary Fig. S3).

### Gene loss and pseudogenization

**Figure 5.**
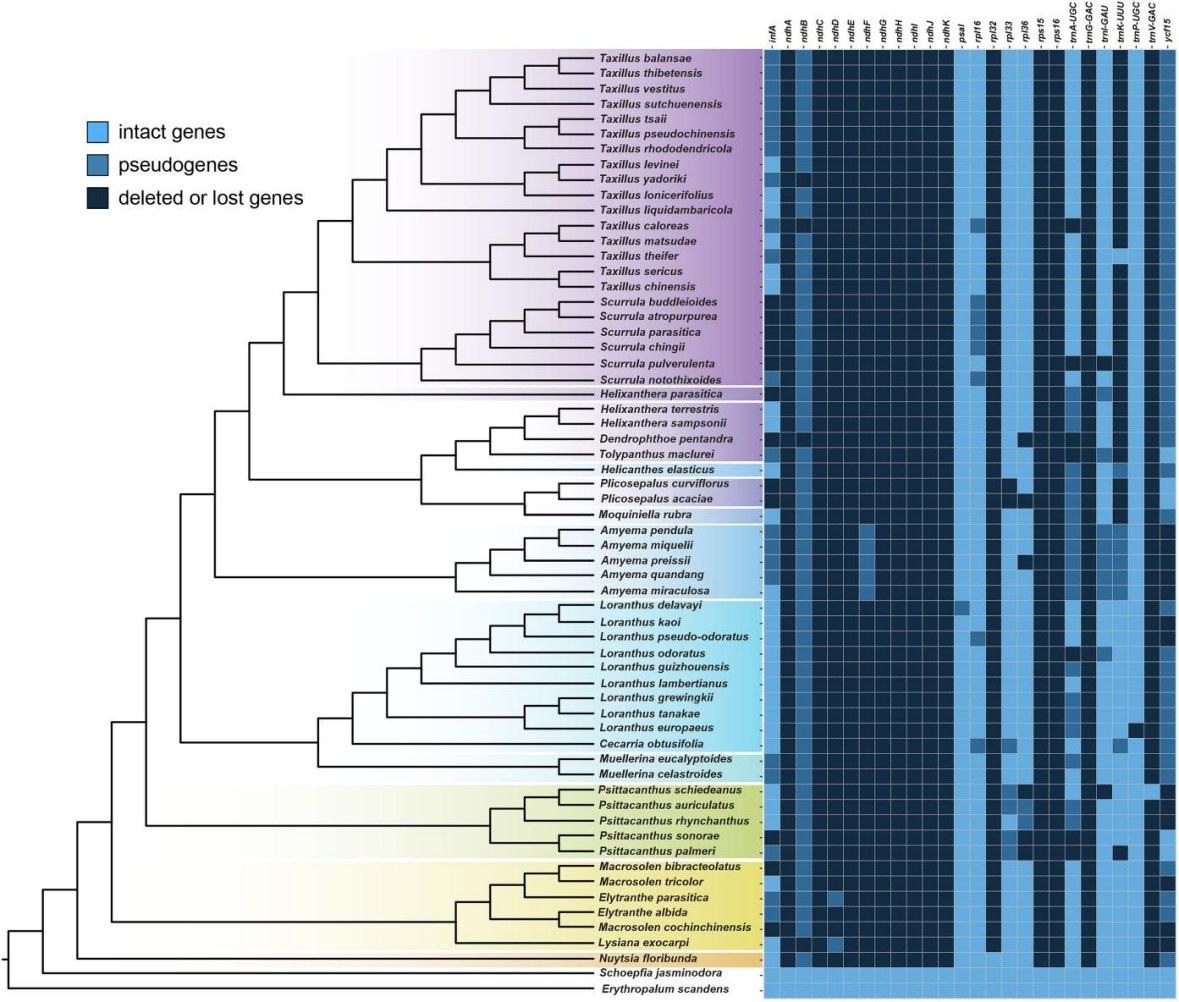
Pseudogenization or loss of PCGs heatmap in the plastome of 60 species of Loranthaceae and the autotrophic nonparasitic woody climber *E. scandens* (Erythropalaceae) and the woody root parasite *S. jasminodora* (Schoepfiaceae) outgroup species (Supplementary Table S2).

Gene loss and pseudogenization events were mapped onto a phylogenetic tree (Fig. 5). Eight *ndh* genes were absent in Loranthaceae, except *ndhB* in 56 species, *ndhD* in *E. parasitica* and *L. exocarpi* (tribe Elytrantheae), and *ndhF* in the five *Amyema* species (subtribe Amyeminae) (Fig. 5). Pseudogenization events were detected in 12 genes for Loranthaceae (Fig. 5). Additionally, unique pseudogenized or gene loss events were identified for *psaI* in *Loranthus delavayi* and *trnP*-*UGC* in *Loranthus europaeus*. The *rps15* and *rps16* genes, as well as the *trnG-GAC* gene, were also absent in all Loranthaceae chloroplast genomes (Fig. 5).

**Figure 6.**
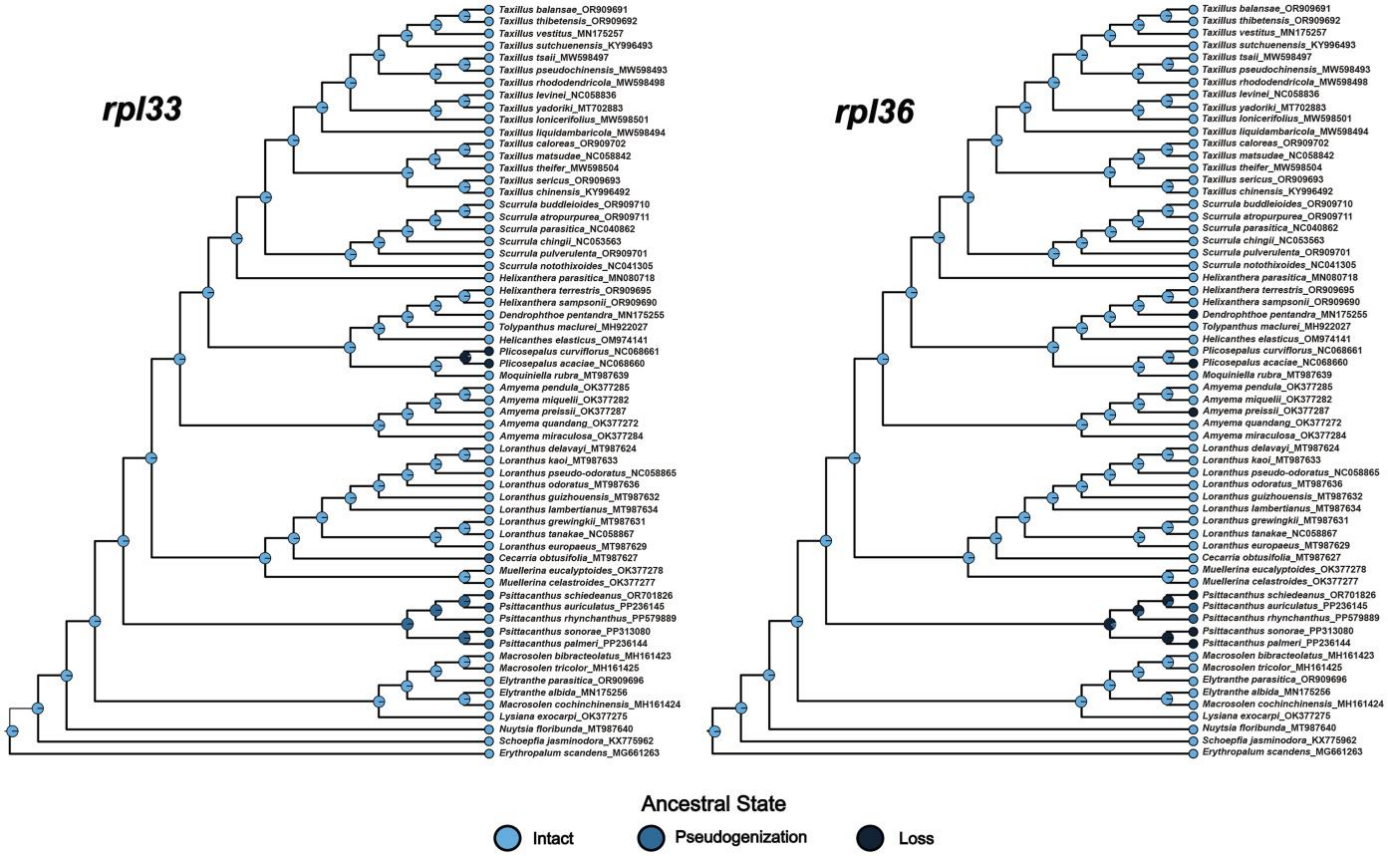
Ancestral state reconstruction of pseudogenization and loss of *rpl33* and *rpl36* genes in Loranthaceae using the ER likelihood model. The plastid genes were classified into three types (intact, pseudogenization, and loss). The pies indicate the ancestral state reconstruction. Light blue indicates a functional gene; dark blue indicates the gene becomes pseudogenized; and black indicates gene loss.

The ancestral reconstruction using the ER likelihood model revealed that *infA* and *trnA* genes displayed losses and pseudogenization differently within Loranthaceae. In contrast, loss and pseudogenization of the *ycf15* gene occurred in all analysed species except for *Plicosepalus acaciae*, *Plicosepalus curviflorus*, *Tolypanthus maclurei*, *P. palmeri*, and *P*. *sonorae* (Supplementary Fig. S1). The pseudogenization of *rpl16* and *trnI* might have occurred in the stem lineage ancestor of *Scurrula* and *Amyema*, and the pseudogenization of *rpl33* in *Psittacanthus* also occurred in *Cecarria obtusifolia*, but the further loss was observed in *P. acaciae* and *P*. *curviflorus*. Lastly, the pseudogenization or loss of the *rpl36* gene was observed in *Psittacanthus*, but the loss was observed in *Amyema preissii*, *P. acaciae*, and *Dendrophthoe pentandra* (Fig. 6).

## Discussion

In recent years, several studies have contributed to understanding the chloroplast genome evolution in hemiparasites of Santalales. However, these studies are focused almost exclusively on Asian (Li et al. 2017, Shin and Lee 2018, Liu et al. 2019, 2021, Nie et al. 2019, Chen et al. 2020, Al-Juhani et al. 2022, Darshetkar et al. 2023, Tang et al. 2024) and European lineages (Guo et al. 2020, 2021, Nickrent et al. 2021, Su et al. 2021, Tang et al. 2024), while mistletoes in the American Continent have received no attention. In our study, we sequenced, assembled, and annotated the chloroplast genomes of five *Psittacanthus* species (Psittacantheae tribe), generating valuable genomic data to enhance our understanding of genome evolution in parasitic plants.

### Size and structural variation in Psittacanthus chloroplast genomes

Typically, chloroplast genomes of autotrophic angiosperms with sizes ranging from 115 to 165 kb in size show highly conserved gene composition (101–118 unique genes), as well as collinear sequences arrangement (Bock 2007, Qu et al. 2023). The *Psittacanthus* chloroplast genomes reported here show a reduction in size and gene content between 19.7%–22.9% and 13.4%–14.3%, respectively, relative to the chloroplast genome of the outgroup *E. scandens* (Erythropalaceae), a nonparasitic species placed near the base in the large Santalales order (Zhu et al. 2018). Moreover, the chloroplast genomes of *Psittacanthus* are significantly larger than the one of *S. jasminodora* (118 743 bp), a root hemiparasite in Schoepfiaceae, a more closely related family to Loranthaceae, and the *Psittacanthus* chloroplast genomes encode fewer genes (96–97 unique genes) compared with 112 found in *S*. *jasminodora* (Su and Hu 2016). Within the Loranthaceae family, plastome sizes exhibit significant variation. Genera such as *Loranthus*, *Scurrula*, *Plicosepalus*, *Taxillus*, and *Tolypanthus* possess chloroplast genomes of similar size to those reported here for *Psittacanthus*, whereas *Cecarria*, *Dendrophthoe*, *Phyllodesmis*, and *Scurrula pulverulenta* have smaller plastomes. In contrast, basal genera such as *Elytranthe*, *Macrosolen*, and *Nuytsia* have notably larger plastomes sizes, ranging from 126 621 to 139 027 bp (Li et al. 2017, Shin HW, Lee NS 2018, Al-Juhani et al. 2022, Tang et al. 2024). These results reflect distinct evolutionary trajectories in genome size and gene content among these hemiparasitic plants.

The reduction of plastome size is common phenomenon observed in parasitic plants (Petersen et al. 2015, Chen et al. 2020, Guo et al. 2020, Darshetkar et al. 2023). While hemiparasites are expected to maintain most or all their photosynthetic genes (Frailey et al. 2018), this process is highly variable across different lineages and appears to have occurred independently in multiple clades (Guo et al. 2020, Su et al. 2021). Pseudogenization/gene loss has been linked to size variation in the chloroplast genome of parasitic plants (Bungard 2004, Xiao-Ming et al. 2017, Guo et al. 2020, Liu et al. 2021, Su et al. 2021, Darshetkar et al. 2023). Even though these mechanisms might explain the size reduction observed in the *Psittacanthus* chloroplast genomes, when compared with *E. scandens*, the observed size variation among *Psittacanthus* species chloroplast genomes could also be explained by additional processes such as variations in the length of intergenic regions and the expansion/contraction at the borders of IRs (Petersen et al. 2015, Xiao-Ming et al. 2017). Intergenic region variation has been reported as a mechanism that mainly affects the variation in chloroplast genomes size at the intrageneric level (Xiao-Ming et al. 2017). In this context, our sequence divergence analysis revealed that the highest proportion of the intergenic regions occurred in the largest chloroplast genome (*P*. *sonorae*) with 40.6% of the total size of the plastome, whereas the lowest proportion of intergenic regions (37%) occurred in *P*. *rhynchanthus*, the smallest plastome reported for *Psittacanthus*. Moreover, previous studies have related a greater decrease in intergenic regions with an increasing degree of parasitism and with improved energy efficiency and nutrition utilization (McNeal et al. 2007, Wu et al. 2009, Petersen et al. 2015), as well as reinforced fitness to the partial heterotrophic lifestyle (Guo et al. 2020).

### Inverted repeats and rearrangement patterns

The expansion/contraction at the borders of IRs are major factors contributing to plastome size variation (e.g. Yao et al. 2015, Wang et al. 2017). We found that IR boundary shifts are accompanied by variation in plastome size in *Psittacanthus*. More closely related species in *Psittacanthus* showed similar IR boundary shifts where the *P*. *auriculatus*/*P*. *schiedeanus*/*P*. *rhynchanthus* clade showed the *ycf1* gene located exclusively within the SSC region. In contrast, for the *P*. *sonorae*/*P*. *palmeri* clade, the *ycf1* gene was located crossing both the SSC and IRa regions, a trait shared with nonparasitic *E*. *scandens* (Zhu et al. 2018), suggesting that a reduction of the IR regions is occurring in the *Psittacanthus* chloroplast genomes. The IR shifts are common in hemiparasitic Santalales and have been associated with the functional loss of *ndh* genes (Shin and Lee 2018, Guo et al. 2020, Edlund et al. 2025). However, rearrangements in plastid genomes are not exclusive to parasitic plants, so they might not be connected necessarily to a photosynthesis-driven conservative evolution (Krause 2011).

We observed a high level of collinearity among the chloroplast genomes of the species of *Psittacanthus* included in our study. This observation suggests that no significant structural changes in the plastomes have occurred throughout their evolutionary history, as reported for chloroplast genomes of phylogenetically distant genera from Loranthaceae (Su et al. 2021, Darshetkar et al. 2023, Tang et al. 2024) and for other Santalales (Liu et al. 2021, Edlund et al. 2025). However, the lack of major structural rearrangements contrasts with major structural rearrangements reported in other orders of hemiparasitic plants (Zhang et al. 2022, Chen et al. 2024). Unlike other hemiparasitic plant orders (e.g. Lamiales), which plastomes exhibit major structural rearrangements with ≤36% GC content (Wicke et al. 2013, 2016, Zhang et al. 2020a), hemiparasites in Santalales show remarkably high plastome synteny. Nevertheless, cross-order comparisons between parasitic lineages (e.g. Santalales vs. Lamiales) require caution, as observed differences may arise from lineage-specific evolutionary dynamics rather than parasitic adaptations alone. Altogether, these results reveal that the chloroplast genomes for these *Psittacanthus* species are highly conserved structurally and highly similar in terms of gene content compared with earlier Loranthaceae lineages.

### Phylogenetic relationships and phylogenetic informativeness

The tree topologies from ML and BI phylogenetic analyses of Loranthaceae based on chloroplast genomes of 60 species including five *Psittacanthus* species of the previously unsampled tribe Psittacantheae were largely congruent with previous studies (Vidal-Russell and Nickrent 2008, Liu et al. 2018, Nickrent et al. 2010, Su et al. 2021, Tang et al. 2024). The sister relationships between the facultative root parasite *N. floribunda* (Nuytsieae tribe) and the rest of the obligate stem parasites Loranthaceae tribes and between the tribe Elytrantheae and the other Loranthaceae tribes were recovered with strong support. However, the genera *Macrosolen* and *Elytranthe* in the tribe Elytrantheae and *Helixanthera* in the tribe Lorantheae are recovered as paraphyletic (see also Vidal-Russell and Nickrent 2008, Liu et al. 2018, Tang et al. 2024). *Helixanthera parasitica* was placed sister to the subtribe Scurrulinae clade (*Scurrula* and *Taxillus*), which is consistent with previous findings (Liu et al. 2018, Su et al. 2021, Tang et al. 2024 ). Within the tribe Elytrantheae, *L. exocarpi* was recovered as sister to a well-supported clade that included both *Elytranthe* and *Macrosolen* species, which is consistent with previous studies that have proposed these morphologically similar genera to be treated taxonomically as congeners (Barlow 1997, Liu et al. 2018, Tang et al. 2024). For the largest tribe Lorantheae, subtribes Ileostylinae and Loranthinae were placed sister to clade comprising Amyeminae, Emelianthinae, Tapinanthinae, Dendrophthoinae, and Scurrulinae with strong support value, though Amyeminae and Dendrophthoinae were retrieved as paraphyletic (see also Tang et al. 2024). In addition, our study placed the clade with plastomes of five *Psittacanthus* species, previously unsampled members of the tribe Psittacantheae, sister to members of the tribe Lorantheae (see also Vidal-Russell and Nickrent 2008). Despite the robust sampling at the generic level in previous phylogenetic studies of Loranthaceae (e.g. Vidal-Russell and Nickrent 2008, Grímsson et al. 2017, Liu et al. 2018, Ortiz-Rodriguez et al. 2018), the limited number of chloroplast markers employed produced low node support values along the backbone of the reported trees, polytomies, and topological incongruences in some genera. Studies using chloroplast genome sequence data have shown well-resolved clades, but increased taxonomic sampling is still needed to evaluate the monophyly of most subtribes in Loranthaceae (Guo et al. 2020, Nickrent et al. 2021, Su et al. 2021, Tang et al. 2024).

Whereas previous studies have not assessed the utility of individual plastome genes for phylogenetic inferences in Loranthaceae, our analysis identified the *ycf1* gene as particularly useful. Among the most informative PCGs, *ycf1* produced tree topologies comparable to those from full plastome analysis (60 genes), despite its high mutation rate and nucleotide diversity (consistent with Su et al. 2021). Although too long for standard PCR, *ycf1* shows promise for resolving shallow-level relationships. In contrast, the IR-located *ycf2* gene showed low nucleotide diversity but strong support for deep nodes. Combining these two markers may help resolve phylogenetic relationships at both shallow and deep levels in Loranthaceae.

### The loss of genes in Loranthaceae

Based on the obtained phylogenetic tree (see Fig. 5), our results show that the loss/pseudogenization of the tRNA gene (*trnG-GAC*), two ribosomal protein genes (*rps15* and *rps16*), and 10 *ndh* genes (*ndhA*, *ndhC–K*) are plesiomorphic traits in Loranthaceae. The loss of *ndh* genes is common in hemiparasitic Santalales, including Loranthaceae, except for the *ndhB* gene (Guo et al. 2020, Nickrent et al. 2021, Su et al. 2021, Darshetkar et al. 2023, Edlund et al. 2025), which in most species including *Psittacanthus* has been pseudogenized. Two explanations for the loss of *ndh* genes have been proposed: (i) the function of the *ndh* gene is related to hydric stress conditions (Horváth et al. 2000, Martín and Sabater 2010, Chen et al. 2020) and (ii) its role in photosynthesis is often dispensable and even selected against in some plant lineages (Lin et al. 2017, Frailey et al. 2018). Edlund et al. (2025) found one functional loss of the 11 *ndh* genes in Santalales and signs of relaxed selection in autotroph lineages, suggesting that PGR5/PGRL1 protein complex might act as an alternative pathway for photosystem I cyclic electron transport. Although the pseudogenization and eventual loss of the *ndh* genes have been proposed as early genomic changes associated with the transition to a parasitic lifestyle (Shin and Lee 2018, Darshetkar et al. 2023), establishing a direct link between *ndh* degradation and hemiparasitism remains challenging. First, there is insufficient physiological evidence demonstrating that the loss of these genes impairs function in hemiparasitic species. Second, no studies have investigated whether *ndh* genes have been functionally transferred to the nuclear or mitochondrial genome. Moreover, the absence of *ndh* genes is not exclusive to parasitic plants, multiple nonparasitic lineages, including gymnosperms (Pinaceae; Wakasugi et al. 1994; Gnetales; Braukmann et al. 2009) and angiosperms such as orchids (Jheng et al. 2012, Lin et al. 2017) and cacti (Sanderson et al. 2015), as well as species of Alismatales (Iles et al. 2013, Peredo et al. 2013), Geraniales (Blazier et al. 2011), and Ranunculales (Sun et al. 2017), seem not to suffer negative impacts. Thus, the *ndh* complex might be of limited biological significance for contemporary plants (Ruhlman et al. 2015, Lin et al. 2017).

While the loss of the *rpl32* gene appears to have occurred during the evolutionary transition from root parasitism to stem parasitism, comprehensive testing of this hypothesis requires analysis of all the facultative root-parasitic lineages (*N. floribunda*, *Atkinsonia ligustrina*, and *Gaiadendron punctatum*). The loss/pseudogenization of plastid ribosomal protein-encoding genes has been detected not only in parasitic plants (McNeal et al. 2007, Su et al. 2018), but also in a wide range of autotrophic angiosperms (Sabir et al. 2014, Park et al. 2015, Zhang et al. 2020b, Silva et al. 2021). Thereby, the loss of *rpl32*, *rps15*, and *rps16* genes may also be related to the migration of genes to the nucleus and not necessarily to the evolution of parasitism (Park et al. 2018, Dobrogojski et al. 2020, Guo et al. 2020, Yang et al. 2021, Darshetkar et al. 2023). Interestingly, the lineage-specific loss/pseudogenization of ribosomal protein genes (*rpl16*, *rpl33*, and *rpl36*) may reflect evolutionary parallelism, as these independent events occurred in closely related taxa sharing a common ancestor with functional copies of these genes (see Figs 5 and 6; Tang et al. 2024). Particularly, the loss/pseudogenization of the *rpl33* gene has been reported in many Santalales (Chen et al. 2020) but is uncommon in Loranthaceae, suggesting that the degradation of the *rpl33* gene has occurred several times independently. Although the *rps15*, *rpl33*, and *rpl36* genes are not essential for chloroplast gene translation, the remaining genes play essential roles (Tiller and Bock 2014). Hence, the loss of these plastid ribosomal protein-encoding genes in Loranthaceae might be compensated for by other plastid *rpl*/*rps* genes or by nuclear-encoded *rpl*/*rps* genes.

Although pseudogenization of *psbZ*, *matK*, *rpoC2*, *ycf1*, and *ycf2* genes has been previously reported in Loranthaceae (Tang et al. 2024), our dual annotation approach (combining Geseq and PGA annotators) successfully identified these genes with enhanced confidence. This is consistent with previous reports in which transcriptional evidence was found for the *rpoC2*, *ycf1*, and *ycf2* genes from *P. schiedeanus* by RNAseq (Ibarra-Laclette et al. 2022). These results suggest that these genes have undergone size reduction processes but continued to conserve the start and stop codons, and they also do not show intermediate stop codons. The loss/pseudogenization of the *infA* and *ycf15* genes in other Loranthaceae species were also observed in *Psittacanthus*. Both genes are characterized by showing anomalous distribution patterns in angiosperms and have been related to nuclear transfer events (Millen et al. 2001, Shi et al. 2013). Our phylogenetic analysis of plastomes showed that both the *infA* and *ycf15* genes are lost/pseudogenized several times along the evolution of Loranthaceae, which suggests multiple independent transfers to the nucleus (Millen et al. 2001). Lastly, the loss/pseudogenization of the *trnA*-*UGC* and *trnV*-*UAC* genes in *Psittacanthus* (except *P. schiedeanus*) is also observed in other Loranthaceae species as well as other Santalales (Chen et al. 2020, Darshetkar et al. 2023). Both genes have been considered essential for cell viability, so mechanisms such as the tRNA import from the cytosol to the plastids were proposed to replace the essential of these tRNA losses (Alkatib et al. 2012, Dalla Costa et al. 2022).

## Conclusion

Chloroplast genomes are currently the turning point of comparative studies in hemiparasitic plants. It is now essential to generate genomic resources for diverse lineages and then contrast different hypotheses about diverse evolutionary trajectories of hemiparasitic plastome degradation. Our results show that the plastomes of analysed species possess traits uncommon within Loranthaceae, including the loss or pseudogenization of *rpl33* and *rpl36* genes. The pseudogenization of the *ndhB* gene occurred in the plastomes of the five species of *Psittacanthus* included in this study. Contrary to previous studies, our findings reveal that despite the high synteny observed in *Psittacanthus* plastomes, significant size variation exists among species. This variation can be attributed to processes such as variations in the length of intergenic regions and the expansion/contraction of IR borders, traits that have been comparatively understudied in earlier Loranthaceae works. The present study establishes an important precedent to understanding plastome evolution in the New World mistletoes. Although our study demonstrates the utility of plastid genes for phylogenetics of Loranthaceae, some conflictive clades remain unsolved. These discrepancies could potentially be resolved using genomic data across Loranthaceae in the American Continent, particularly from the nuclear genome.

## Supplementary Material

plaf032_Supplementary_Data

## Data Availability

The data underlying this article are available in its supplementary material. Fully annotated plastomes were deposited in the NCBI GenBank database under the accession numbers listed in Table 1 and Supplementary Table S2.

## References

[plaf032-B1] Al-Juhani W, Al-Thagafi NT, Al-Qthanin RN. Gene losses and plastome degradation in the hemiparasitic species *Plicosepalus acacia*e and *Plicosepalus curviflorus*: comparative analyses and phylogenetic relationships among Santalales members. Plants. 2022;11:1869. 10.3390/plants11141869 35890506 PMC9317152

[plaf032-B2] Alkatib S, Scharff LB, Rogalski M et al The contributions of wobbling and superwobbling to the reading of the genetic code. PLoS Genet 2012;8:e1003076. 10.1371/journal.pgen.1003076 23166520 PMC3499367

[plaf032-B3] Amiryousefi A, Hyvönen J, Poczai P. IRscope: an online program to visualize the junction sites of chloroplast genomes. Bioinformatics 2018;34:3030–1. 10.1093/bioinformatics/bty220 29659705

[plaf032-B4] Banerjee A, Stefanović S. A comparative study across the parasitic plants of *Cuscuta* subgenus *Grammica* (Convolvulaceae) reveals a possible loss of the plastid genome in its section Subulatae. Planta 2023;257:66. 10.1007/s00425-023-04099-y 36826697

[plaf032-B5] Barlow BA . Loranthaceae. In: Kalkman C, Stevens PF, Kirkup DW, de Wilde WJJO, Booteboom HP (eds.), Flora Malesiana Series 1. Leiden, The Netherlands: National Herbarium of the Neatherlands, 1997, 209–401.

[plaf032-B6] Blazier CJ, Guisinger MM, Jansen RK. Recent loss of plastid-encoded *ndh* genes within *Erodium* (Geraniaceae). Plant Mol Biol 2011;76:263–72. 10.1007/s11103-011-9753-5 21327834

[plaf032-B7] Bock R . Structure, function, and inheritance of plastid genomes. In: Bock R (ed.), Cell and Molecular Biology of Plastids. Topics in Current Genetics, Vol. 19. Berlin, Germany: Springer, 2007, 29–63.

[plaf032-B8] Bolger AM, Lohse M, Usadel B. Trimmomatic: a flexible trimmer for Illumina sequence data. Bioinformatics 2014;30:2114–20. 10.1093/bioinformatics/btu170 24695404 PMC4103590

[plaf032-B9] Braukmann TWA, Kuzmina M, Stefanović S. Loss of all plastid *ndh* genes in Gnetales and conifers: extent and evolutionary significance for the seed plant phylogeny. Curr Genet 2009;55:323–37. 10.1007/s00294-009-0249-7 19449185

[plaf032-B10] Bromham L, Cowman PF, Lanfear R. Parasitic plants have increased rates of molecular evolution across all three genomes. BMC Evol Biol 2013;13:126. 10.1186/1471-2148-13-126 23782527 PMC3694452

[plaf032-B11] Bungard RA . Photosynthetic evolution in parasitic plants: insight from the chloroplast genome. BioEssays 2004;26:235–47. 10.1002/bies.10405 14988925

[plaf032-B12] Carbonell-Caballero J, Alonso R, Ibañez V et al A phylogenetic analysis of 34 chloroplast genomes elucidates the relationships between wild and domestic species within the genus *Citrus*. Mol Biol Evol 2015;32:2015–35. 10.1093/molbev/msv082 25873589 PMC4833069

[plaf032-B13] Chan PP, Lowe TM. tRNAscan-SE: searching for tRNA genes in genomic sequences. Methods Mol Biol 2019;1962:1–14. 10.1007/978-1-4939-9173-0_1 31020551 PMC6768409

[plaf032-B14] Chen X, Fang D, Wu C et al Comparative plastome analysis of root-and stem-feeding parasites of Santalales untangle the footprints of feeding mode and lifestyle transitions. Genome Biol Evol 2020;12:3663–76. 10.1093/gbe/evz271 31845987 PMC6953812

[plaf032-B15] Chen L-Q, Li X, Yao X et al Variations and reduction of plastome are associated with the evolution of parasitism in Convolvulaceae. Plant Mol Biol 2024;114:40. 10.1007/s11103-024-01440-1 38622367

[plaf032-B16] Civáň P, Foster PG, Embley MT et al Analyses of charophyte chloroplast genomes help characterize the ancestral chloroplast genome of land plants. Genome Biol Evol 2014;6:897–911. 10.1093/gbe/evu061 24682153 PMC4007539

[plaf032-B17] Dalla Costa TP, Silva MC, de Santana Lopes A et al The plastome of *Melocactus glaucescens* Buining & Brederoo reveals unique evolutionary features and loss of essential tRNA genes. Planta 2022;255:57. 10.1007/s00425-022-03841-2 35113261

[plaf032-B18] Darriba D, Posada D, Kozlov AM et al ModelTest-NG: a new and scalable tool for the selection of DNA and protein evolutionary models. Mol Biol Evol 2020;37:291–4. 10.1093/molbev/msz189 31432070 PMC6984357

[plaf032-B19] Darshetkar AM, Pable AA, Nadaf AB et al Understanding parasitism in Loranthaceae: insights from plastome and mitogenome of *Helicanthes elastica*. Gene 2023;861:147238. 10.1016/j.gene.2023.147238 36736502

[plaf032-B20] Dettke GA, Caires CS. *Psittacanthus* (Loranthaceae) in Brazil: new occurrences, lectotypifications, new synonyms and an illustrated key. Rodriguésia 2021;72:e00602020. 10.1590/2175-7860202172138

[plaf032-B21] Dierckxsens N, Mardulyn P, Smits G. NOVOPlasty: de novo assembly of organelle genomes from whole genome data. Nucleic Acids Res 2017;45:e18. 10.1093/nar/gkw955 28204566 PMC5389512

[plaf032-B22] Dobrogojski J, Adamiec M, Luciński R. The chloroplast genome: a review. Acta Physiol Plant 2020;42:98. 10.1007/s11738-020-03089-x

[plaf032-B23] Edlund M, Anderson BM, Su HJ et al Plastome evolution in Santalales involves relaxed selection prior to loss of *ndh* genes and major boundary shifts of the inverted repeat. Ann Bot 2025;135:515–30. 10.1093/aob/mcae145 39213003 PMC11897430

[plaf032-B24] Frailey DC, Chaluvadi SR, Vaughn JN et al Gene loss and genome rearrangement in the plastids of five hemiparasites in the family Orobanchaceae. BMC Plant Biol 2018;18:30. 10.1186/s12870-018-1249-x 29409454 PMC5801802

[plaf032-B25] Greiner S, Lehwark P, Bock R. Organellar GenomeDRAW (OGDRAW) version 1.3.1: expanded toolkit for the graphical visualization of organellar genomes. Nucleic Acids Res 2019;47:W59–64. 10.1093/nar/gkz238 30949694 PMC6602502

[plaf032-B26] Grímsson F, Kapli P, Hofmann CC et al Eocene Loranthaceae pollen pushes back divergence ages for major splits in the family. PeerJ 2017;5:e3373. 10.7717/peerj.3373 28607837 PMC5466002

[plaf032-B27] Guo X, Liu C, Zhang G et al The complete plastomes of five hemiparasitic plants (*Osyris wightiana*, *Pyrularia edulis*, *Santalum album*, *Viscum liquidambaricolum*, and *V. ovalifolium*): comparative and evolutionary analyses within Santalales. Front Genet 2020;11:597. 10.3389/fgene.2020.00597 32612639 PMC7308561

[plaf032-B28] Guo X, Zhang G, Fan L et al Highly degenerate plastomes in two hemiparasitic dwarf mistletoes: *Arceuthobium chinense* and *A. pini* (Viscaceae). Planta 2021;253:125. 10.1007/s00425-021-03643-y 34028602

[plaf032-B29] Horváth EM, Peter SO, Joët T et al Targeted inactivation of the plastid *ndhB* gene in tobacco results in an enhanced sensitivity of photosynthesis to moderate stomatal closure. Plant Physiol 2000;123:1337–50. 10.1104/pp.123.4.1337 10938352 PMC59092

[plaf032-B30] Ibarra-Laclette E, Venancio-Rodríguez CA, Vásquez-Aguilar AA et al Transcriptional basis for haustorium formation and host establishment in hemiparasitic *Psittacanthus schiedeanus* mistletoes. Front Genet 2022;13:929490. 10.3389/fgene.2022.929490 35769994 PMC9235361

[plaf032-B31] Iles WJD, Smith SY, Graham SW. A well-supported phylogenetic framework for the monocot order Alismatales reveals multiple losses of the plastid NADH dehydrogenase complex and a strong long-branch effect. In: Wilkin P, Mayo SJ (eds.), Early Events in Monocot Evolution. Cambridge, UK: Cambridge University Press, 2013, 1–28.

[plaf032-B32] Jheng C-F, Chen T-C, Lin J-Y et al The comparative chloroplast genomic analysis of photosynthetic orchids and developing DNA markers to distinguish *Phalaenopsis* orchids. Plant Sci 2012;190:62–73. 10.1016/j.plantsci.2012.04.001 22608520

[plaf032-B33] Jiang Y, Zhu C, Wang S et al Identification of three cultivated varieties of *Scutellaria baicalensis* using the complete chloroplast genome as a super-barcode. Sci Rep 2023;13:5602. 10.1038/s41598-023-32493-9 37019975 PMC10075158

[plaf032-B34] Jin J-J, Yu W-B, Yang J-B et al GetOrganelle: a fast and versatile toolkit for accurate de novo assembly of organelle genomes. Genome Biol 2020;21:241. 10.1186/s13059-020-02154-5 32912315 PMC7488116

[plaf032-B35] Katoh K, Rozewicki J, Yamada KD. MAFFT online service: multiple sequence alignment, interactive sequence choice and visualization. Brief Bioinform 2019;20:1160–6. 10.1093/bib/bbx108 28968734 PMC6781576

[plaf032-B36] Kearse M, Moir R, Wilson A et al Geneious basic: an integrated and extendable desktop software platform for the organization and analysis of sequence data. Bioinformatics 2012;28:1647–9. 10.1093/bioinformatics/bts199 22543367 PMC3371832

[plaf032-B37] Krause K . Piecing together the puzzle of parasitic plant plastome evolution. Planta 2011;234:647–56. 10.1007/s00425-011-1494-9 21850456

[plaf032-B38] Kuijt J . Monograph of *Psittacanthus* (Loranthaceae). Syst Bot Monogr 2009;86:1–361. https://www.jstor.org/stable/25592351

[plaf032-B39] Li X, Yang J-B, Wang H et al Plastid NDH pseudogenization and gene loss in a recently derived lineage from the largest hemiparasitic plant genus *Pedicularis* (Orobanchaceae). Plant Cell Physiol 2021b;62:971–84. 10.1093/pcp/pcab074 34046678 PMC8504446

[plaf032-B40] Li M, Zhang Y, Li Y et al The complete chloroplast genome of *Scurrula chingii* (W.C. Cheng) H.S. Kiu (Loranthaceae), a hemiparasitic shrub. Mitochondrial DNA B Resour 2021a;6:282–4. 10.1080/23802359.2020.1863166 33553646 PMC7850402

[plaf032-B41] Li Y, Zhang L, Wang T et al The complete chloroplast genome sequences of three lilies: genome structure, comparative genomic and phylogenetic analyses. J Plant Res 2022;135:723–37. 10.1007/s10265-022-01417-5 36260182

[plaf032-B42] Li C, Zheng Y, Huang P. Molecular markers from the chloroplast genome of rose provide a complementary tool for variety discrimination and profiling. Sci Rep 2020;10:12188. 10.1038/s41598-020-68092-1 32699274 PMC7376030

[plaf032-B43] Li Y, Zhou J-G, Chen X-L et al Gene losses and partial deletion of small single-copy regions of the chloroplast genomes of two hemiparasitic *Taxillus* species. Sci Rep 2017;7:12834. 10.1038/s41598-017-13401-4 29026168 PMC5638910

[plaf032-B44] Lin C-S, Chen JJW, Chiu C-C et al Concomitant loss of NDH complex-related genes within chloroplast and nuclear genomes in some orchids. Plant J 2017;90:994–1006. 10.1111/tpj.13525 28258650

[plaf032-B45] Liu S-S, Hu Y-H, Maghuly F et al The complete chloroplast genome sequence annotation for *Malania oleifera*, a critically endangered and important bioresource tree. Conserv Genet Resour 2019;11:271–4. 10.1007/s12686-018-1005-4

[plaf032-B46] Liu B, Le CT, Barrett RL et al Historical biogeography of Loranthaceae (Santalales): diversification agrees with emergence of tropical forests and radiation of songbirds. Mol Phylogenet Evol 2018;124:199–212. 10.1016/j.ympev.2018.03.010 29550535

[plaf032-B47] Liu X, Xu D, Hong Z et al Comparative and phylogenetic analysis of the complete chloroplast genome of *Santalum* (Santalaceae). Forests 2021;12:1303. 10.3390/f12101303

[plaf032-B48] López-Giráldez F, Townsend JP. PhyDesign: an online application for profiling phylogenetic informativeness. BMC Evol Biol 2011;11:152. 10.1186/1471-2148-11-152 21627831 PMC3124428

[plaf032-B49] Martín M, Sabater B. Plastid *ndh* genes in plant evolution. Plant Physiol Biochem 2010;48:636–45. 10.1016/j.plaphy.2010.04.009 20493721

[plaf032-B50] McNeal JR, Kuehl JV, Boore JL et al Complete plastid genome sequences suggest strong selection for retention of photosynthetic genes in the parasitic plant genus *Cuscuta*. BMC Plant Biol 2007;7:57. 10.1186/1471-2229-7-57 17956636 PMC2216012

[plaf032-B51] Millen RS, Olmstead RG, Adams KL et al Many parallel losses of *infA* from chloroplast DNA during angiosperm evolution with multiple independent transfers to the nucleus. Plant Cell 2001;13:645–58. 10.1105/tpc.13.3.645 11251102 PMC135507

[plaf032-B52] Morales-Saldaña S, Villafán E, Vásquez-Aguilar AA et al The complete chloroplast genome sequence of *Psittacanthus schiedeanus* (Cham. & Schltdl.) G. Don (Santalales: Loranthaceae), the first plastome of a mistletoe species in the Psittacantheae tribe. Mitochondrial DNA B Resour 2024;9:5–10. 10.1080/23802359.2023.2298078 38187014 PMC10769147

[plaf032-B53] Nickrent DL . Parasitic angiosperms: how often and how many? Taxon 2020;69:5–27. 10.1002/tax.12195

[plaf032-B54] Nickrent DL, García MA. On the brink of holoparasitism: plastome evolution in dwarf mistletoes (*Arceuthobium*, Viscaceae). J Mol Evol 2009;68:603–15. 10.1007/s00239-009-9224-7 19479176

[plaf032-B55] Nickrent DL, Malécot V, Vidal-Russell R et al A revised classification of Santalales. Taxon 2010;59:538–58. 10.1002/tax.592019

[plaf032-B56] Nickrent DL, Su HJ, Lin RZ et al Examining the needle in the haystack: evolutionary relationships in the mistletoe genus *Loranthus* (Loranthaceae). Syst Bot 2021;46:403–15. 10.1600/036364421X16231785234748

[plaf032-B57] Nie L, Cui Y, Wu L et al Gene losses and variations in chloroplast genome of parasitic plant *Macrosolen* and phylogenetic relationships within Santalales. Int J Mol Sci 2019;20:5812. 10.3390/ijms20225812 31752332 PMC6888684

[plaf032-B58] Ohyama K, Fukuzawa H, Kohchi T et al Chloroplast gene organization deduced from complete sequence of liverwort *Marchantia polymorpha* chloroplast DNA. Nature 1986;322:572–4. 10.1038/322572a0

[plaf032-B59] Ornelas JF, Lara C, Morales-Saldaña S et al Insights into mistletoe seed germination: a study of hemiparasitic *Psittacanthus* Mart. (Santalales: Loranthaceae) mistletoes. Flora 2024;316:152527. 10.1016/j.flora.2024.152527

[plaf032-B60] Ortiz-Rodriguez AE, Guerrero EY, Ornelas JF. Phylogenetic position of Neotropical *Bursera*-specialist mistletoes: the evolution of deciduousness and succulent leaves in *Psittacanthus* (Loranthaceae). Bot Sci 2018;96:443–61. 10.17129/botsci.1961

[plaf032-B61] Paradis E, Claude J, Strimmer K. APE: analyses of phylogenetics and evolution in R language. Bioinformatics 2004;20:289–90. 10.1093/bioinformatics/btg412 14734327

[plaf032-B62] Park S, An B, Park S. Reconfiguration of the plastid genome in *Lamprocapnos spectabilis*: IR boundary shifting, inversion, and intraspecific variation. Sci Rep 2018;8:13568. 10.1038/s41598-018-31938-w 30206286 PMC6134119

[plaf032-B63] Park S, Jansen RK, Park S. Complete plastome sequence of *Thalictrum coreanum* (Ranunculaceae) and transfer of the *rpl32* gene to the nucleus in the ancestor of the subfamily Thalictroideae. BMC Plant Biol 2015;15:40. 10.1186/s12870-015-0432-6 25652741 PMC4329224

[plaf032-B64] Peredo EL, King UM, Les DH. The plastid genome of *Najas flexilis*: adaptation to submersed environments is accompanied by the complete loss of the NDH complex in an aquatic angiosperm. PLoS One 2013;8:e68591. 10.1371/journal.pone.0068591 23861923 PMC3701688

[plaf032-B65] Petersen G, Cuenca A, Seberg O. Plastome evolution in hemiparasitic mistletoes. Genome Biol Evol 2015;7:2520–32. 10.1093/gbe/evv165 26319577 PMC4607522

[plaf032-B66] Pond SL, Frost SD, Muse SV. HyPhy: hypothesis testing using phylogenies. Bioinformatics 2005;21:676–9. 10.1093/bioinformatics/bti079 15509596

[plaf032-B67] Qu X-J, Moore MJ, Li D-Z et al PGA: a software package for rapid, accurate, and flexible batch annotation of plastomes. Plant Methods 2019;15:1–12. 10.1186/s13007-019-0435-7 31139240 PMC6528300

[plaf032-B68] Qu X-J, Zou D, Zhang R-Y et al Progress, challenge and prospect of plant plastome annotation. Front Plant Sci 2023;14:1166140. 10.3389/fpls.2023.1166140 37324662 PMC10266425

[plaf032-B69] Raubeson LA, Jansen RK. Chloroplast genomes of plants. In: Henry D (ed.), Diversity and Evolution of Plants; Genotypic and Phenotypic Variation in Higher Plants. Cambridge, MA: CABI Publishing, 2005, 45–68.

[plaf032-B70] R Core Team . R: A Language and Environment for Statistical Computing. Vienna: R Foundation for Statistical Computing, 2023. http://www.R-project.org/

[plaf032-B71] Revell LJ . Phytools: an R package for phylogenetic comparative biology (and other things). Methods Ecol Evol 2012;3:217–23. 10.1111/j.2041-210X.2011.00169.x

[plaf032-B72] Ronquist F, Huelsenbeck JP. MRBAYES 3: Bayesian phylogenetic inference under mixed models. Bioinformatics 2003;19:1572–4. 10.1093/bioinformatics/btg180 12912839

[plaf032-B73] Rozas J, Ferrer-Mata A, Sánchez-DelBarrio JC et al DnaSP 6: DNA sequence polymorphism analysis of large data sets. Mol Biol Evol 2017;34:3299–302. 10.1093/molbev/msx248 29029172

[plaf032-B74] Ruhfel BR, Gitzendanner MA, Soltis PS et al From algae to angiosperms–inferring the phylogeny of green plants (*Viridiplantae*) from 360 plastid genomes. BMC Ecol Evol 2014;14:23. 10.1186/1471-2148-14-23 PMC3933183 24533922

[plaf032-B75] Ruhlman TA, Chang W-J, Chen JJW et al NDH expression marks major transitions in plant evolution and reveals coordinate intracellular gene loss. BMC Plant Biol 2015;15:100. 10.1186/s12870-015-0484-7 25886915 PMC4404220

[plaf032-B76] Sabir J, Schwarz E, Ellison N et al Evolutionary and biotechnology implications of plastid genome variation in the inverted repeat lacking clade of legumes. Plant Biotechnol J 2014;12:743–54. 10.1111/pbi.12179 24618204

[plaf032-B77] Sanchez-Puerta MV, Ceriotti LF, Gatica-Soria LM et al Beyond parasitic convergence: unravelling the evolution of the organellar genomes in holoparasites. Ann Bot 2023;132:909–28. 10.1093/aob/mcad108 37503831 PMC10808021

[plaf032-B78] Sanderson MJ, Copetti D, Búrquez A et al Exceptional reduction of the plastid genome of saguaro cactus (*Carnegiea gigantea*): loss of the *ndh* gene suite and inverted repeat. Am J Bot 2015;102:1115–27. 10.3732/ajb.1500184 26199368

[plaf032-B79] Schneider AC, Chun H, Stefanović S et al Punctuated plastome reduction and host-parasite horizontal gene transfer in the holoparasitic plant genus *Aphyllon*. Proc R Soc Lond B Biol Sci 2018;285:20181535. 10.1098/rspb.2018.1535 PMC6170807 30232155

[plaf032-B80] Shi C, Liu Y, Huang H et al Contradiction between plastid gene transcription and function due to complex posttranscriptional splicing: an exemplary study of *ycf15* function and evolution in angiosperms. PLoS One 2013;8:e59620. 10.1371/journal.pone.0059620 23527231 PMC3601113

[plaf032-B81] Shin HW, Lee NS. Understanding plastome evolution in Hemiparasitic Santalales: complete chloroplast genomes of three species, *Dendrotrophe varians*, *Helixanthera parasitica*, and *Macrosolen cochinchinensis*. PLoS One 2018;13:e0200293. 10.1371/journal.pone.0200293 29975758 PMC6033455

[plaf032-B82] Shinozaki K, Ohme M, Tanaka M et al The complete nucleotide sequence of the tobacco chloroplast genome: its gene organization and expression. EMBO J 1986;5:2043–9. 10.1002/j.1460-2075.1986.tb04464.x 16453699 PMC1167080

[plaf032-B83] Silva GMD, Lopes ADS, Pacheco TG et al Genetic and evolutionary analyses of plastomes of the subfamily Cactoideae (Cactaceae) indicate relaxed protein biosynthesis and tRNA import from cytosol. Rev Bras Bot 2021;44:97116. 10.1007/s40415-020-00689-2

[plaf032-B84] Stamatakis A . RAxML version 8: a tool for phylogenetic analysis and post-analysis of large phylogenies. Bioinformatics 2014;30:1312–3. 10.1093/bioinformatics/btu033 24451623 PMC3998144

[plaf032-B85] Su H-J, Barkman TJ, Hao W et al Novel genetic code and record-setting AT-richness in the highly reduced plastid genome of the holoparasitic plant *Balanophora*. Proc Natl Acad Sci U S A 2018;116:934–43. 10.1073/pnas.1816822116 30598433 PMC6338844

[plaf032-B86] Su H-J, Hu J-M. The complete chloroplast genome of hemiparasitic flowering plant *Schoepfia jasminodora*. Mitochondrial DNA B Resour 2016;1:767–9. 10.1080/23802359.2016.1238753 33473621 PMC7799538

[plaf032-B87] Su H-J, Liang SL, Nickrent DL. Plastome variation and phylogeny of *Taxillus* (Loranthaceae). PLoS One 2021;16:e0256345. 10.1371/journal.pone.0256345 34407123 PMC8372910

[plaf032-B88] Sun Y, Moore MJ, Lin N et al Complete plastome sequencing of both living species of Circaeasteraceae (Ranunculales) reveals unusual rearrangements and the loss of the *ndh* gene family. BMC Genomics 2017;18:592. 10.1186/s12864-017-3956-3 28793854 PMC5551029

[plaf032-B89] Tang L, Wang T, Hou L et al Comparative and phylogenetic analyses of Loranthaceae plastomes provide insights into the evolutionary trajectories of plastome degradation in hemiparasitic plants. BMC Plant Biol 2024;24:406. 10.1186/s12870-024-05094-5 38750463 PMC11097404

[plaf032-B90] Tiller N, Bock R. The translational apparatus of plastids and its role in plant development. Mol Plant 2014;7:1105–20. 10.1093/mp/ssu022 24589494 PMC4086613

[plaf032-B91] Tillich M, Lehwark P, Pellizzer T et al Geseq –versatile and accurate annotation of organelle genomes. Nucleic Acids Res 2017;45:W6–W11. 10.1093/nar/gkx391 28486635 PMC5570176

[plaf032-B92] Townsend JP . Profiling phylogenetic informativeness. Syst Biol 2007;56:222–31. 10.1080/10635150701311362 17464879

[plaf032-B93] Tyszka AS, Bretz EC, Robertson HM et al Characterizing conflict and congruence of molecular evolution across organellar genome sequences for phylogenetics in land plants. Front Plant Sci 2023;14:1125107. 10.3389/fpls.2023.1125107 37063179 PMC10098128

[plaf032-B94] Verma D, Daniell H. Chloroplast vector systems for biotechnology applications. Plant Physiol 2007;145:1129–43. 10.1104/pp.107.106690 18056863 PMC2151729

[plaf032-B95] Vidal-Russell R, Nickrent DL. Evolutionary relationships in the showy mistletoe family (Loranthaceae). Am J Bot 2008;95:1015–29. 10.3732/ajb.0800085 21632422

[plaf032-B96] Wakasugi T, Tsudzuki J, Ito S et al Loss of all *ndh* genes as determined by sequencing the entire chloroplast genome of the black pine *Pinus thunbergii*. Proc Natl Acad Sci U S A 1994;91:9794–8. 10.1073/pnas.91.21.9794 7937893 PMC44903

[plaf032-B97] Wang Y-H, Qu X-J, Chen S-Y et al Plastomes of Mimosoideae: structural and size variation, sequence divergence, and phylogenetic implication. Tree Genet Genomes 2017;13:41. 10.1007/s11295-017-1124-1

[plaf032-B98] Westwood JH, Yoder JI, Timko MP et al The evolution of parasitism in plants. Trends Plant Sci 2010;15:227–35. 10.1016/j.tplants.2010.01.004 20153240

[plaf032-B99] Wicke S, Müller KF, dePamphilis CW et al Mechanisms of functional and physical genome reduction in photosynthetic and nonphotosynthetic parasitic plants of the broomrape family. Plant Cell 2013;25:3711–25. 10.1105/tpc.113.113373 24143802 PMC3877813

[plaf032-B100] Wicke S, Müller KF, dePamphilis CW et al Mechanistic model of evolutionary rate variation en route to a nonphotosynthetic lifestyle in plants. Proc Natl Acad Sci U S A 2016;113:9045–50. 10.1073/pnas.1607576113 27450087 PMC4987836

[plaf032-B101] Wickham H . ggplot2: Elegant Graphics for Data Analysis. New York, NY: Springer, 2016.

[plaf032-B102] Wolfe KH, Morden CW, Ems SC et al Rapid evolution of the plastid translational apparatus in a nonphotosynthetic plant: loss or accelerated sequence evolution of tRNA and ribosomal protein genes. J Mol Evol 1992a;35:304–17. 10.1007/BF00161168 1404416

[plaf032-B103] Wolfe KH, Morden CW, Palmer JD. Function and evolution of a minimal plastid genome from a nonphotosynthetic parasitic plant. Proc Natl Acad Sci U S A 1992b;89:10648–52. 10.1073/pnas.89.22.10648 1332054 PMC50398

[plaf032-B104] Wu C, Lai Y, Lin C et al Evolution of reduced and compact plastid genomes (cpDNAs) in gnetophytes: selection towards a low-cost strategy. Mol Phylogenet Evol 2009;52:115–24. 10.1016/j.ympev.2008.12.026 19166950

[plaf032-B105] Xiao-Ming Z, Junrui W, Li F et al Inferring the evolutionary mechanism of the chloroplast genome size by comparing whole-chloroplast genome sequences in seed plants. Sci Rep 2017;7:1555. 10.1038/s41598-017-01518-5 28484234 PMC5431534

[plaf032-B106] Yang J, Park S, Gil H-Y et al Characterization and dynamics of intracellular gene transfer in plastid genomes of *Viola* (Violaceae) and Order Malpighiales. Front Plant Sci 2021;12:678580. 10.3389/fpls.2021.678580 34512682 PMC8429499

[plaf032-B107] Yao X, Tang P, Li Z et al The first complete chloroplast genome sequences in Actinidiaceae: genome structure and comparative analysis. PLoS One 2015;10:e0129347. 10.1371/journal.pone.0129347 26046631 PMC4457681

[plaf032-B108] Zhang C, Lin Q, Zhang J et al Comparing complete organelle genomes of holoparasitic *Christisonia kwangtungensis* (Orabanchaceae) with its close relatives: how different are they? BMC Plant Biol 2022;22:444. 10.1186/s12870-022-03814-3 36114450 PMC9482287

[plaf032-B109] Zhang X, Sun Y, Landis JB et al Plastome phylogenomic study of Gentianeae (Gentianaceae): widespread gene tree discordance and its association with evolutionary rate heterogeneity of plastid genes. BMC Plant Biol 2020b;20:340. 10.1186/s12870-020-02518-w 32680458 PMC7368685

[plaf032-B110] Zhang R, Xu B, Li J et al Transit from autotrophism to heterotrophism: sequence variation and evolution of chloroplast genomes in Orobanchaceae species. Front Genet 2020a;11:542017. 10.3389/fgene.2020.542017 33133143 PMC7573133

[plaf032-B111] Zhao C, Wang Y, Chan KX et al Evolution of chloroplast retrograde signaling facilitates green plant adaptation to land. Proc Natl Acad Sci U S A 2019;116:5015–20. 10.1073/pnas.1812092116 30804180 PMC6421419

[plaf032-B112] Zhu Z-X, Wang J-H, Cai Y-C et al Complete plastome sequence of *Erythropalum scandens* (Erythropalaceae), an edible and medicinally important liana in China. Mitochondrial DNA B Resour 2018;3:139–40. 10.1080/23802359.2017.1413435 33474097 PMC7799593

