# Supplementary material for: Comparative plastomes of five Psittacanthus species: genome organization, structural features, and patterns of pseudogenization and gene loss
Source: AoB Plants. 2025 Jun 24;17(4):plaf032. doi: 10.1093/aobpla/plaf032 (PMC12342154; doi:10.1093/aobpla/plaf032)
Supplement: plaf032_Supplementary_Data [file plaf032_supplementary_data.pdf]

Online Supporting Information: Tables S1–S3 and Figures S1–S3

Comparative plastomes of five *Psittacanthus* species: genome organization, structural features, and patterns of pseudogenization and gene loss

By

Table S1. Sampling locations of *Psittacanthus* species studied in this work.

Species	Locality	Altitude (m)	Latitude N	Longitude W	Voucher information
P. auriculatus	Oaxaca, Santiago Chazumba	1709	18°05´42´´	97°41´11´´	M.J. Pérez CPC01
P. palmeri	Oaxaca, Sta. Ma. Tecomavaca, C. Sabino	725	17°51´53´´	97°02´10´´	C. Soberanes CPC139
P. rhynchanthus	Michoacán, La Peña	9	18°10´43´´	102°28´08´´	E. Ruiz Sánchez 417
P. schiedeanus	Oaxaca, Santiago Comaltepec	848	17°41´23´´	96°20´13´´	A. Ortiz-Rodríguez 754
P. sonorae	Sonora, Paraiso La Manga	7	27°53´43´´	111°06´55´´	F. Molina-Freaner

Table S2. GenBank accession numbers and reference information for species used in this study.

Species	GenBank Accession Number	Reference
Amyema miraculosa	OK377284	Not published
Amyema miquelii	OK377282	Not published
Amyema pendula	OK377285	Not published
Amyema preissii	OK377287	Not published
Amyema quandang	OK377272	Not published
Cecarria obtusifolia	MT987627	Nickrent *et al*. 2021
Dendrophthoe pentandra	MN175255	Guo and Ruan 2019
Elytranthe albida	MN175256	Guo and Ruan 2019
Elytranthe parasitica	OR909696	Tang *et al*. 2024
Helicanthes elasticus	OM974141	Darshetkar *et al*. 2023
Helixanthera parasitica	MN080718	Liu *et al*. 2019
Helixanthera sampsonii	OR909690	Tang *et al*. 2024
Helixanthera terrestris	OR909695	Tang *et al*. 2024
Loranthus delavayi	MT987624	Nickrent *et al*. 2021
Loranthus europaeus	MT987629	Nickrent *et al*. 2021
Loranthus grewingkii	MT987631	Nickrent *et al*. 2021
Loranthus guizhouensis	MT987632	Nickrent *et al*. 2021
Loranthus kaoi	MT987633	Nickrent *et al*. 2021
Loranthus lambertianus	MT987634	Nickrent *et al*. 2021
Loranthus odoratus	MT987636	Nickrent *et al*. 2021
Loranthus pseudo-odoratus	NC058865	Nickrent *et al*. 2021
Loranthus tanakae	NC058867	Nickrent *et al*. 2021
Lysiana exocarpi	OK377275	Not published
Macrosolen bibracteolatus	MH161423	Nie *et al*. 2019
Macrosolen cochinchinensis	MH161424	Nie *et al*. 2019
Macrosolen tricolor	MH161425	Nie *et al*. 2019
Moquiniella rubra	MT987639	Nickrent *et al*. 2021
Muellerina celastroides	OK377277	Not published
Muellerina eucalyptoides	OK377278	Not published
Nuytsia floribunda	MT987640	Nickrent *et al*. 2021
Plicosepalus acaciae	NC068660	Al-Juhani *et al*. 2023
Plicosepalus curviflorus	NC068661	Al-Juhani *et al*. 2023
Psittacanthus auriculatus	PP236145	This study
Psittacanthus palmeri	PP236144	This study
Psittacanthus rhynchanthus	PP579889	This study
Psittacanthus schiedeanus	OR701826	Morales-Saldaña *et al*. 2024
Psittacanthus sonorae	PP313080	This study
Scurrula atropurpurea	OR909711	Tang *et al*. 2024
Scurrula buddleioides	OR909710	Tang *et al*. 2024

Scurrula chingii	NC053563	Li *et al*. 2021
Scurrula notothixoides	NC041305	Yuan *et al*. 2018
Scurrula parasitica	NC040862	Yue *et al*. 2023
Scurrula pulverulenta	OR909701	Tang *et al*. 2024
Taxillus balansae	OR909691	Tang *et al*. 2024
Taxillus calorea	OR909702	Tang *et al*. 2024
Taxillus chinensis	KY996492	Li *et al*. 2017; Liu *et al*. 2019
Taxillus levinei	NC058836	Su *et al*. 2021
Taxillus liquidambaricola	MW598494	Su *et al*. 2021
Taxillus lonicerifolius	MW598501	Su *et al*. 2021
Taxillus matsudae	NC058842	Su *et al*. 2021
Taxillus pseudochinensis	MW598493	Su *et al*. 2021
Taxillus rhododendricola	MW598498	Su *et al*. 2021
Taxillus sericus	OR909693	Tang *et al*. 2024
Taxillus sutchuenensis	KY996493	Ying *et al*. 2017
Taxillus tsaii	MW598497	Su *et al*. 2021
Taxillus theifer	MW598504	Su *et al*. 2021
Taxillus thibetensis	OR909692	Tang *et al*. 2024
Taxillus vestitus	MN175257	Guo *et al*. 2019
Taxillus yadoriki	MT702883	Cho *et al*. 2020
Tolypanthus maclurei	MH922027	Yu *et al*. 2018
Erythropalum scandens	MG661263	Zhu *et al*. 2018
Schoepfia jasminodora	KX775962	Su and Hu 2016

References

Al-Juhani W, Al-Thagafi NT, Al-Qthanin RN. 2022. Gene losses and plastome degradation in the hemiparasitic species *Plicosepalus acaciae* and *Plicosepalus curviflorus*: comparative analyses and phylogenetic relationships among Santalales members. *Plants* 11:1869.

Cho WB, Han EK, Son DC, Lee JH. 2020. The complete chloroplast genome sequence of *Taxillus yadoriki* (Loranthaceae): a hemi-parasitic evergreen shrub in east Asia. *Mitochondrial DNA Part B* 5:3172–3173.

Darshetkar AM, Pable AA, Nadaf AB, Barvkar VT. 2023. Understanding parasitism in Loranthaceae: insights from plastome and mitogenome of *Helicanthes elastica*. *Gene* 861:147238.

Guo X, Ruan Z. 2019. Characterization of the complete plastome of *Dendrophthoe pentandra* (Loranthaceae), a stem hemiparasite. *Mitochondrial DNA Part B* 4:3099–3100.

Guo X, Ruan Z, Zhang G. 2019. The complete plastome of *Taxillus vestitus* (Loranthaceae), a hemiparasitic plant. *Mitochondrial DNA Part B* 4:3188–3189.

Li M, Zhang Y, Li Y, Zhang L. 2021. The complete chloroplast genome of *Scurrula chingii* (W.C. Cheng) H.S. Kiu (Loranthaceae), a hemiparasitic shrub. *Mitochondrial DNA Part B* 6:282–284.

Li Y, Zhou JG, Chen XL, Cui YX, Xu ZC, Li YH, Song JY, *et al*. 2017. Gene losses and partial deletion of small single-copy regions of the chloroplast genomes of two hemiparasitic *Taxillus* species. *Scientific Reports* 7:12834.

Liu B, Zhang Y, Shi Y. 2019. Complete chloroplast genome sequence of *Taxillus chinensis* (Loranthaceae): a hemiparasitic shrub in South China. *Mitochondrial DNA Part B* 4:3077–3078.

Morales-Saldaña S, Villafán E, Vásquez-Aguilar AA, Ramírez-Barahona S, Ibarra-Laclette E, Ornelas JF. 2024. The complete chloroplast genome sequence of *Psittacanthus schiedeanus* (Cham. & Schltdl.) G. Don (Santalales: Loranthaceae), the first plastome of a mistletoe species in the Psittacantheae tribe. *Mitochondrial DNA Part B* 9:5–10.

Nickrent DL, Su HJ, Lin RZ, Devkota MP, Hu JM, Glatzel G. 2021. Examining the needle in the haystack: evolutionary relationships in the mistletoe genus *Loranthus* (Loranthaceae). *Systematic Botany* 46:403–415.

Shi Y, Zhang Y, Liu B. 2019. The complete chloroplast genome sequence of *Scurrula parasitica* (Loranthaceae). *Mitochondrial DNA Part B* 4:3029–3030.

Shin HW, Lee NS. 2018. Understanding plastome evolution in Hemiparasitic Santalales: complete chloroplast genomes of three species, *Dendrotrophe varians*, *Helixanthera parasitica*, and *Macrosolen cochinchinensis*. *PLoS ONE* 13:e0200293.

Su HJ, Liang SL, Nickrent DL. 2021. Plastome variation and phylogeny of *Taxillus* (Loranthaceae). *PLoS ONE* 16:e0256345.

Yu R, Zhou S, Zhou Q, Liu Y, Zhou R. 2018. The complete chloroplast genome of a hemiparasitic plant *Tolypanthus maclurei* (Loranthaceae). *Mitochondrial DNA Part B* 4:207–208.

Yuan LX, Wang JH, Chen CR, Zhao KK, Zhu ZX, Wang HF. 2018. Complete chloroplast genome sequence of *Scurrula notothixoides* (Loranthaceae): a hemiparasitic shrub in South China. *Mitochondrial DNA Part B* 3:580–581.

Table S3. Results from the estimation substitution model conducted by ModelTest-NG.

AIC	model	K	lnL	score	delta	weight
1	GTR+I+G4	10	–277777.178	555784.3573	0.000	0.9998
2	TVM+I+G4	9	–277786.607	555801.2142	16.856	0.0002
3	TPM1uf+I+G4	7	–277930.497	556084.9944	300.637	0.0000
4	TIM1+I+G4	8	–277944.333	556114.6677	330.310	0.0000
5	GTR+G4	9	–277984.860	556197.7207	413.363	0.0000
6	TVM+G4	8	–277993.961	556213.9223	429.565	0.0000
7	TIM1+G4	7	–278161.832	556547.6651	763.307	0.0000
8	TPM1uf+G4	6	–278164.792	556551.5843	767.227	0.0000
9	GTR+I	9	–279076.437	558380.8748	2596.517	0.0000
10	TVM+I	8	–279081.140	558388.2813	2603.924	0.0000

Figure S1. Ancestral state reconstruction of pseudogenization and loss of *infA*, *ndhB*, *ndhD*, *ndhF*, *rpl16*, *trnA*, *trnL*, *trnK*, and *ycf15* genes in Loranthaceae using the equal-rates (ER) likelihood model. The plastid genes were classified into three types (intact, pseudogenization, loss). Light blue indicates a functional gene; dark blue indicates the gene becomes pseudogenized; black indicates gene loss.

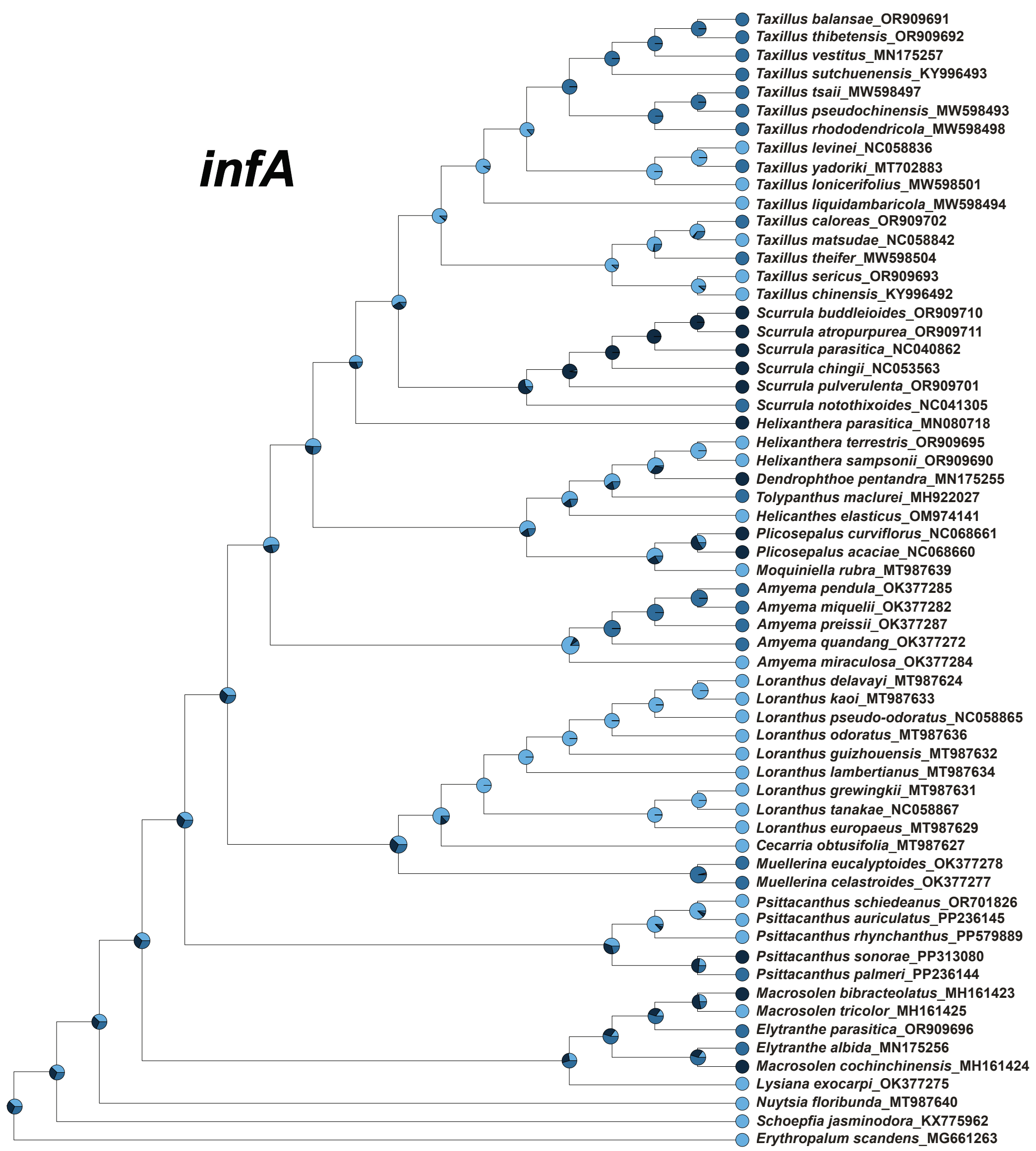

infA
Taxillus balansae_OR909691
Taxillus thibetensis_OR909692
Taxillus vestitus_MN175257
Taxillus sutchuenensis_KY996493
Taxillus tsaii_MW598497
Taxillus pseudochinensis_MW598493
Taxillus rhododendricola_MW598498
Taxillus levinei_NC058836
Taxillus yadoriki_MT702883
Taxillus lonicerifolius_MW598501
Taxillus liquidambaricola_MW598494
Taxillus caloreas_OR909702
Taxillus matsudae_NC058842
Taxillus theifer_MW598504
Taxillus sericus_OR909693
Taxillus chinensis_KY996492
Scurrula buddleioides_OR909710
Scurrula atropurpurea_OR909711
Scurrula parasitica_NC040862
Scurrula chingii_NC053563
Scurrula pulverulenta_OR909701
Scurrula notothixoides_NC041305
Helixanthera parasitica_MN080718
Helixanthera terrestris_OR909695
Helixanthera sampsonii_OR909690
Dendrophthoe pentandra_MN175255
Tolypanthus maclurei_MH922027
Helicanthes elasticus_OM974141
Plicosepalus curviflorus_NC068661
Plicosepalus acaciae_NC068660
Moquiniella rubra_MT987639
Amyema pendula_OK377285
Amyema miquelii_OK377282
Amyema preissii_OK377287
Amyema quandang_OK377272
Amyema miraculosa_OK377284
Loranthus delavayi_MT987624
Loranthus kaoi_MT987633
Loranthus pseudo-odoratus_NC058865
Loranthus odoratus_MT987636
Loranthus guizhouensis_MT987632
Loranthus lambertianus_MT987634
Loranthus grewingkii_MT987631
Loranthus tanakae_NC058867
Loranthus europaeus_MT987629
Cecarria obtusifolia_MT987627
Muellerina eucalyptoides_OK377278
Muellerina celastroides_OK377277
Psittacanthus schiedeanus_OR701826
Psittacanthus auriculatus_PP236145
Psittacanthus rhynchanthus_PP579889
Psittacanthus sonorae_PP313080
Psittacanthus palmeri_PP236144
Macrosolen bibracteolatus_MH161423
Macrosolen tricolor_MH161425
Elytranthe parasitica_OR909696
Elytranthe albida_MN175256
Macrosolen cochinchinensis_MH161424
Lysiana exocarpi_OK377275
Nuytsia floribunda_MT987640
Schoepfia jasminodora_KX775962
Erythropalum scandens_MG661263

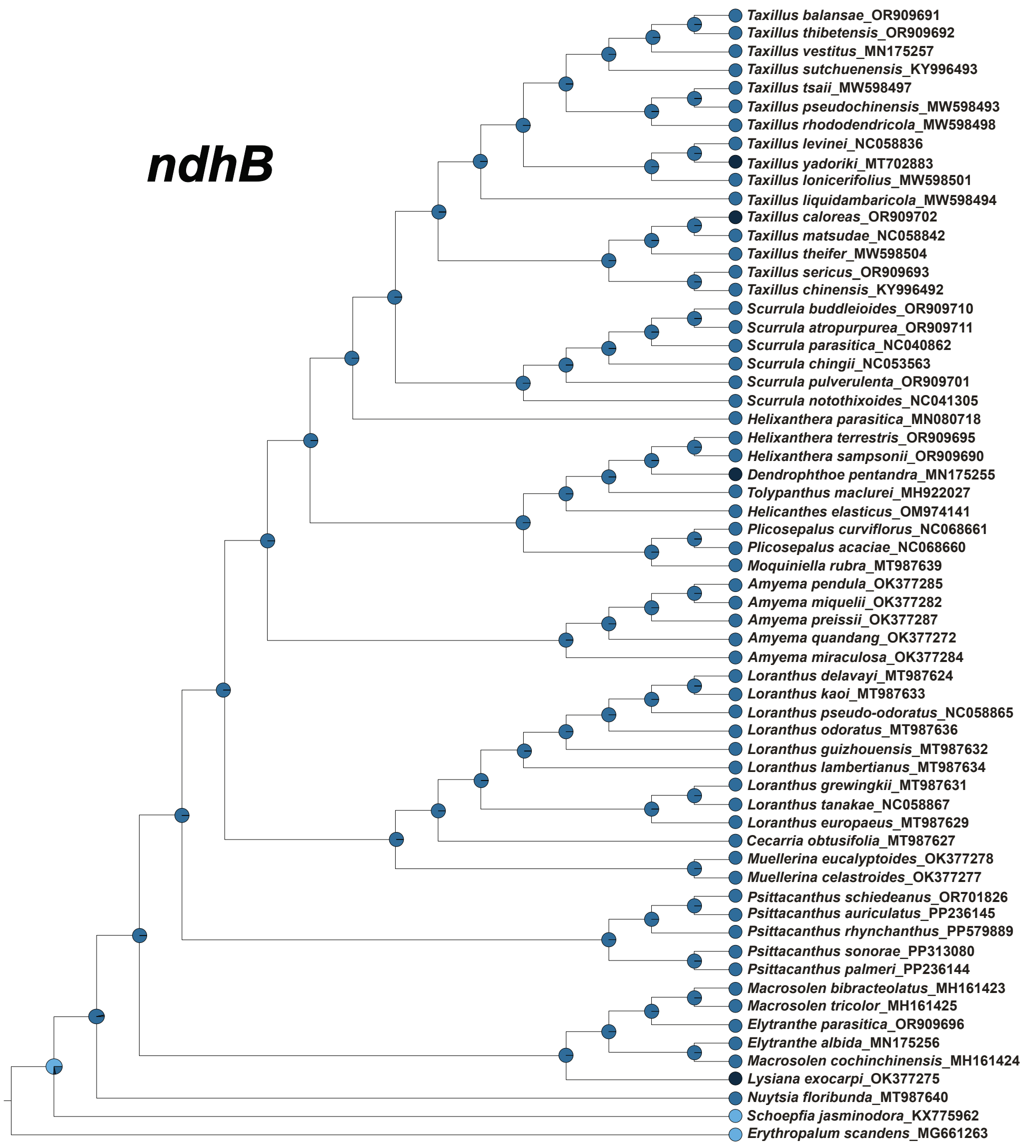
ndhB
Taxillus balansae_OR909691
Taxillus thibetensis_OR909692
Taxillus vestitus_MN175257
Taxillus sutchuenensis_KY996493
Taxillus tsaii_MW598497
Taxillus pseudochinensis_MW598493
Taxillus rhododendricola_MW598498
Taxillus levinei_NC058836
Taxillus yadoriki_MT702883
Taxillus lonicerifolius_MW598501
Taxillus liquidambaricola_MW598494
Taxillus caloreas_OR909702
Taxillus matsudae_NC058842
Taxillus theifer_MW598504
Taxillus sericus_OR909693
Taxillus chinensis_KY996492
Scurrula buddleioides_OR909710
Scurrula atropurpurea_OR909711
Scurrula parasitica_NC040862
Scurrula chingii_NC053563
Scurrula pulverulenta_OR909701
Scurrula notothixoides_NC041305
Helixanthera parasitica_MN080718
Helixanthera terrestris_OR909695
Helixanthera sampsonii_OR909690
Dendrophthoe pentandra_MN175255
Tolypanthus maclurei_MH922027
Helicanthes elasticus_OM974141
Plicosepalus curviflorus_NC068661
Plicosepalus acaciae_NC068660
Moquiniella rubra_MT987639
Amyema pendula_OK377285
Amyema miquelii_OK377282
Amyema preissii_OK377287
Amyema quandang_OK377272
Amyema miraculosa_OK377284
Loranthus delavayi_MT987624
Loranthus kaoi_MT987633
Loranthus pseudo-odoratus_NC058865
Loranthus odoratus_MT987636
Loranthus guizhouensis_MT987632
Loranthus lambertianus_MT987634
Loranthus grewingkii_MT987631
Loranthus tanakae_NC058867
Loranthus europaeus_MT987629
Cecarria obtusifolia_MT987627
Muellerina eucalyptoides_OK377278
Muellerina celastroides_OK377277
Psittacanthus schiedeanus_OR701826
Psittacanthus auriculatus_PP236145
Psittacanthus rhynchanthus_PP579889
Psittacanthus sonorae_PP313080
Psittacanthus palmeri_PP236144
Macrosolen bibracteolatus_MH161423
Macrosolen tricolor_MH161425
Elytranthe parasitica_OR909696
Elytranthe albida_MN175256
Macrosolen cochinchinensis_MH161424
Lysiana exocarpi_OK377275
Nuytsia floribunda_MT987640
Schoepfia jasminodora_KX775962
Erythropalum scandens_MG661263

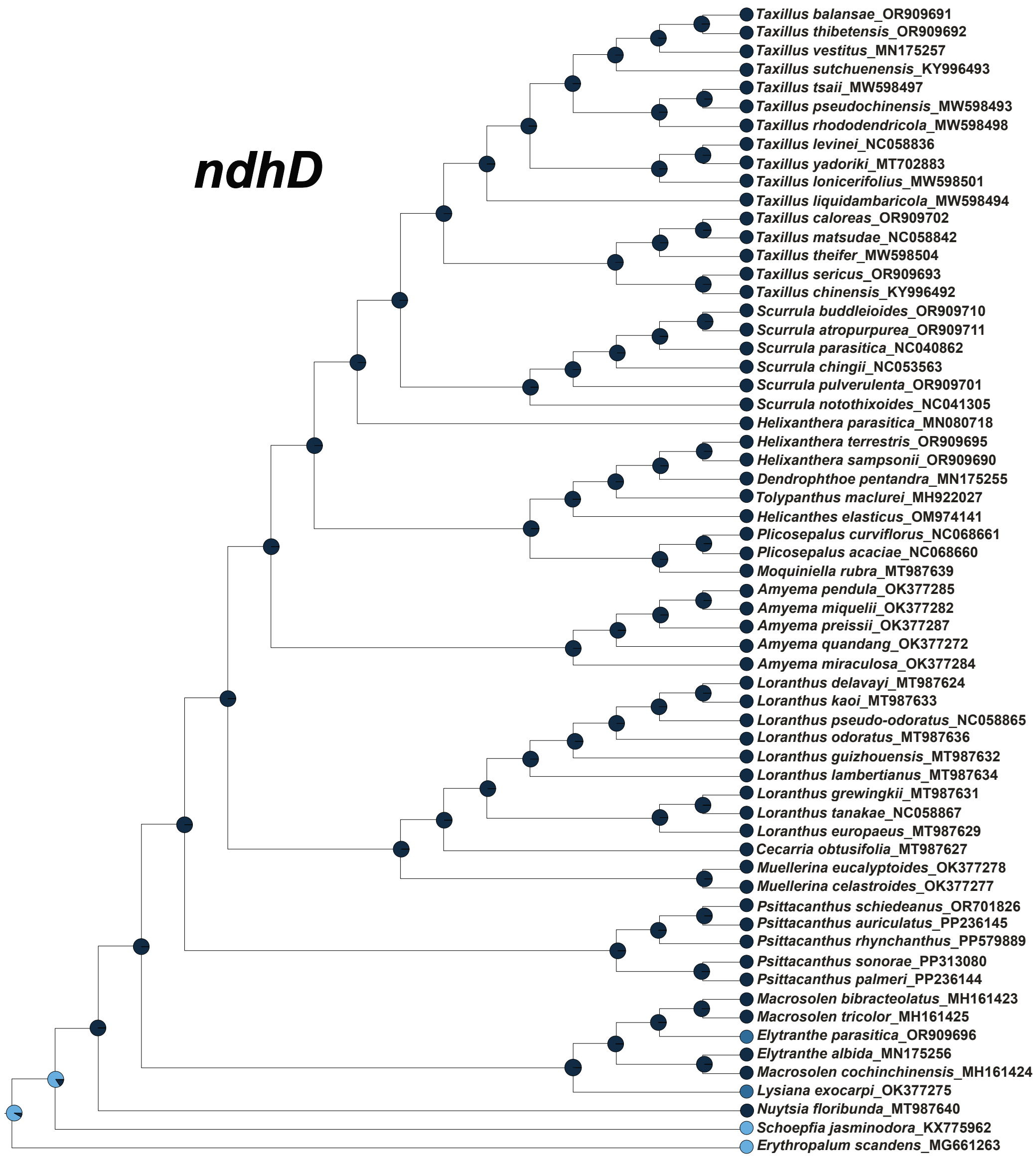

ndhD
Taxillus balansae_OR909691
Taxillus thibetensis_OR909692
Taxillus vestitus_MN175257
Taxillus sutchuenensis_KY996493
Taxillus tsaii_MW598497
Taxillus pseudochinensis_MW598493
Taxillus rhododendricola_MW598498
Taxillus levinei_NC058836
Taxillus yadoriki_MT702883
Taxillus lonicerifolius_MW598501
Taxillus liquidambaricola_MW598494
Taxillus caloreas_OR909702
Taxillus matsudae_NC058842
Taxillus theifer_MW598504
Taxillus sericus_OR909693
Taxillus chinensis_KY996492
Scurrula buddleioides_OR909710
Scurrula atropurpurea_OR909711
Scurrula parasitica_NC040862
Scurrula chingii_NC053563
Scurrula pulverulenta_OR909701
Scurrula notothixoides_NC041305
Helixanthera parasitica_MN080718
Helixanthera terrestris_OR909695
Helixanthera sampsonii_OR909690
Dendrophthoe pentandra_MN175255
Tolypanthus maclurei_MH922027
Helicanthes elasticus_OM974141
Plicosepalus curviflorus_NC068661
Plicosepalus acaciae_NC068660
Moquiniella rubra_MT987639
Amyema pendula_OK377285
Amyema miquelii_OK377282
Amyema preissii_OK377287
Amyema quandang_OK377272
Amyema miraculosa_OK377284
Loranthus delavayi_MT987624
Loranthus kaoi_MT987633
Loranthus pseudo-odoratus_NC058865
Loranthus odoratus_MT987636
Loranthus guizhouensis_MT987632
Loranthus lambertianus_MT987634
Loranthus grewingkii_MT987631
Loranthus tanakae_NC058867
Loranthus europaeus_MT987629
Cecarria obtusifolia_MT987627
Muellerina eucalyptoides_OK377278
Muellerina celastroides_OK377277
Psittacanthus schiedeanus_OR701826
Psittacanthus auriculatus_PP236145
Psittacanthus rhynchanthus_PP579889
Psittacanthus sonorae_PP313080
Psittacanthus palmeri_PP236144
Macrosolen bibracteolatus_MH161423
Macrosolen tricolor_MH161425
Elytranthe parasitica_OR909696
Elytranthe albida_MN175256
Macrosolen cochinchinensis_MH161424
Lysiana exocarpi_OK377275
Nuytsia floribunda_MT987640
Schoepfia jasminodora_KX775962
Erythropalum scandens_MG661263

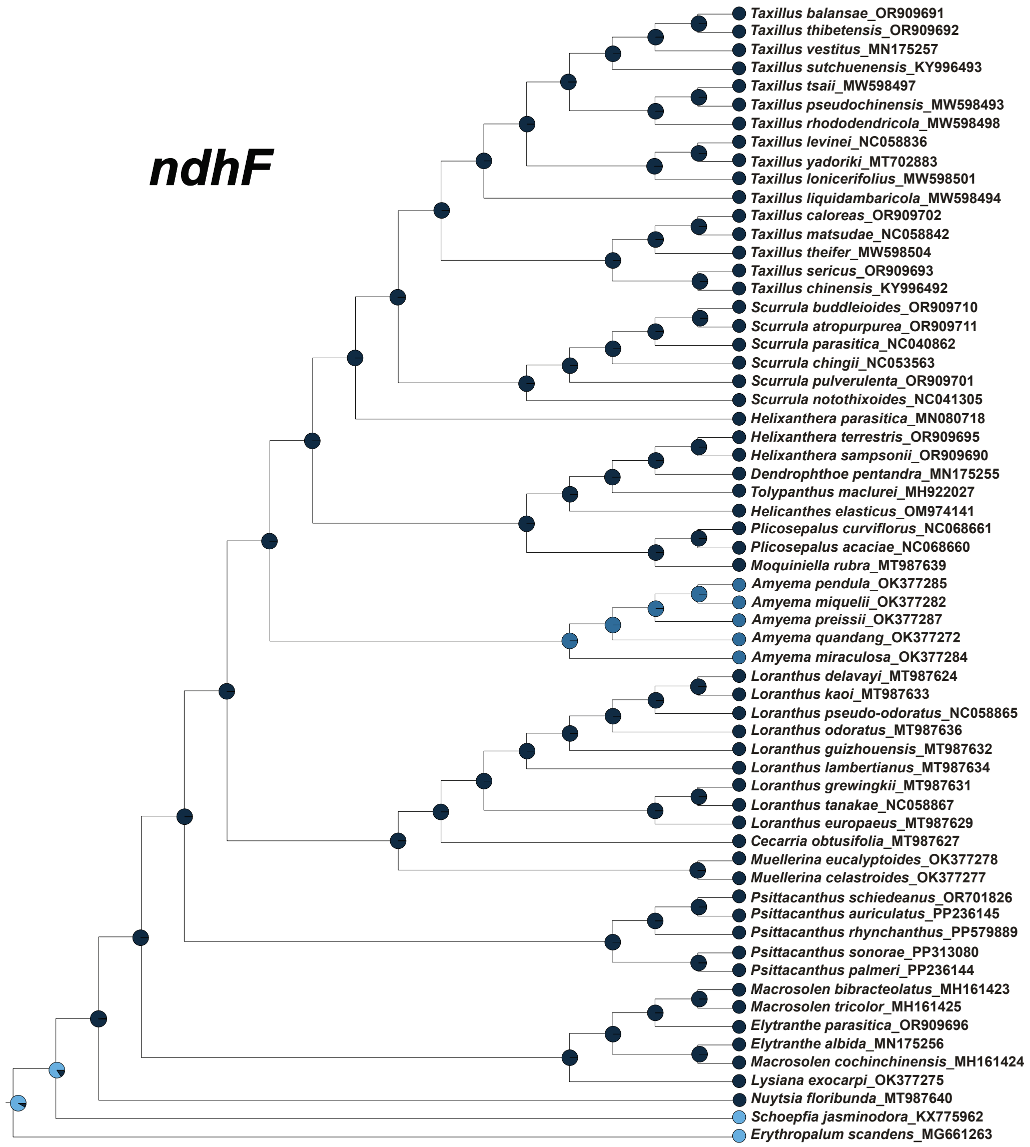

ndhF
Taxillus balansae_OR909691
Taxillus thibetensis_OR909692
Taxillus vestitus_MN175257
Taxillus sutchuenensis_KY996493
Taxillus tsaii_MW598497
Taxillus pseudochinensis_MW598493
Taxillus rhododendricola_MW598498
Taxillus levinei_NC058836
Taxillus yadoriki_MT702883
Taxillus lonicerifolius_MW598501
Taxillus liquidambaricola_MW598494
Taxillus caloreas_OR909702
Taxillus matsudae_NC058842
Taxillus theifer_MW598504
Taxillus sericus_OR909693
Taxillus chinensis_KY996492
Scurrula buddleioides_OR909710
Scurrula atropurpurea_OR909711
Scurrula parasitica_NC040862
Scurrula chingii_NC053563
Scurrula pulverulenta_OR909701
Scurrula notothixoides_NC041305
Helixanthera parasitica_MN080718
Helixanthera terrestris_OR909695
Helixanthera sampsonii_OR909690
Dendrophthoe pentandra_MN175255
Tolypanthus maclurei_MH922027
Helicanthes elasticus_OM974141
Plicosepalus curviflorus_NC068661
Plicosepalus acaciae_NC068660
Moquiniella rubra_MT987639
Amyema pendula_OK377285
Amyema miquelii_OK377282
Amyema preissii_OK377287
Amyema quandang_OK377272
Amyema miraculosa_OK377284
Loranthus delavayi_MT987624
Loranthus kaoi_MT987633
Loranthus pseudo-odoratus_NC058865
Loranthus odoratus_MT987636
Loranthus guizhouensis_MT987632
Loranthus lambertianus_MT987634
Loranthus grewingkii_MT987631
Loranthus tanakae_NC058867
Loranthus europaeus_MT987629
Cecarria obtusifolia_MT987627
Muellerina eucalyptoides_OK377278
Muellerina celastroides_OK377277
Psittacanthus schiedeanus_OR701826
Psittacanthus auriculatus_PP236145
Psittacanthus rhynchanthus_PP579889
Psittacanthus sonorae_PP313080
Psittacanthus palmeri_PP236144
Macrosolen bibracteolatus_MH161423
Macrosolen tricolor_MH161425
Elytranthe parasitica_OR909696
Elytranthe albida_MN175256
Macrosolen cochinchinensis_MH161424
Lysiana exocarpi_OK377275
Nuytsia floribunda_MT987640
Schoepfia jasminodora_KX775962
Erythropalum scandens_MG661263

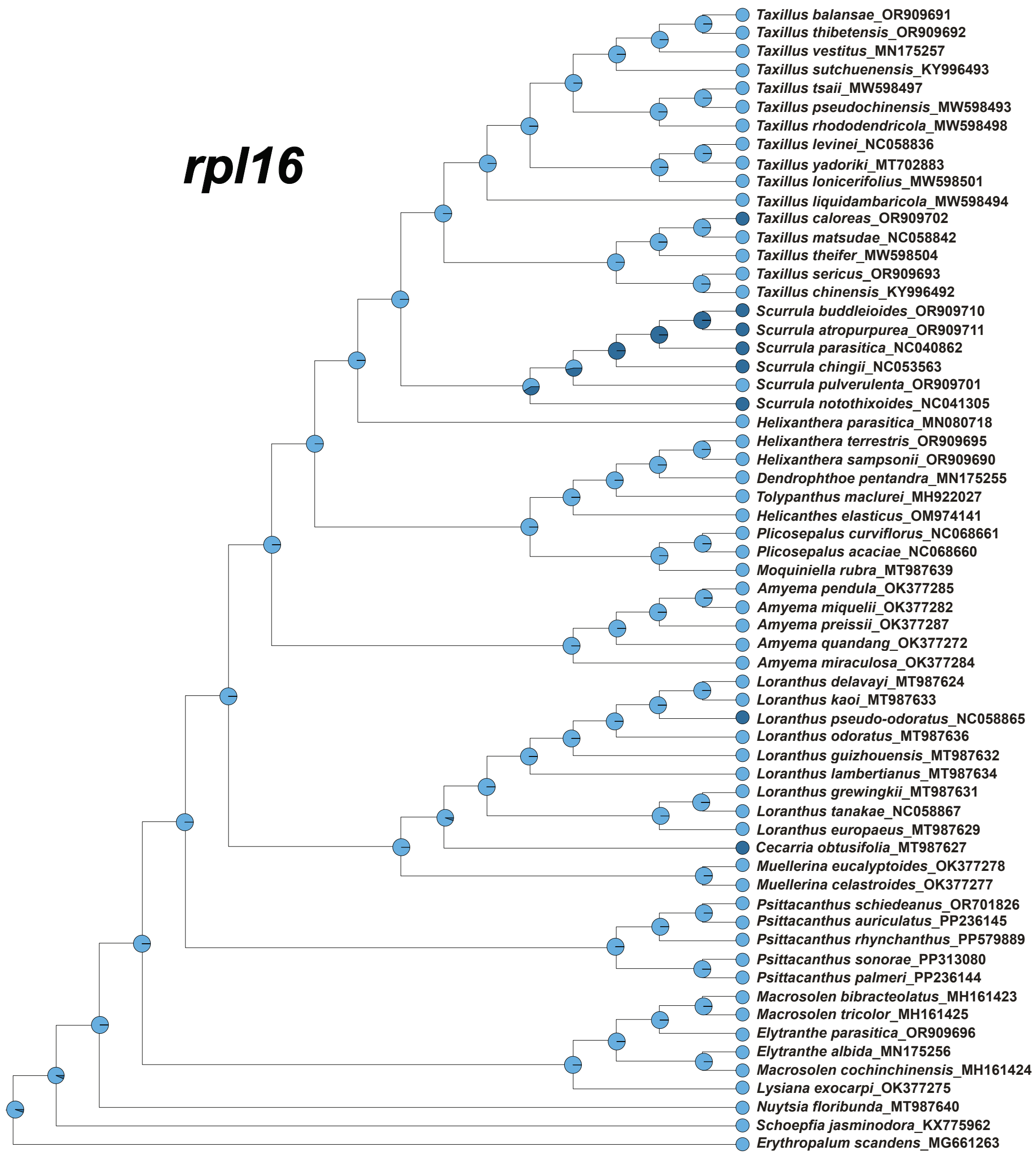

rpl16
Taxillus balansae_OR909691
Taxillus thibetensis_OR909692
Taxillus vestitus_MN175257
Taxillus sutchuenensis_KY996493
Taxillus tsaii_MW598497
Taxillus pseudochinensis_MW598493
Taxillus rhododendricola_MW598498
Taxillus levinei_NC058836
Taxillus yadoriki_MT702883
Taxillus lonicerifolius_MW598501
Taxillus liquidambaricola_MW598494
Taxillus caloreas_OR909702
Taxillus matsudae_NC058842
Taxillus theifer_MW598504
Taxillus sericus_OR909693
Taxillus chinensis_KY996492
Scurrula buddleioides_OR909710
Scurrula atropurpurea_OR909711
Scurrula parasitica_NC040862
Scurrula chingii_NC053563
Scurrula pulverulenta_OR909701
Scurrula notothixoides_NC041305
Helixanthera parasitica_MN080718
Helixanthera terrestris_OR909695
Helixanthera sampsonii_OR909690
Dendrophthoe pentandra_MN175255
Tolypanthus maclurei_MH922027
Helicanthes elasticus_OM974141
Plicosepalus curviflorus_NC068661
Plicosepalus acaciae_NC068660
Moquiniella rubra_MT987639
Amyema pendula_OK377285
Amyema miquelii_OK377282
Amyema preissii_OK377287
Amyema quandang_OK377272
Amyema miraculosa_OK377284
Loranthus delavayi_MT987624
Loranthus kaoi_MT987633
Loranthus pseudo-odoratus_NC058865
Loranthus odoratus_MT987636
Loranthus guizhouensis_MT987632
Loranthus lambertianus_MT987634
Loranthus grewingkii_MT987631
Loranthus tanakae_NC058867
Loranthus europaeus_MT987629
Cecarria obtusifolia_MT987627
Muellerina eucalyptoides_OK377278
Muellerina celastroides_OK377277
Psittacanthus schiedeanus_OR701826
Psittacanthus auriculatus_PP236145
Psittacanthus rhynchanthus_PP579889
Psittacanthus sonorae_PP313080
Psittacanthus palmeri_PP236144
Macrosolen bibracteolatus_MH161423
Macrosolen tricolor_MH161425
Elytranthe parasitica_OR909696
Elytranthe albida_MN175256
Macrosolen cochinchinensis_MH161424
Lysiana exocarpi_OK377275
Nuytsia floribunda_MT987640
Schoepfia jasminodora_KX775962
Erythropalum scandens_MG661263

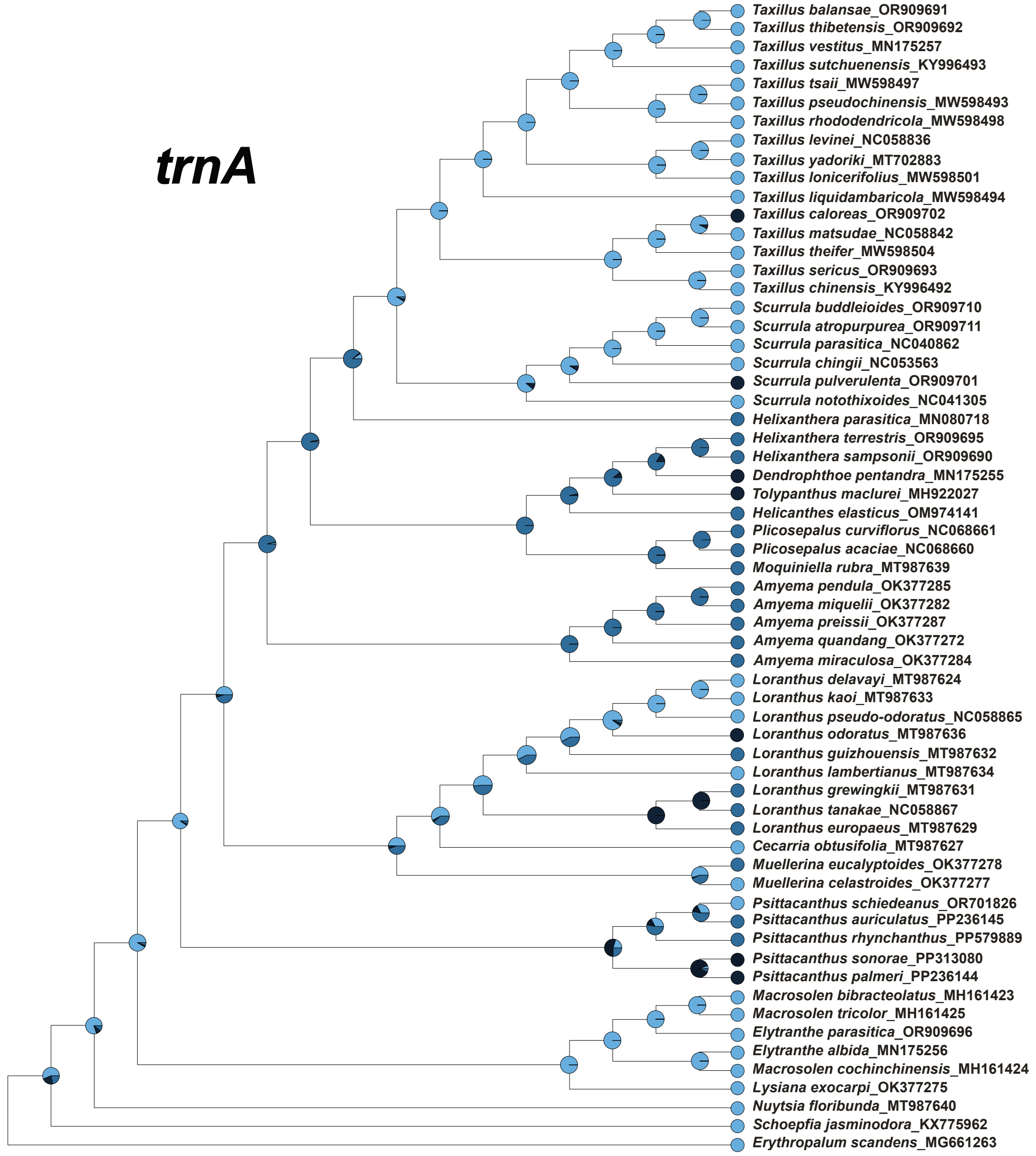
trnA
Taxillus balansae_OR909691
Taxillus thibetensis_OR909692
Taxillus vestitus_MN175257
Taxillus sutchuenensis_KY996493
Taxillus tsaii_MW598497
Taxillus pseudochinensis_MW598493
Taxillus rhododendricola_MW598498
Taxillus levinei_NC058836
Taxillus yadoriki_MT702883
Taxillus lonicerifolius_MW598501
Taxillus liquidambaricola_MW598494
Taxillus caloreas_OR909702
Taxillus matsudae_NC058842
Taxillus theifer_MW598504
Taxillus sericus_OR909693
Taxillus chinensis_KY996492
Scurrula buddleioides_OR909710
Scurrula atropurpurea_OR909711
Scurrula parasitica_NC040862
Scurrula chingii_NC053563
Scurrula pulverulenta_OR909701
Scurrula notothixoides_NC041305
Helixanthera parasitica_MN080718
Helixanthera terrestris_OR909695
Helixanthera sampsonii_OR909690
Dendrophthoe pentandra_MN175255
Tolypanthus maclurei_MH922027
Helicanthes elasticus_OM974141
Plicosepalus curviflorus_NC068661
Plicosepalus acaciae_NC068660
Moquiniella rubra_MT987639
Amyema pendula_OK377285
Amyema miquelii_OK377282
Amyema preissii_OK377287
Amyema quandang_OK377272
Amyema miraculosa_OK377284
Loranthus delavayi_MT987624
Loranthus kaoi_MT987633
Loranthus pseudo-odoratus_NC058865
Loranthus odoratus_MT987636
Loranthus guizhouensis_MT987632
Loranthus lambertianus_MT987634
Loranthus grewingkii_MT987631
Loranthus tanakae_NC058867
Loranthus europaeus_MT987629
Cecarria obtusifolia_MT987627
Muellerina eucalyptoides_OK377278
Muellerina celastroides_OK377277
Psittacanthus schiedeanus_OR701826
Psittacanthus auriculatus_PP236145
Psittacanthus rhynchanthus_PP579889
Psittacanthus sonorae_PP313080
Psittacanthus palmeri_PP236144
Macrosolen bibracteolatus_MH161423
Macrosolen tricolor_MH161425
Elytranthe parasitica_OR909696
Elytranthe albida_MN175256
Macrosolen cochinchinensis_MH161424
Lysiana exocarpi_OK377275
Nuytsia floribunda_MT987640
Schoepfia jasminodora_KX775962
Erythropalum scandens_MG661263

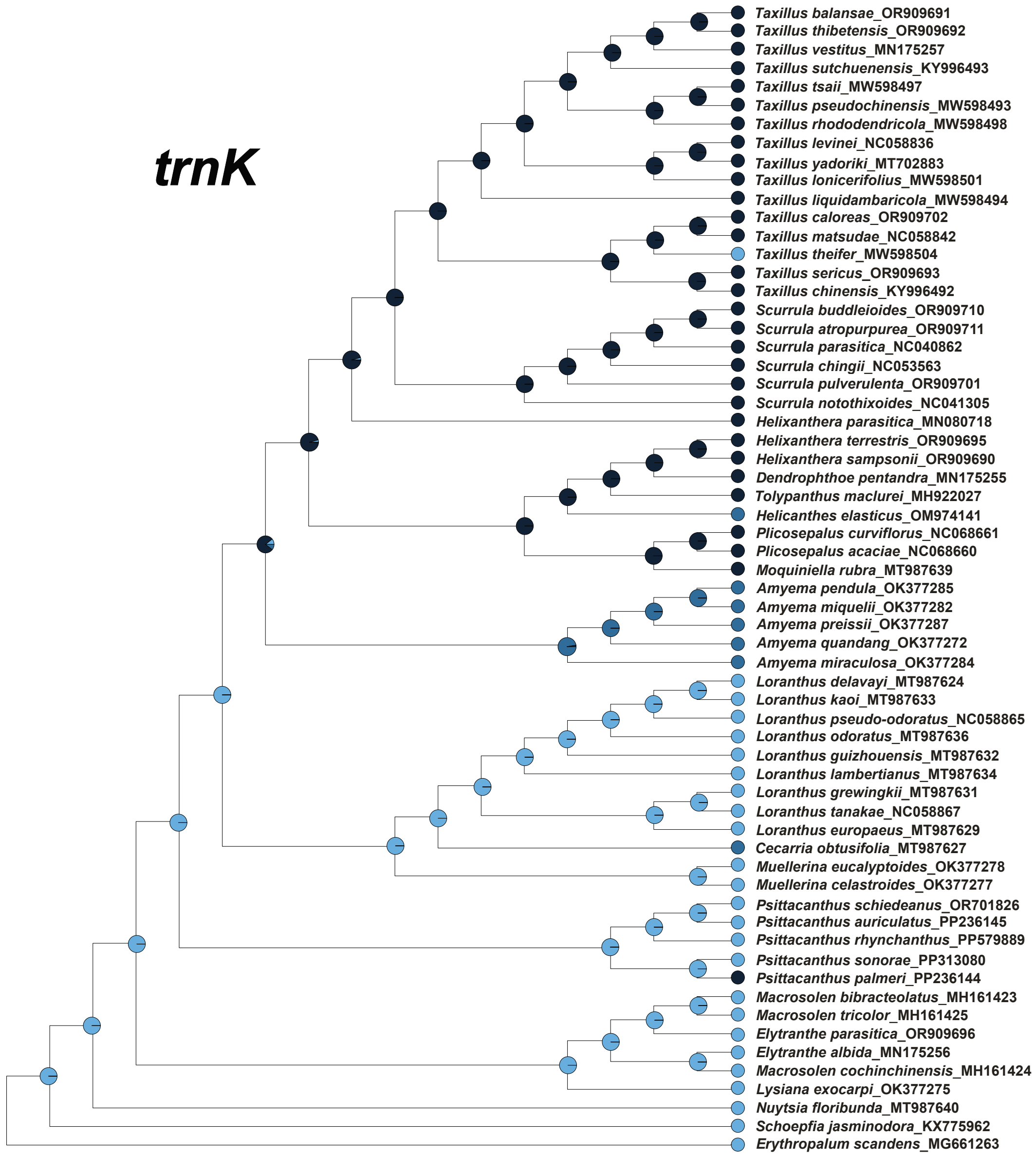
trnK
Taxillus balansae_OR909691
Taxillus thibetensis_OR909692
Taxillus vestitus_MN175257
Taxillus sutchuenensis_KY996493
Taxillus tsaii_MW598497
Taxillus pseudochinensis_MW598493
Taxillus rhododendricola_MW598498
Taxillus levinei_NC058836
Taxillus yadoriki_MT702883
Taxillus lonicerifolius_MW598501
Taxillus liquidambaricola_MW598494
Taxillus caloreas_OR909702
Taxillus matsudae_NC058842
Taxillus theifer_MW598504
Taxillus sericus_OR909693
Taxillus chinensis_KY996492
Scurrula buddleioides_OR909710
Scurrula atropurpurea_OR909711
Scurrula parasitica_NC040862
Scurrula chingii_NC053563
Scurrula pulverulenta_OR909701
Scurrula notothixoides_NC041305
Helixanthera parasitica_MN080718
Helixanthera terrestris_OR909695
Helixanthera sampsonii_OR909690
Dendrophthoe pentandra_MN175255
Tolypanthus maclurei_MH922027
Helicanthes elasticus_OM974141
Plicosepalus curviflorus_NC068661
Plicosepalus acaciae_NC068660
Moquiniella rubra_MT987639
Amyema pendula_OK377285
Amyema miquelii_OK377282
Amyema preissii_OK377287
Amyema quandang_OK377272
Amyema miraculosa_OK377284
Loranthus delavayi_MT987624
Loranthus kaoi_MT987633
Loranthus pseudo-odoratus_NC058865
Loranthus odoratus_MT987636
Loranthus guizhouensis_MT987632
Loranthus lambertianus_MT987634
Loranthus grewingkii_MT987631
Loranthus tanakae_NC058867
Loranthus europaeus_MT987629
Cecarria obtusifolia_MT987627
Muellerina eucalyptoides_OK377278
Muellerina celastroides_OK377277
Psittacanthus schiedeanus_OR701826
Psittacanthus auriculatus_PP236145
Psittacanthus rhynchanthus_PP579889
Psittacanthus sonorae_PP313080
Psittacanthus palmeri_PP236144
Macrosolen bibracteolatus_MH161423
Macrosolen tricolor_MH161425
Elytranthe parasitica_OR909696
Elytranthe albida_MN175256
Macrosolen cochinchinensis_MH161424
Lysiana exocarpi_OK377275
Nuytsia floribunda_MT987640
Schoepfia jasminodora_KX775962
Erythropalum scandens_MG661263

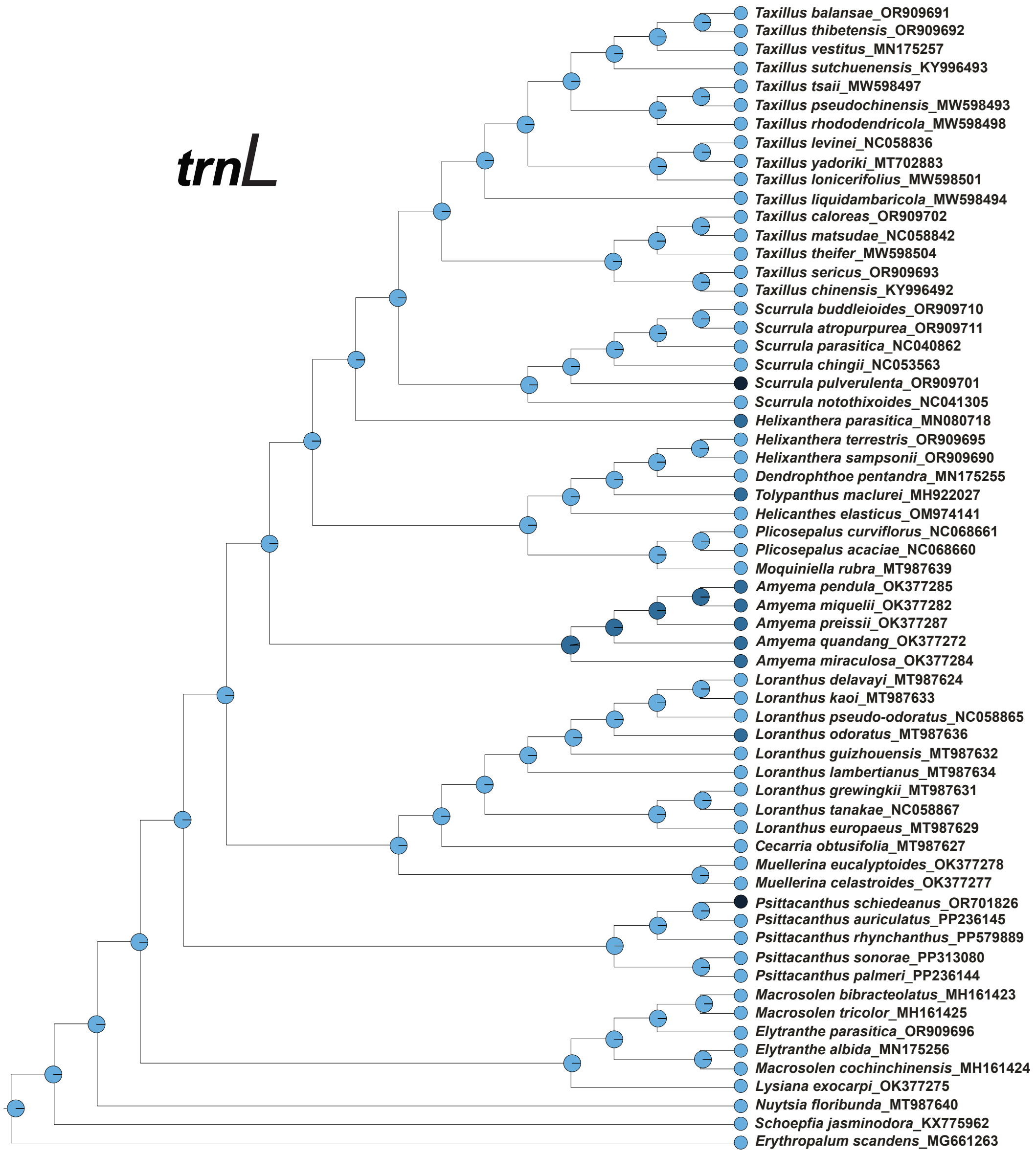
trnL
Taxillus balansae_OR909691
Taxillus thibetensis_OR909692
Taxillus vestitus_MN175257
Taxillus sutchuenensis_KY996493
Taxillus tsaii_MW598497
Taxillus pseudochinensis_MW598493
Taxillus rhododendricola_MW598498
Taxillus levinei_NC058836
Taxillus yadoriki_MT702883
Taxillus lonicerifolius_MW598501
Taxillus liquidambaricola_MW598494
Taxillus caloreas_OR909702
Taxillus matsudae_NC058842
Taxillus theifer_MW598504
Taxillus sericus_OR909693
Taxillus chinensis_KY996492
Scurrula buddleioides_OR909710
Scurrula atropurpurea_OR909711
Scurrula parasitica_NC040862
Scurrula chingii_NC053563
Scurrula pulverulenta_OR909701
Scurrula notothixoides_NC041305
Helixanthera parasitica_MN080718
Helixanthera terrestris_OR909695
Helixanthera sampsonii_OR909690
Dendrophthoe pentandra_MN175255
Tolypanthus maclurei_MH922027
Helicanthes elasticus_OM974141
Plicosepalus curviflorus_NC068661
Plicosepalus acaciae_NC068660
Moquiniella rubra_MT987639
Amyema pendula_OK377285
Amyema miquelii_OK377282
Amyema preissii_OK377287
Amyema quandang_OK377272
Amyema miraculosa_OK377284
Loranthus delavayi_MT987624
Loranthus kaoi_MT987633
Loranthus pseudo-odoratus_NC058865
Loranthus odoratus_MT987636
Loranthus guizhouensis_MT987632
Loranthus lambertianus_MT987634
Loranthus grewingkii_MT987631
Loranthus tanakae_NC058867
Loranthus europaeus_MT987629
Cecarria obtusifolia_MT987627
Muellerina eucalyptoides_OK377278
Muellerina celastroides_OK377277
Psittacanthus schiedeanus_OR701826
Psittacanthus auriculatus_PP236145
Psittacanthus rhynchanthus_PP579889
Psittacanthus sonorae_PP313080
Psittacanthus palmeri_PP236144
Macrosolen bibracteolatus_MH161423
Macrosolen tricolor_MH161425
Elytranthe parasitica_OR909696
Elytranthe albida_MN175256
Macrosolen cochinchinensis_MH161424
Lysiana exocarpi_OK377275
Nuytsia floribunda_MT987640
Schoepfia jasminodora_KX775962
Erythropalum scandens_MG661263

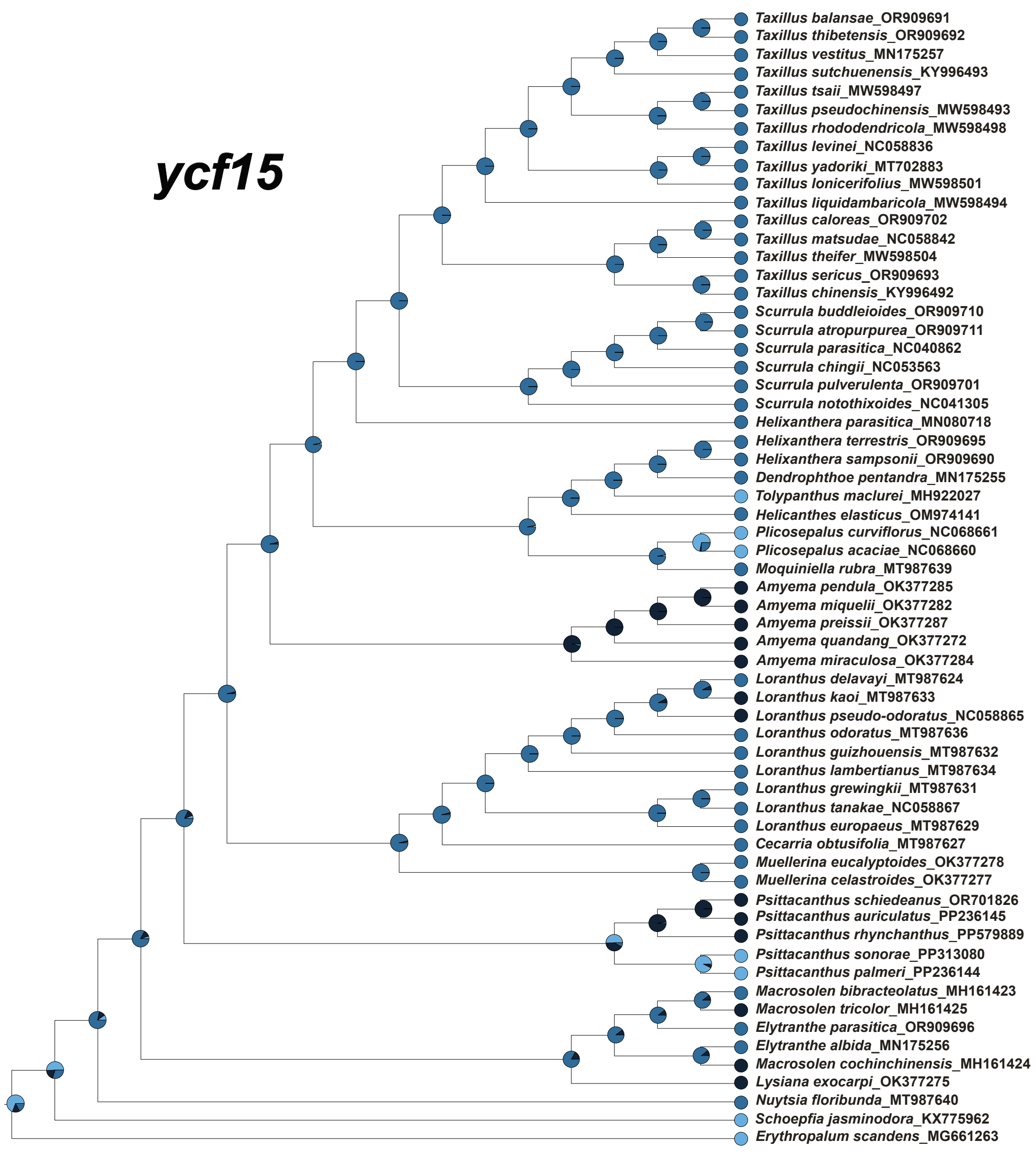
ycf15
Taxillus balansae_OR909691
Taxillus thibetensis_OR909692
Taxillus vestitus_MN175257
Taxillus sutchuenensis_KY996493
Taxillus tsaii_MW598497
Taxillus pseudochinensis_MW598493
Taxillus rhododendricola_MW598498
Taxillus levinei_NC058836
Taxillus yadoriki_MT702883
Taxillus lonicerifolius_MW598501
Taxillus liquidambaricola_MW598494
Taxillus caloreas_OR909702
Taxillus matsudae_NC058842
Taxillus theifer_MW598504
Taxillus sericus_OR909693
Taxillus chinensis_KY996492
Scurrula buddleioides_OR909710
Scurrula atropurpurea_OR909711
Scurrula parasitica_NC040862
Scurrula chingii_NC053563
Scurrula pulverulenta_OR909701
Scurrula notothixoides_NC041305
Helixanthera parasitica_MN080718
Helixanthera terrestris_OR909695
Helixanthera sampsonii_OR909690
Dendrophthoe pentandra_MN175255
Tolypanthus maclurei_MH922027
Helicanthes elasticus_OM974141
Plicosepalus curviflorus_NC068661
Plicosepalus acaciae_NC068660
Moquiniella rubra_MT987639
Amyema pendula_OK377285
Amyema miquelii_OK377282
Amyema preissii_OK377287
Amyema quandang_OK377272
Amyema miraculosa_OK377284
Loranthus delavayi_MT987624
Loranthus kaoi_MT987633
Loranthus pseudo-odoratus_NC058865
Loranthus odoratus_MT987636
Loranthus guizhouensis_MT987632
Loranthus lambertianus_MT987634
Loranthus grewingkii_MT987631
Loranthus tanakae_NC058867
Loranthus europaeus_MT987629
Cecarria obtusifolia_MT987627
Muellerina eucalyptoides_OK377278
Muellerina celastroides_OK377277
Psittacanthus schiedeanus_OR701826
Psittacanthus auriculatus_PP236145
Psittacanthus rhynchanthus_PP579889
Psittacanthus sonorae_PP313080
Psittacanthus palmeri_PP236144
Macrosolen bibracteolatus_MH161423
Macrosolen tricolor_MH161425
Elytranthe parasitica_OR909696
Elytranthe albida_MN175256
Macrosolen cochinchinensis_MH161424
Lysiana exocarpi_OK377275
Nuytsia floribunda_MT987640
Schoepfia jasminodora_KX775962
Erythropalum scandens_MG661263

Figure S2. Phylogenetic net informativeness profiles of 60 protein-coding genes of Loranthaceae estimated. A) Cladogram obtained from 60 plastid protein-coding genes of Loranthaceae. B) Phylogenetic informativeness profiles for 60 protein-coding genes in Loranthaceae, estimated using PhyDesign. The six most informative genes are color-coded, with *ycf1* being the most informative locus. The *x*-axis represents relative time, while the *y*-axis shows net phylogenetic informativeness. C) Phylogenetic informativeness profiles of 60 protein-coding genes in Loranthaceae, estimated using PhyDesign. The *ycf1* gene (the most informative locus) was excluded to highlight the contributions of the remaining five top-informativeness genes. The *x*-axis shows relative time, and the *y*-axis represents net phylogenetic informativeness.

A

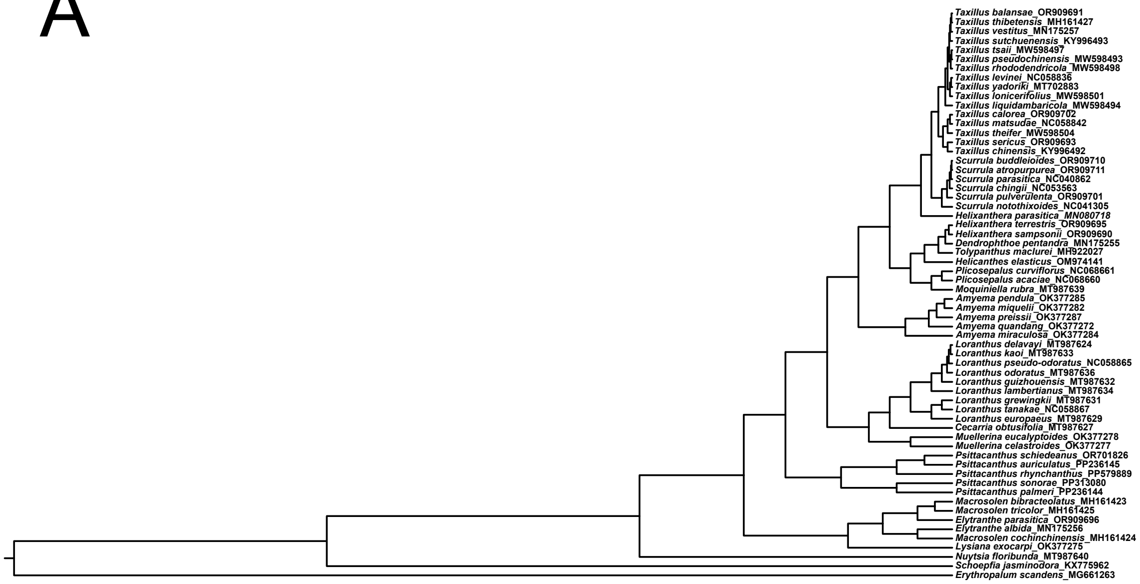

Taxillus balansae_OR909691
Taxillus thibetensis_MH161427
Taxillus vestitus_MN175257
Taxillus sutchuenensis_KY996493
Taxillus tsaii_MW598497
Taxillus pseudochinensis_MW598493
Taxillus rhododendricola_MW598498
Taxillus levinei_NC058836
Taxillus yadoriki_MT702883
Taxillus lonicerifolius_MW598501
Taxillus liquidambaricola_MW598494
Taxillus calorea_OR909702
Taxillus matsudae_NC058842
Taxillus theifer_MW598504
Taxillus sericus_OR909693
Taxillus chinensis_KY996492
Scurrula buddleioides_OR909710
Scurrula atropurpurea_OR909711
Scurrula parasitica_NC040862
Scurrula chingii_NC053563
Scurrula pulverulenta_OR909701
Scurrula notothixoides_NC041305
Helixanthera parasitica_MN080718
Helixanthera terrestris_OR909695
Helixanthera sampsonii_OR909690
Dendrophthoe pentandra_MN175255
Tolypanthus maclurei_MH922027
Helicanthes elasticus_OM974141
Plicosepalus curviflorus_NC068661
Plicosepalus acaciae_NC068660
Moquiniella rubra_MT987639
Amyema pendula_OK377285
Amyema miquelii_OK377282
Amyema preissii_OK377287
Amyema quandang_OK377272
Amyema miraculosa_OK377284
Loranthus delavayi_MT987624
Loranthus kaoi_MT987633
Loranthus pseudo-odoratus_NC058865
Loranthus odoratus_MT987636
Loranthus guizhouensis_MT987632
Loranthus lambertianus_MT987634
Loranthus grewingkii_MT987631
Loranthus tanakae_NC058867
Loranthus europaeus_MT987629
Cecarria obtusifolia_MT987627
Muellerina eucalyptoides_OK377278
Muellerina celastroides_OK377277
Psittacanthus schiedeanus_OR701826
Psittacanthus auriculatus_PP236145
Psittacanthus rhynchanthus_PP579889
Psittacanthus sonorae_PP313080
Psittacanthus palmeri_PP236144
Macrosolen bibracteolatus_MH161423
Macrosolen tricolor_MH161425
Elytranthe parasitica_OR909696
Elytranthe albida_MN175256
Macrosolen cochinchinensis_MH161424
Lysiana exocarpi_OK377275
Nuytsia floribunda_MT987640
Schoepfia jasminodora_KX775962
Erythropalum scandens_MG661263

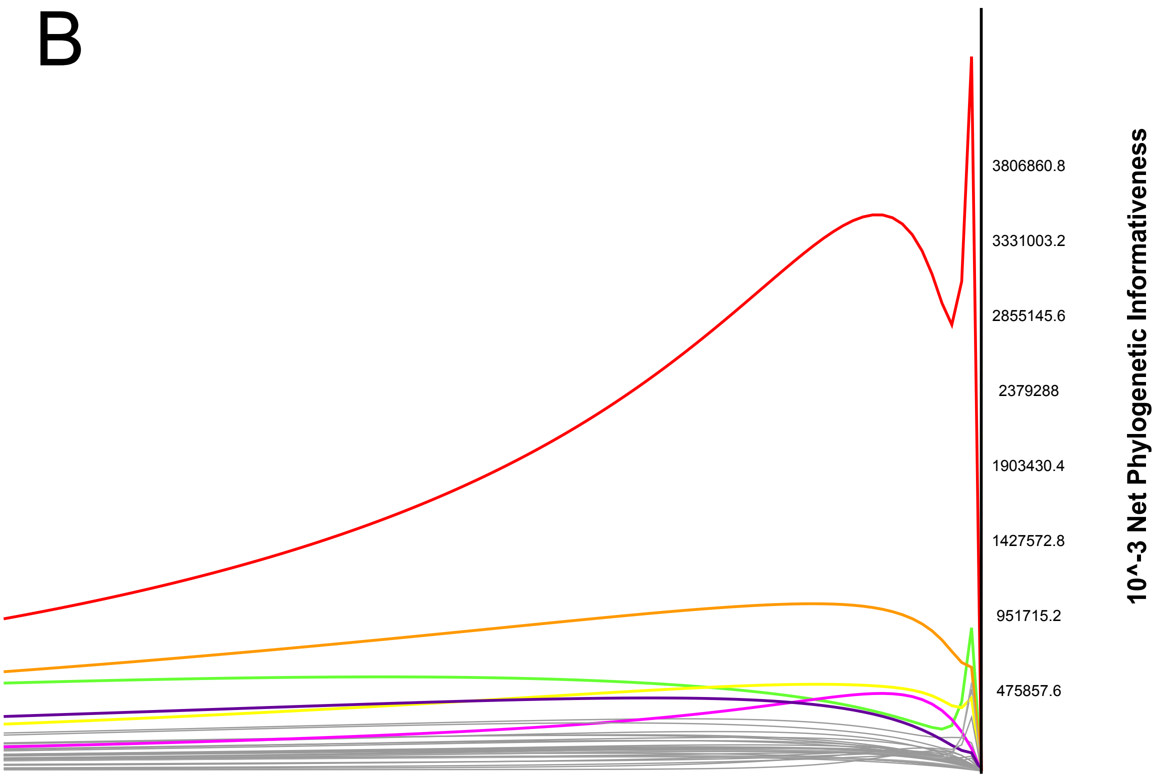
B
3806860.8
3331003.2
2855145.6
2379288
1903430.4
1427572.8
951715.2
475857.6
10^-3 Net Phylogenetic Informativeness

C

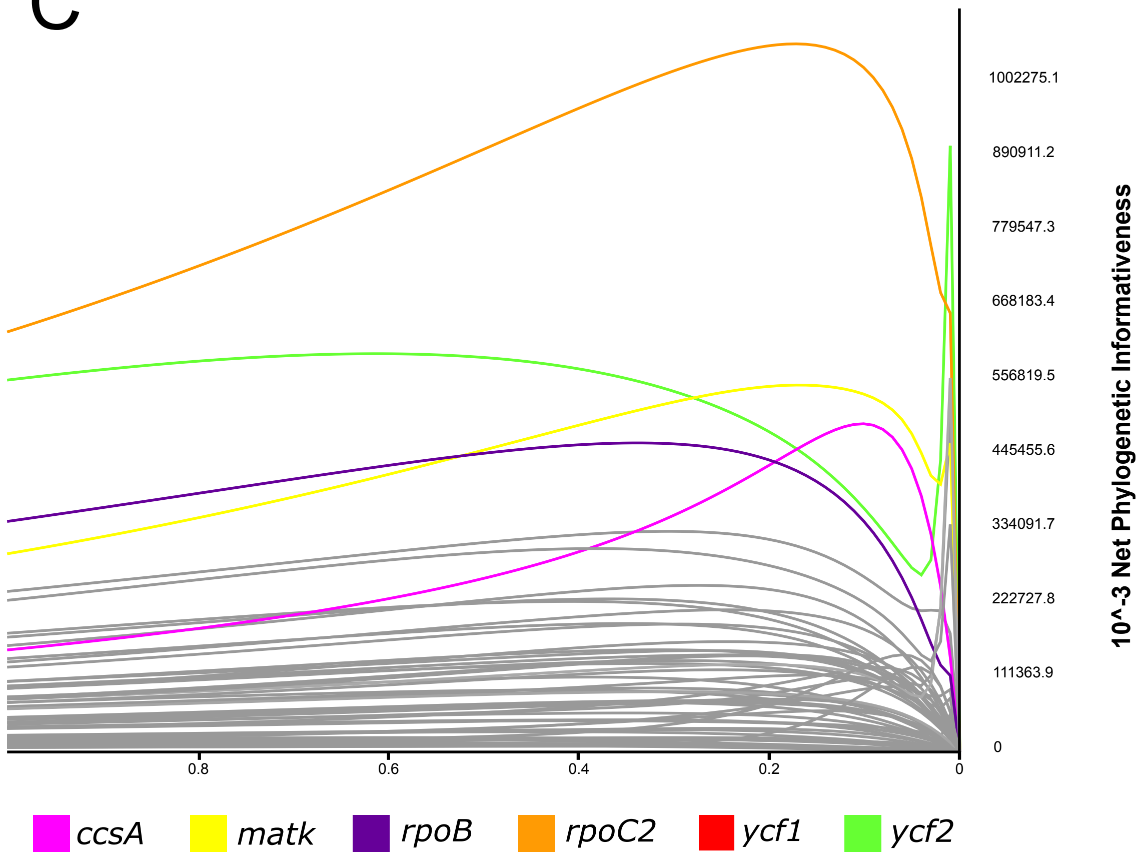

1002275.1
890911.2
779547.3
668183.4
556819.5
445455.6
334091.7
222727.8
111363.9
0
0.8
0.6
0.4
0.2
0
10^-3 Net Phylogenetic Informativeness
ccsA
matk
rpoB
rpoC2
ycf1
ycf2

Figure S3. Comparisons of phylogenetic tree topologies for six genes (*ccsA*, *matK*, *rpoB*, *rpoC2*, *ycf1*, *ycf2*) based on phylogenetic informativeness analysis. Circles above nodes are maximum-likelihood (ML) bootstrap values: black circles correspond to 100 bootstrap values; blue circles represent > 86–99 values; red circles are bootstrap values less than < 85.

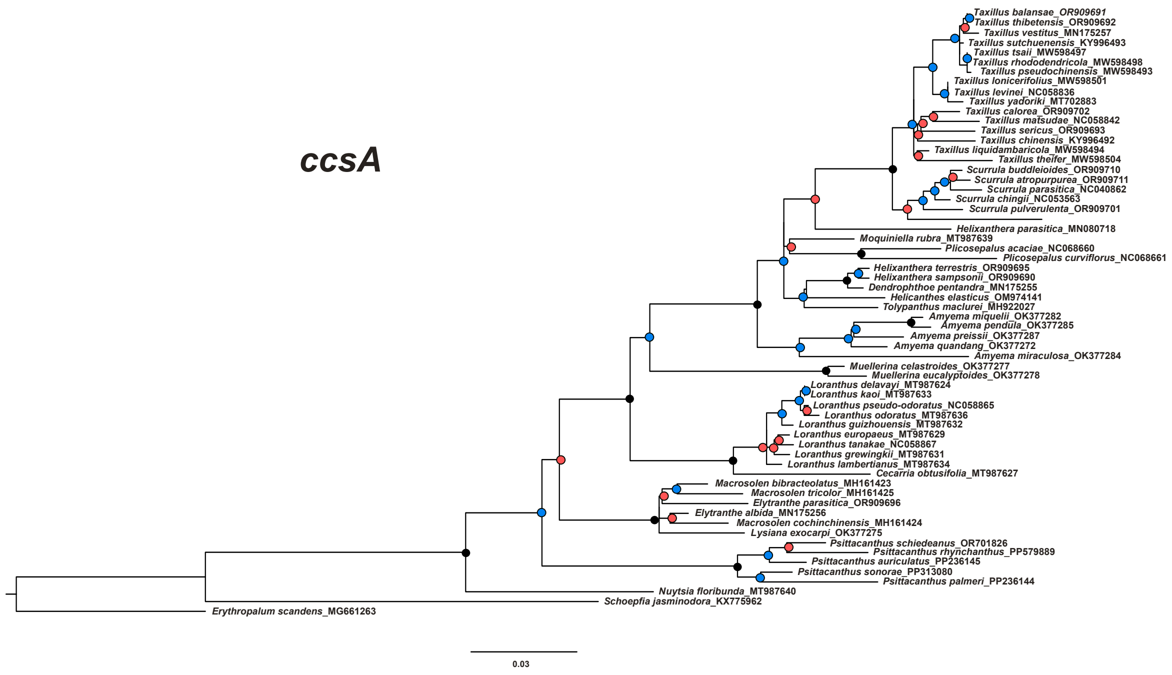
ccsA
Taxillus balansae_OR909691
Taxillus thibetensis_OR909692
Taxillus vestitus_MN175257
Taxillus sutchuenensis_KY996493
Taxillus tsaii_MW598497
Taxillus rhododendricola_MW598498
Taxillus pseudochinensis_MW598493
Taxillus lonicerifolius_MW598501
Taxillus levinei_NC058836
Taxillus yadoriki_MT702883
Taxillus calorea_OR909702
Taxillus matsudae_NC058842
Taxillus sericus_OR909693
Taxillus chinensis_KY996492
Taxillus liquidambaricola_MW598494
Taxillus theifer_MW598504
Scurrula buddleioides_OR909710
Scurrula atropurpurea_OR909711
Scurrula parasitica_NC040862
Scurrula chingii_NC053563
Scurrula pulverulenta_OR909701
Helixanthera parasitica_MN080718
Moquiniella rubra_MT987639
Plicosepalus acaciae_NC068660
Plicosepalus curviflorus_NC068661
Helixanthera terrestris_OR909695
Helixanthera sampsonii_OR909690
Dendrophthoe pentandra_MN175255
Helicanthes elasticus_OM974141
Tolypanthus maclurei_MH922027
Amyema miquelii_OK377282
Amyema pendula_OK377285
Amyema preissii_OK377287
Amyema quandang_OK377272
Amyema miraculosa_OK377284
Muellerina celastroides_OK377277
Muellerina eucalyptoides_OK377278
Loranthus delavayi_MT987624
Loranthus kaoi_MT987633
Loranthus pseudo-odoratus_NC058865
Loranthus odoratus_MT987636
Loranthus guizhouensis_MT987632
Loranthus europaeus_MT987629
Loranthus tanakae_NC058867
Loranthus grewingkii_MT987631
Loranthus lambertianus_MT987634
Cecarria obtusifolia_MT987627
Macrosolen bibracteolatus_MH161423
Macrosolen tricolor_MH161425
Elytranthe parasitica_OR909696
Elytranthe albida_MN175256
Macrosolen cochinchinensis_MH161424
Lysiana exocarpi_OK377275
Psittacanthus schiedeanus_OR701826
Psittacanthus rhynchanthus_PP579889
Psittacanthus auriculatus_PP236145
Psittacanthus sonorae_PP313080
Psittacanthus palmeri_PP236144
Nuytsia floribunda_MT987640
Schoepfia jasminodora_KX775962
Erythropalum scandens_MG661263
0.03

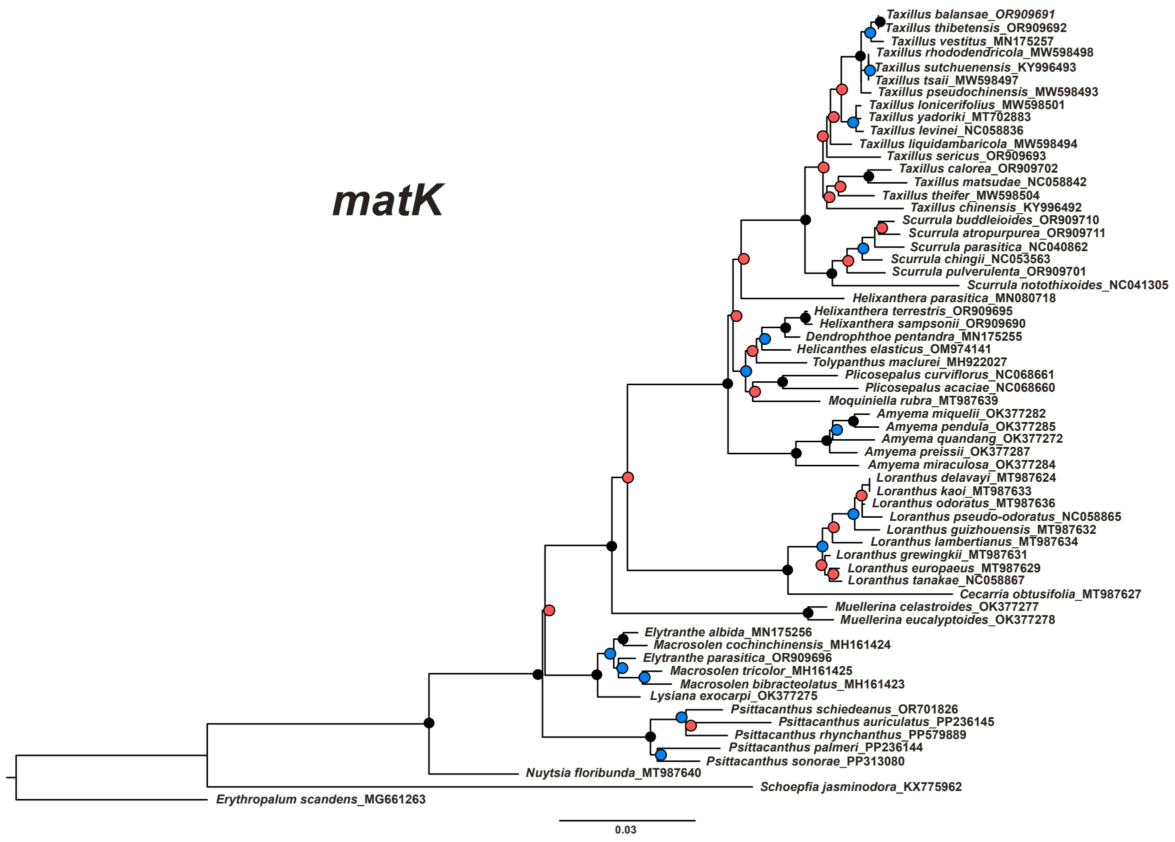
matK
Taxillus balansae_OR909691
Taxillus thibetensis_OR909692
Taxillus vestitus_MN175257
Taxillus rhododendricola_MW598498
Taxillus sutchuenensis_KY996493
Taxillus tsaii_MW598497
Taxillus pseudochinensis_MW598493
Taxillus lonicerifolius_MW598501
Taxillus yadoriki_MT702883
Taxillus levinei_NC058836
Taxillus liquidambaricola_MW598494
Taxillus sericus_OR909693
Taxillus calorea_OR909702
Taxillus matsudae_NC058842
Taxillus theifer_MW598504
Taxillus chinensis_KY996492
Scurrula buddleioides_OR909710
Scurrula atropurpurea_OR909711
Scurrula parasitica_NC040862
Scurrula chingii_NC053563
Scurrula pulverulenta_OR909701
Scurrula notothixoides_NC041305
Helixanthera parasitica_MN080718
Helixanthera terrestris_OR909695
Helixanthera sampsonii_OR909690
Dendrophthoe pentandra_MN175255
Helicanthes elasticus_OM974141
Tolypanthus maclurei_MH922027
Plicosepalus curviflorus_NC068661
Plicosepalus acaciae_NC068660
Moquiniella rubra_MT987639
Amyema miquelii_OK377282
Amyema pendula_OK377285
Amyema quandang_OK377272
Amyema preissii_OK377287
Amyema miraculosa_OK377284
Loranthus delavayi_MT987624
Loranthus kaoi_MT987633
Loranthus odoratus_MT987636
Loranthus pseudo-odoratus_NC058865
Loranthus guizhouensis_MT987632
Loranthus lambertianus_MT987634
Loranthus grewingkii_MT987631
Loranthus europaeus_MT987629
Loranthus tanakae_NC058867
Cecarria obtusifolia_MT987627
Muellerina celastroides_OK377277
Muellerina eucalyptoides_OK377278
Elytranthe albida_MN175256
Macrosolen cochinchinensis_MH161424
Elytranthe parasitica_OR909696
Macrosolen tricolor_MH161425
Macrosolen bibracteolatus_MH161423
Lysiana exocarpi_OK377275
Psittacanthus schiedeanus_OR701826
Psittacanthus auriculatus_PP236145
Psittacanthus rhynchanthus_PP579889
Psittacanthus palmeri_PP236144
Psittacanthus sonorae_PP313080
Nuytsia floribunda_MT987640
Schoepfia jasminodora_KX775962
Erythropalum scandens_MG661263
0.03

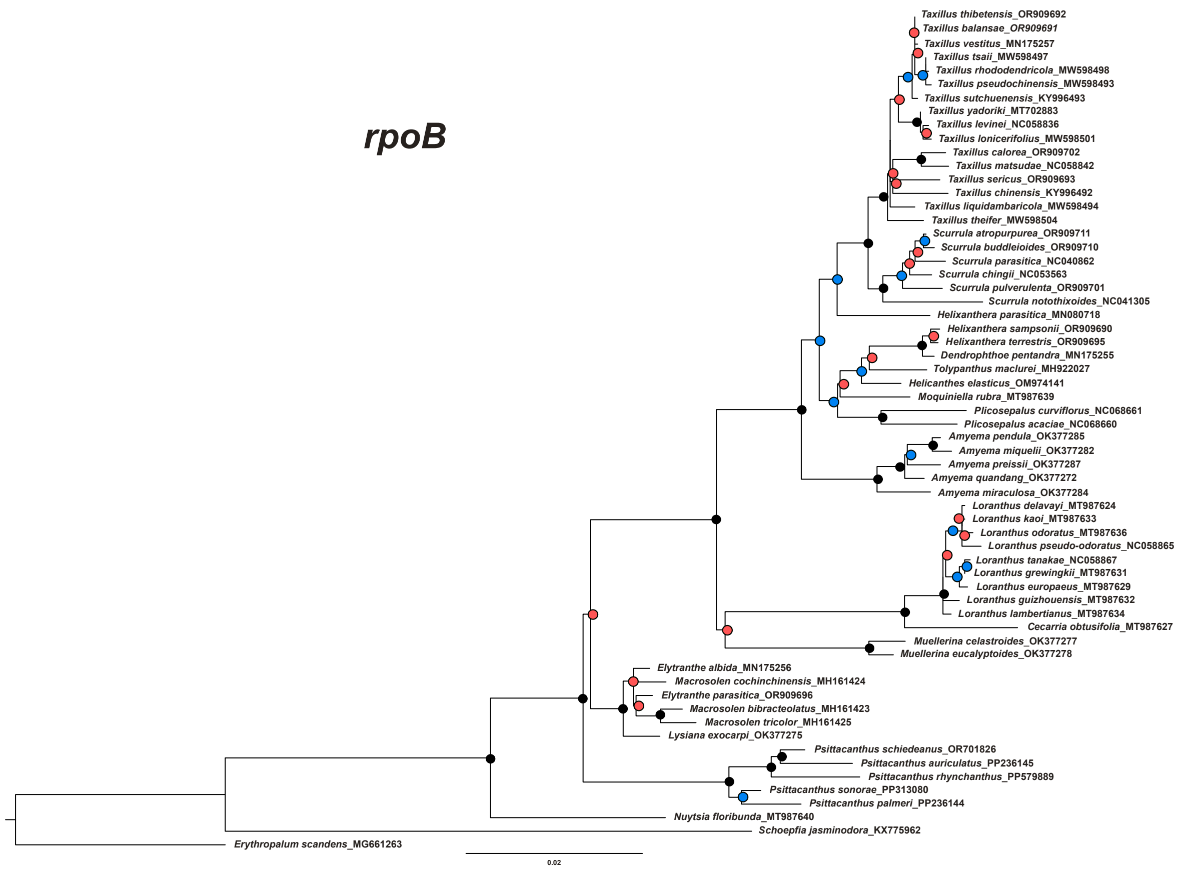
rpoB
Taxillus thibetensis_OR909692
Taxillus balansae_OR909691
Taxillus vestitus_MN175257
Taxillus tsaii_MW598497
Taxillus rhododendricola_MW598498
Taxillus pseudochinensis_MW598493
Taxillus sutchuenensis_KY996493
Taxillus yadoriki_MT702883
Taxillus levinei_NC058836
Taxillus lonicerifolius_MW598501
Taxillus calorea_OR909702
Taxillus matsudae_NC058842
Taxillus sericus_OR909693
Taxillus chinensis_KY996492
Taxillus liquidambaricola_MW598494
Taxillus theifer_MW598504
Scurrula atropurpurea_OR909711
Scurrula buddleioides_OR909710
Scurrula parasitica_NC040862
Scurrula chingii_NC053563
Scurrula pulverulenta_OR909701
Scurrula notothixoides_NC041305
Helixanthera parasitica_MN080718
Helixanthera sampsonii_OR909690
Helixanthera terrestris_OR909695
Dendrophthoe pentandra_MN175255
Tolypanthus maclurei_MH922027
Helicanthes elasticus_OM974141
Moquiniella rubra_MT987639
Plicosepalus curviflorus_NC068661
Plicosepalus acaciae_NC068660
Amyema pendula_OK377285
Amyema miquelii_OK377282
Amyema preissii_OK377287
Amyema quandang_OK377272
Amyema miraculosa_OK377284
Loranthus delavayi_MT987624
Loranthus kaoi_MT987633
Loranthus odoratus_MT987636
Loranthus pseudo-odoratus_NC058865
Loranthus tanakae_NC058867
Loranthus grewingkii_MT987631
Loranthus europaeus_MT987629
Loranthus guizhouensis_MT987632
Loranthus lambertianus_MT987634
Cecarria obtusifolia_MT987627
Muellerina celastroides_OK377277
Muellerina eucalyptoides_OK377278
Elytranthe albida_MN175256
Macrosolen cochinchinensis_MH161424
Elytranthe parasitica_OR909696
Macrosolen bibracteolatus_MH161423
Macrosolen tricolor_MH161425
Lysiana exocarpi_OK377275
Psittacanthus schiedeanus_OR701826
Psittacanthus auriculatus_PP236145
Psittacanthus rhynchanthus_PP579889
Psittacanthus sonorae_PP313080
Psittacanthus palmeri_PP236144
Nuytsia floribunda_MT987640
Schoepfia jasminodora_KX775962
Erythropalum scandens_MG661263
0.02

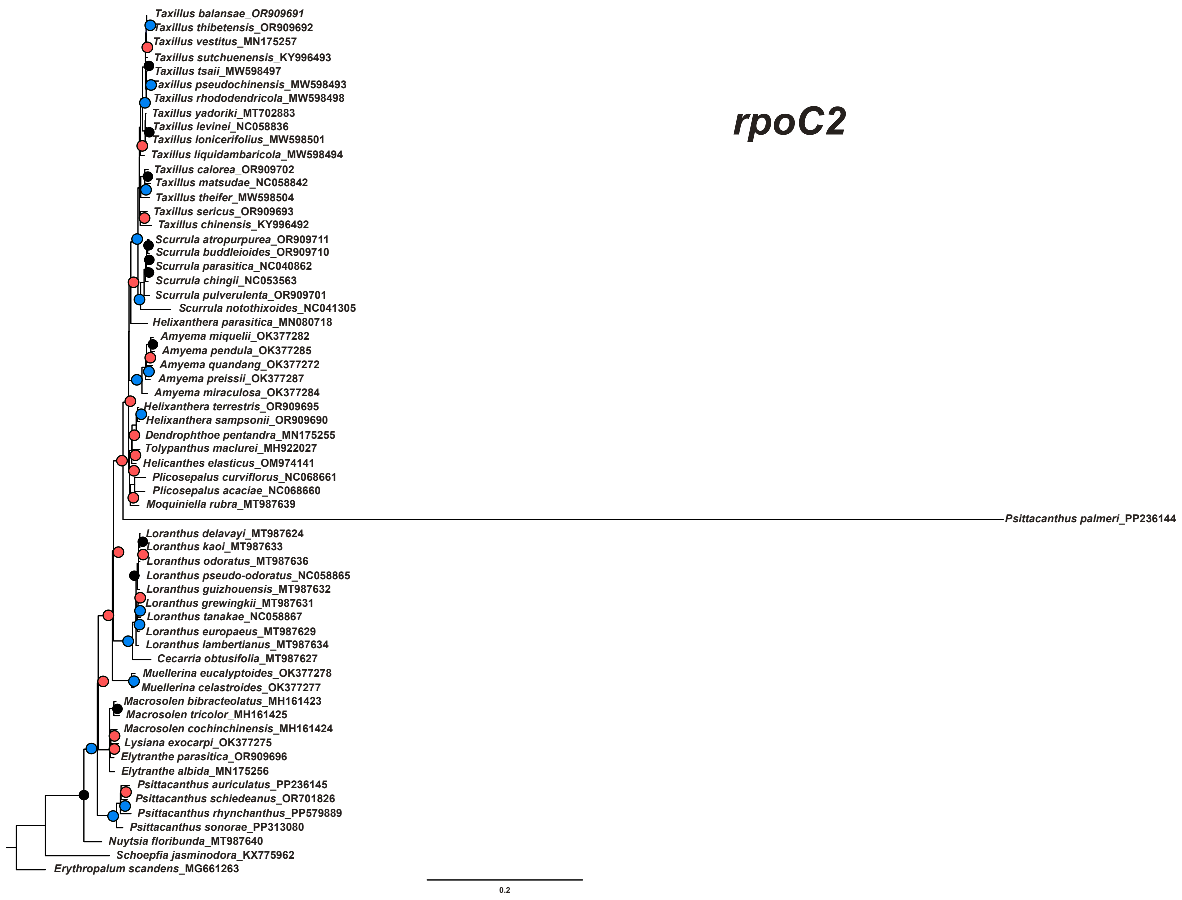
rpoC2
Taxillus balansae_OR909691
Taxillus thibetensis_OR909692
Taxillus vestitus_MN175257
Taxillus sutchuenensis_KY996493
Taxillus tsaii_MW598497
Taxillus pseudochinensis_MW598493
Taxillus rhododendricola_MW598498
Taxillus yadoriki_MT702883
Taxillus levinei_NC058836
Taxillus lonicerifolius_MW598501
Taxillus liquidambaricola_MW598494
Taxillus calorea_OR909702
Taxillus matsudae_NC058842
Taxillus theifer_MW598504
Taxillus sericus_OR909693
Taxillus chinensis_KY996492
Scurrula atropurpurea_OR909711
Scurrula buddleioides_OR909710
Scurrula parasitica_NC040862
Scurrula chingii_NC053563
Scurrula pulverulenta_OR909701
Scurrula notothixoides_NC041305
Helixanthera parasitica_MN080718
Amyema miquelii_OK377282
Amyema pendula_OK377285
Amyema quandang_OK377272
Amyema preissii_OK377287
Amyema miraculosa_OK377284
Helixanthera terrestris_OR909695
Helixanthera sampsonii_OR909690
Dendrophthoe pentandra_MN175255
Tolypanthus maclurei_MH922027
Helicanthes elasticus_OM974141
Plicosepalus curviflorus_NC068661
Plicosepalus acaciae_NC068660
Moquiniella rubra_MT987639
Psittacanthus palmeri_PP236144
Loranthus delavayi_MT987624
Loranthus kaoi_MT987633
Loranthus odoratus_MT987636
Loranthus pseudo-odoratus_NC058865
Loranthus guizhouensis_MT987632
Loranthus grewingkii_MT987631
Loranthus tanakae_NC058867
Loranthus europaeus_MT987629
Loranthus lambertianus_MT987634
Cecarria obtusifolia_MT987627
Muellerina eucalyptoides_OK377278
Muellerina celastroides_OK377277
Macrosolen bibracteolatus_MH161423
Macrosolen tricolor_MH161425
Macrosolen cochinchinensis_MH161424
Lysiana exocarpi_OK377275
Elytranthe parasitica_OR909696
Elytranthe albida_MN175256
Psittacanthus auriculatus_PP236145
Psittacanthus schiedeanus_OR701826
Psittacanthus rhynchanthus_PP579889
Psittacanthus sonorae_PP313080
Nuytsia floribunda_MT987640
Schoepfia jasminodora_KX775962
Erythropalum scandens_MG661263
0.2

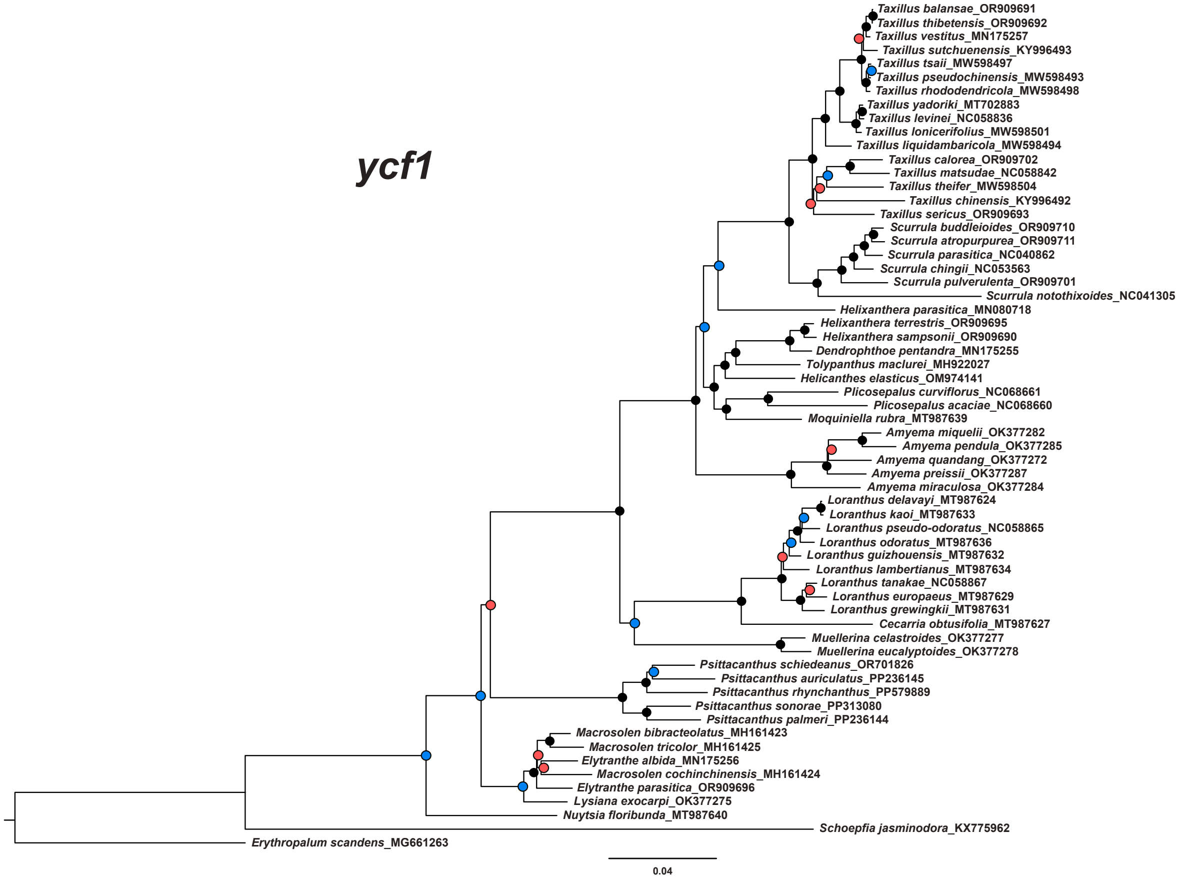
ycf1
Taxillus balansae_OR909691
Taxillus thibetensis_OR909692
Taxillus vestitus_MN175257
Taxillus sutchuenensis_KY996493
Taxillus tsaii_MW598497
Taxillus pseudochinensis_MW598493
Taxillus rhododendricola_MW598498
Taxillus yadoriki_MT702883
Taxillus levinei_NC058836
Taxillus lonicerifolius_MW598501
Taxillus liquidambaricola_MW598494
Taxillus calorea_OR909702
Taxillus matsudae_NC058842
Taxillus theifer_MW598504
Taxillus chinensis_KY996492
Taxillus sericus_OR909693
Scurrula buddleioides_OR909710
Scurrula atropurpurea_OR909711
Scurrula parasitica_NC040862
Scurrula chingii_NC053563
Scurrula pulverulenta_OR909701
Scurrula notothixoides_NC041305
Helixanthera parasitica_MN080718
Helixanthera terrestris_OR909695
Helixanthera sampsonii_OR909690
Dendrophthoe pentandra_MN175255
Tolypanthus maclurei_MH922027
Helicanthes elasticus_OM974141
Plicosepalus curviflorus_NC068661
Plicosepalus acaciae_NC068660
Moquiniella rubra_MT987639
Amyema miquelii_OK377282
Amyema pendula_OK377285
Amyema quandang_OK377272
Amyema preissii_OK377287
Amyema miraculosa_OK377284
Loranthus delavayi_MT987624
Loranthus kaoi_MT987633
Loranthus pseudo-odoratus_NC058865
Loranthus odoratus_MT987636
Loranthus guizhouensis_MT987632
Loranthus lambertianus_MT987634
Loranthus tanakae_NC058867
Loranthus europaeus_MT987629
Loranthus grewingkii_MT987631
Cecarria obtusifolia_MT987627
Muellerina celastroides_OK377277
Muellerina eucalyptoides_OK377278
Psittacanthus schiedeanus_OR701826
Psittacanthus auriculatus_PP236145
Psittacanthus rhynchanthus_PP579889
Psittacanthus sonorae_PP313080
Psittacanthus palmeri_PP236144
Macrosolen bibracteolatus_MH161423
Macrosolen tricolor_MH161425
Elytranthe albida_MN175256
Macrosolen cochinchinensis_MH161424
Elytranthe parasitica_OR909696
Lysiana exocarpi_OK377275
Nuytsia floribunda_MT987640
Schoepfia jasminodora_KX775962
Erythropalum scandens_MG661263
0.04

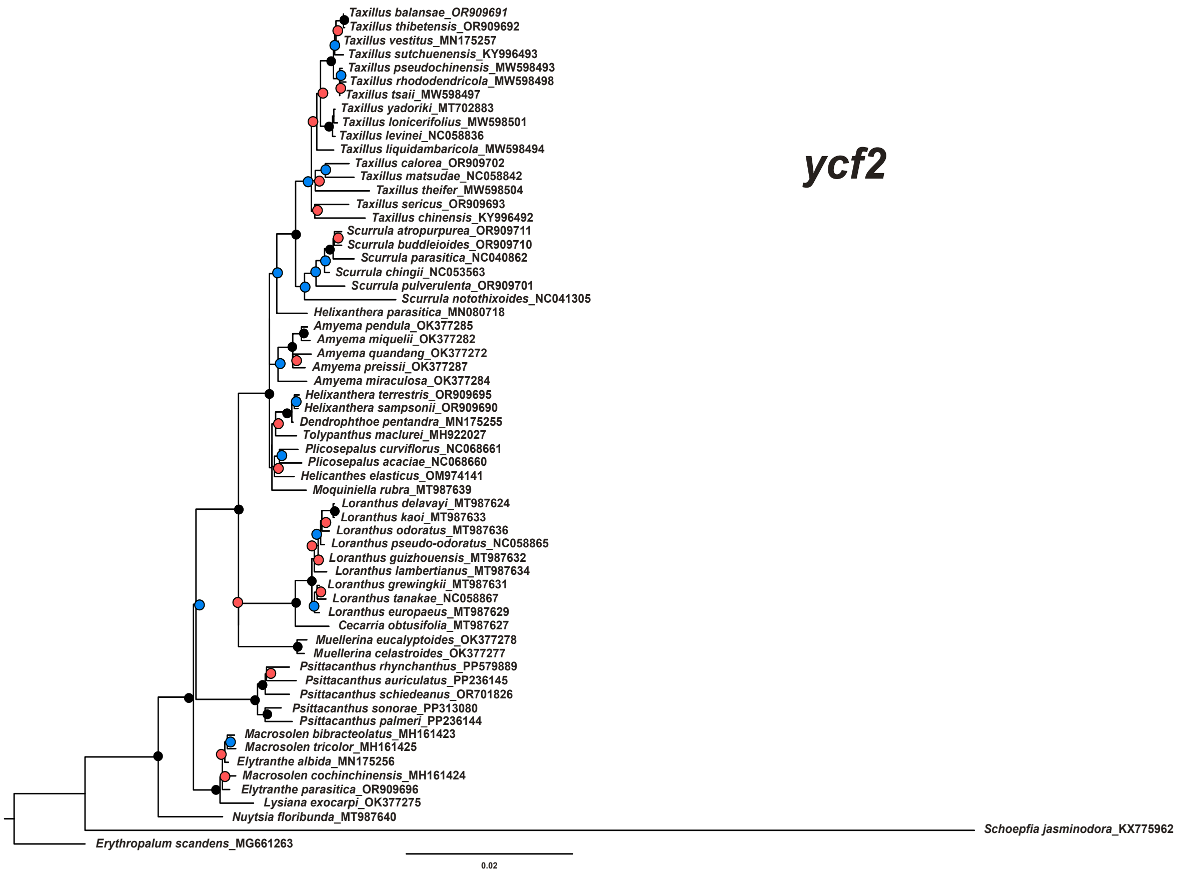

ycf2
Taxillus balansae_OR909691
Taxillus thibetensis_OR909692
Taxillus vestitus_MN175257
Taxillus sutchuenensis_KY996493
Taxillus pseudochinensis_MW598493
Taxillus rhododendricola_MW598498
Taxillus tsaii_MW598497
Taxillus yadoriki_MT702883
Taxillus lonicerifolius_MW598501
Taxillus levinei_NC058836
Taxillus liquidambaricola_MW598494
Taxillus calorea_OR909702
Taxillus matsudae_NC058842
Taxillus theifer_MW598504
Taxillus sericus_OR909693
Taxillus chinensis_KY996492
Scurrula atropurpurea_OR909711
Scurrula buddleioides_OR909710
Scurrula parasitica_NC040862
Scurrula chingii_NC053563
Scurrula pulverulenta_OR909701
Scurrula notothixoides_NC041305
Helixanthera parasitica_MN080718
Amyema pendula_OK377285
Amyema miquelii_OK377282
Amyema quandang_OK377272
Amyema preissii_OK377287
Amyema miraculosa_OK377284
Helixanthera terrestris_OR909695
Helixanthera sampsonii_OR909690
Dendrophthoe pentandra_MN175255
Tolypanthus maclurei_MH922027
Plicosepalus curviflorus_NC068661
Plicosepalus acaciae_NC068660
Helicanthes elasticus_OM974141
Moquiniella rubra_MT987639
Loranthus delavayi_MT987624
Loranthus kaoi_MT987633
Loranthus odoratus_MT987636
Loranthus pseudo-odoratus_NC058865
Loranthus guizhouensis_MT987632
Loranthus lambertianus_MT987634
Loranthus grewingkii_MT987631
Loranthus tanakae_NC058867
Loranthus europaeus_MT987629
Cecarria obtusifolia_MT987627
Muellerina eucalyptoides_OK377278
Muellerina celastroides_OK377277
Psittacanthus rhynchanthus_PP579889
Psittacanthus auriculatus_PP236145
Psittacanthus schiedeanus_OR701826
Psittacanthus sonorae_PP313080
Psittacanthus palmeri_PP236144
Macrosolen bibracteolatus_MH161423
Macrosolen tricolor_MH161425
Elytranthe albida_MN175256
Macrosolen cochinchinensis_MH161424
Elytranthe parasitica_OR909696
Lysiana exocarpi_OK377275
Nuytsia floribunda_MT987640
Schoepfia jasminodora_KX775962
Erythropalum scandens_MG661263
0.02
